# PFN4 is required for manchette development and acrosome biogenesis during mouse spermiogenesis

**DOI:** 10.1242/dev.200499

**Published:** 2022-08-22

**Authors:** Naila Umer, Sharang Phadke, Farhad Shakeri, Lena Arévalo, Keerthika Lohanadan, Gregor Kirfel, Marc Sylvester, Andreas Buness, Hubert Schorle

**Affiliations:** 1 Department of Developmental Pathology, Institute of Pathology, University Hospital Bonn, 53127 Bonn, Germany; 2 Institute for Medical Biometry, Informatics and Epidemiology, Medical Faculty, University of Bonn, 53127 Bonn, Germany; 3 Institute for Genomic Statistics and Bioinformatics, Medical Faculty, University of Bonn, 53127 Bonn, Germany; 4 Institute for Cell Biology, University of Bonn, 53121 Bonn, Germany; 5 Core Facility Mass Spectrometry, Institute of Biochemistry and Molecular Biology, Medical Faculty, University of Bonn, 53115 Bonn, Germany

**Keywords:** Profilin 4, Acrosome biogenesis, PI3K/AKT signaling, *Cis-*Golgi, *Trans-*Golgi, Infertility, *In vitro* fertilization, Morula, Manchette, Spermiogenesis

## Abstract

Profilin 4 (*Pfn4*) is expressed during spermiogenesis and localizes to the acrosome-acroplaxome-manchette complex. Here, we generated PFN4-deficient mice, with sperm displaying severe impairment in manchette formation. Interestingly, HOOK1 staining suggests that the perinuclear ring is established; however, ARL3 staining is disrupted, suggesting that lack of PFN4 does not interfere with the formation of the perinuclear ring and initial localization of HOOK1, but impedes microtubular organization of the manchette. Furthermore, amorphous head shape and flagellar defects were detected, resulting in reduced sperm motility. Disrupted *cis-* and *trans-*Golgi networks and aberrant production of proacrosomal vesicles caused impaired acrosome biogenesis. Proteomic analysis showed that the proteins ARF3, SPECC1L and FKBP1, which are involved in Golgi membrane trafficking and PI3K/AKT pathway, are more abundant in *Pfn4^−/−^* testes. Levels of PI3K, AKT and mTOR were elevated, whereas AMPK level was reduced, consistent with inhibition of autophagy. This seems to result in blockage of autophagic flux, which could explain the failure in acrosome formation. *In vitro* fertilization demonstrated that PFN4-deficient sperm is capable of fertilizing zona-free oocytes, suggesting a potential treatment for PFN4-related human infertility.

## Abstract (precis)

**Summary:** PFN4-deficient male mice exhibit impaired acrosome formation and malformation of the manchette, leading to amorphous sperm head shape, flagellar abnormalities and sterility.

## INTRODUCTION

Fertilization is the process of fusing two gametes for sexual reproduction. Male gametes are produced through spermatogenesis whereby the germ cells undergo a series of various differentiation steps, including condensation of chromatin, development of the manchette, and flagellar and acrosome formation. In mice, formation of the caudal manchette starts during spermiogenesis at step 8, which is present along the base of the developing spermatid nucleus (Long and Cook, 1991; O'Donnell and O'Bryan, 2014). At the beginning of nuclear elongation, the microtubular manchette extends in a posterior direction from the nuclear marginal ring to the cytoplasmic lobe at the distal end, creating a ring-like structure that surrounds the spermatid head underneath the acrosome. Protein synthesis and storage occur in the cytoplasmic lobe and the proteins responsible for the strengthening of the head-tail attachment are transported along manchette microtubules by intra-manchette transport (IMT).

In addition to shaping the sperm head, the manchette is also responsible for trafficking proteins to the developing sperm flagella (Teves et al., 2020). Transport of proteins and vesicles occurs between the cytoplasm and nucleus via nuclear pores, developing centrosomes and sperm flagella. Protein trafficking occurs via IMT using the manchette (Teves et al., 2020). A number of proteins localize to the manchette and contribute to IMT, such as *Hook1* (Mendoza-Lujambio et al., 2002), *Gopc* (Yao et al., 2002), *Pfn3* (Umer et al., 2021), *Spag17* (Kazarian et al., 2018)^,^ *Ift20* (Li et al., 2016), *Lrguk1* (also known as *Lrguk*) (Okuda et al., 2017), *Spef2* (Lehti et al., 2017) and *Cdc42* (Chapin et al., 2001), and mutation results in a mis-shaped sperm head. However, the molecular mechanisms of trafficking proteins responsible for sperm head shaping and flagella development via IMT are not clear (Teves et al., 2020).

During fertilization, the acrosomal reaction takes place, which results in release of the acrosomal content. This is instrumental in breaking down the zona pellucida of the oocyte and enables the penetration and fusion of sperm and oocyte (Sun et al., 2011). Therefore, structural or functional abnormalities of the acrosome are potentially able to interfere with sperm-egg fusion, eventually resulting in sub- or infertility.

Acrosome biogenesis is brought about by several endoplasmic reticulum (ER)- and Golgi-associated proteins and Golgi-mediated vesicle trafficking (Khawar et al., 2019). This vesicle trafficking is in part regulated by the autophagy machinery. Autophagy is the intracellular catabolic process responsible for the degradation and recycling of organelles and cytosolic proteins by autophagosomes (Mizushima and Levine, 2010; Zhu et al., 2019). Autophagy is crucial for the proliferation and differentiation of spermatogonia stem cells, meiotic progression and spermiogenesis. During spermiogenesis, autophagy plays a role in acrosome development, head shaping, flagellum formation and cytoplasmic removal. During the process of autophagy, initially a cup-like structure termed phagophore develops, engulfing cytoplasmic components and then closing as a double-membrane structure named the autophagosome. This process consists of three key steps: initiation, elongation and closure of the membrane (Yang et al., 2017).

Autophagy is a highly specialized and tightly regulated process involving autophagy-related proteins (ATGs). To date, 41 ATGs are known and ATG7 has been demonstrated to be crucial for autophagosome biogenesis (Wang et al., 2014). ATG7 activates LC3 (microtubule-associated protein 1/11-light chain 3, MAP1LC3A), which is transported to Golgi-derived vesicles via phagophores during auto-lysosomal or acrosomal biogenesis. There, the activated LC3 protein facilitates the fusion and transportation of Golgi-derived vesicles in the phagophore-like structures towards the nucleus for acrosome biogenesis (Wang et al., 2014). The major regulator of autophagy, mTOR, is a downstream component of the phosphatidylinositol 3 kinase (PI3K) and AKT-kinase (AKT) pathway (Kaleağasıoğlu et al., 2020). mTOR is the main intracellular autophagic suppressor and is positively regulated by the PI3K/AKT pathway (Li et al., 2018).

The autophagy-related proteins and caspases interplay to regulate autophagy and apoptosis under basal conditions. Recently, the role of caspases in the regulation of autophagy has begun to be described. Core ATGs interact, recruit and activate several caspases by various autophagosomal membranes. Caspases 3 and 9 are markers for apoptosis known to play a role in autophagy (Tsapras and Nezis, 2017). In addition, autophagy is also thought be involved in phagocytosis of the apoptotic cells. It is known that under some physiological and pathological conditions Sertoli cells play a role in phagocytosis of apoptotic germ cells (Dong et al., 2016).

Profilins are small proteins of 15 kDa molecular weight. There are four known profilin genes in mouse and human. The functions of the various profilins seem to have diverged in mammals to either regulate membrane trafficking or regulate the cytoskeleton (Sun et al., 2013). Profilin 1 is ubiquitously expressed (Tariq et al., 2016), localized in the *trans-*Golgi network and required for the formation of constitutive vesicle transport at the *trans-*Golgi network (Dong et al., 2000). Profilin 2, expressed in brain, associates with a number of proteins involved in endocytosis and membrane trafficking (Gareus et al., 2006). The testis-specific PFN3 is localized to the *cis*- and *trans-*Golgi networks and plays a role in autophagy regulation during acrosome biogenesis (Umer et al., 2021).

Profilin 4 (*Pfn4*, PFN4) is highly expressed in testes and localized to the acrosome-acroplaxome-manchette complex (Behnen et al., 2009). Hence, it is tempting to speculate that PFN4 plays a role in acrosome and manchette biogenesis. Of note, *Pfn4* shows only 30% homology to other profilin family members, and it does not encode for the actin and poly-L-proline binding sites that are present in PFN1-3 (Behnen et al., 2009), suggesting that *Pfn4* has functions *in vivo* that are independent of the regulation of actin dynamics.

Here, we generated PFN4*-*deficient mice by CRISPR/Cas9-mediated gene editing in zygotes. PFN4-deficient males show disturbed spermatogenesis, resulting in male infertility. Development of the manchette in *Pfn4^−/−^* mice was greatly disturbed leading to defects in the sperm head and flagella defects impinging on sperm motility. Furthermore, acrosome formation was impaired and abnormalities in the *cis*- and *trans-*Golgi network and disrupted proacrosomal vesicles were observed. Proteomic analyses indicated deregulation of the PI3K/AKT and mTOR/AMPK pathways, suggestive of inhibited autophagy. Taken together, our results show that PFN4 is required for proper manchette and acrosome formation.

## RESULTS

### Generation and phenotypic characterization of PFN4-deficient mouse lines

**Fig. 1.**
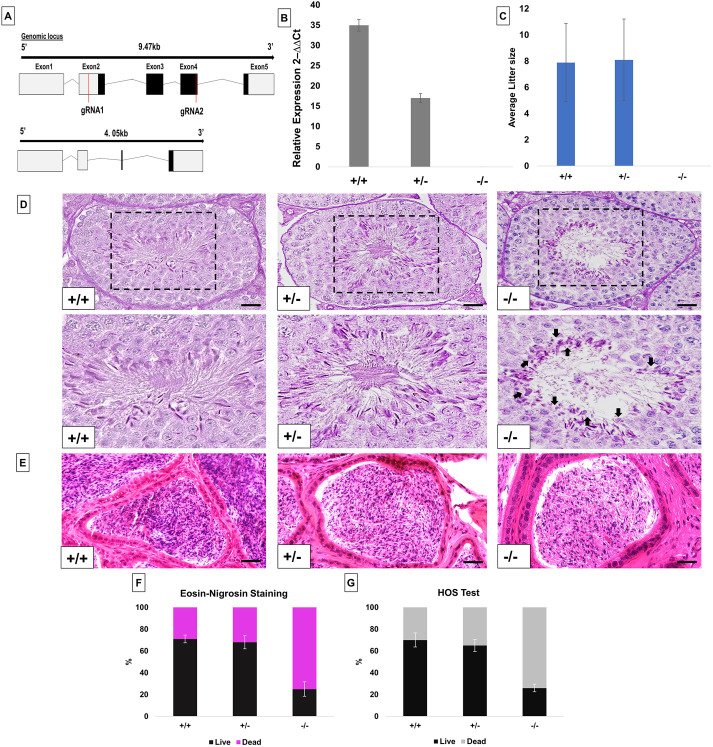
**Generation and characterization of PFN4-deficient mice (*Pfn4*Δa).** (A) Schematic of the *Pfn4* locus and established PFN4-deficient mouse line. Red lines mark positions of the guide RNAs used for gene editing. (B) Validation of PFN4-deficient mice (*Pfn4*Δa). qRT-PCR was performed to check the relative expression of *Pfn4* mRNA in murine testis of WT, *Pfn4*^+/−^ and *Pfn4^−/−^* mice for the *Pfn4*Δa mutation. (C) Mating statistics of WT, *Pfn4*^+/−^ and *Pfn4^−/−^* male mice (*n*=9/genotype). (D) PAS staining on WT, *Pfn4*^+/−^ and *Pfn4^−/−^* testes sections. Dashed boxes indicate the areas shown at higher magnification below. Arrows indicate abnormal head morphology. (E) Hematoxylin and Eosin staining on WT, *Pfn4*^+/−^ and *Pfn4^−/−^* cauda epididymis sections. (F) Quantification of E&N staining (*n*=3 biological replicates/genotype) of mature WT, *Pfn4*^+/−^ and *Pfn4^−/−^* sperm isolated from cauda epididymis. (G) Hypo-osmotic swelling (HOS) test (*n*=3 biological replicates/genotype) of mature WT, *Pfn4*^+/−^ and *Pfn4^−/−^* sperm isolated from cauda epididymis. Error bars represent mean±s.d. Scale bars: 10 μm.

To delete the *Pfn4* allele, two single guide RNAs annealing to exon 2 and exon 4 of the *Pfn4* locus (Fig. 1A) and *Cas9* mRNA were injected into fertilized oocytes. Successful gene editing in the offspring was detected by PCR and confirmed by sequencing (Fig. S1A,B). Two PFN4-deficient mouse lines harboring identical deletions of 5417 bp were established, backcrossed to C57BL/6 mice and named *Pfn4*Δa and *Pfn4*Δb. The deletion causes a frame shift in the PFN4 reading frame, leading to premature translational termination giving rise to a hypothetical truncated protein of 25 amino acids.

For *Pfn4*Δa, real-time quantitative PCR (qRT-PCR) showed that the level of *Pfn4* mRNA is reduced by half in *Pfn4*^+/−^ compared with WT mice. No *Pfn4* transcript was detected in *Pfn4^−/−^* mice confirming the *Pfn4*Δ allele as a null allele (Fig. 1B). Results for *Pfn4*Δb line are shown in the Fig. S1C.

After generating and validating the PFN4-deficient lines, we performed fertility analysis on males from *Pfn4*Δa and *Pfn4*Δb lines. Adult males heterozygous and homozygous for the *Pfn4* deletion mated normally with females as vaginal plugs were clearly seen. Males heterozygous for the *Pfn4* mutation retained normal fertility, with an average litter size comparable to that of wild-type (WT) littermates. This suggests that the reduced level of *Pfn4* mRNA observed in heterozygous males does not affect fertility. In contrast, *Pfn4^−/−^* mice of both *Pfn4*Δa and *Pfn4*Δb lines were infertile as none of the males (*n*=9) produced any pregnancy or offspring (Fig. 1C, *Pfn4*Δa; Fig. S1D, *Pfn4*Δb). Of note, female *Pfn4^−/−^* mice did not show any alterations in fertility (Fig. S1E).

For both PFN4-deficient lines (*Pfn4*Δa, *Pfn4*Δb), body weight (Fig. S1F), and the absolute and relative weight of testes and epididymis in *Pfn4*^−/−^ males were comparable with *Pfn4^+/−^* and WT littermates (Fig. S1F-J). Periodic acid Schiff (PAS) and Hematoxylin and Eosin staining was performed on testes and cauda epididymis and showed that the overall morphology of the seminiferous tubules and cauda epididymis is not affected (Fig. 1D,E), but elongating spermatozoa of *Pfn4^−/−^* mice appeared mis-shapen with seemingly smaller nuclei (Fig. 1D, arrows). These results suggest that loss of *Pfn4* affects spermiogenesis.

In order to assess the viability of spermatozoa, Eosin and Nigrosin (E&N) staining was performed on mature sperm cells isolated from cauda epididymis. E&N staining differentiates live (white heads) from dead (pink heads) sperm (Fig. S1K). In mice, a percentage of 60-80% live sperm is considered normal, 40-60% borderline and less than 40% abnormal. Using males from the *Pfn4*Δa line, E&N staining showed that *Pfn4^+/−^* males mice display a normal percentage of viable sperm whereas in *Pfn4^−/−^* mice an abnormally low percentage of viable sperm (25%) was observed (Fig. 1F). To confirm this, a hypo-osmotic swelling test was performed on cauda epididymal sperm, wherein intact (live) sperm show tail curling (Fig. S1M). The percentage of hypo-osmotic reactive sperm in *Pfn4^−/−^* mice was abnormally low (24%) (Fig. 1G). The results were confirmed with the *Pfn4*Δb animals (Fig. S1L,N). In conclusion, loss of *Pfn4* affects sperm viability. As noted before, we observed that sperm from *Pfn4^−/−^* display an amorphous and smaller head shape (black arrows) (Fig. S1K).

### Loss of PFN4 results in aberrant sperm head morphology

**Fig. 2.**
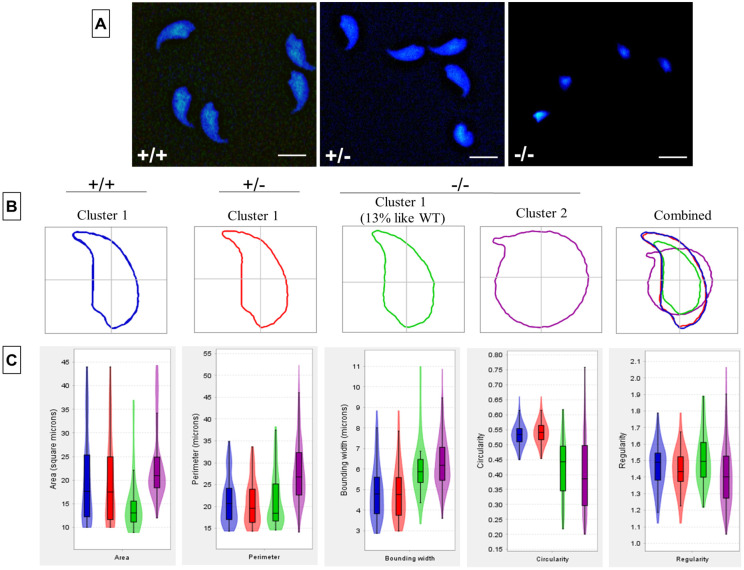
**Sperm head morphology analysis.** Sperm head morphology analysis on WT, *Pfn4*^+/−^ and *Pfn4^−/−^* sperm cells (*n*=3 biological replicates/genotype). In total, 901 nuclei were analyzed: 309 for WT (cluster 1), 276 for *Pfn4^+/−^* (cluster 1) and 541 for *Pfn4^−/−^* (86 sperm nuclei in cluster 1 and 455 nuclei in cluster 2). (A) Sperm cells stained with DAPI. (B) Head morphology of WT, *Pfn4*^+/−^ and *Pfn4^−/−^* sperm cells. (C) Area, perimeter, regularity, bounding width, and circularity of WT, *Pfn4*^+/−^ and *Pfn4^−/−^* sperm cells. Bonferroni's corrected *P*-values were calculated using the non-parametric Mann–Whitney–Wilcoxon test. Error bars show the mean±s.d. Box boundaries indicate consensus nucleus (overall examination of the population). Horizontal lines indicate mean of the respective parameter and error bars indicate coefficient of variability, standard error and standard deviation per parameter.

In order to determine the shape of the sperm head, we next isolated mature sperm from cauda epididymis, and performed nuclear morphology analysis (Fig. 2A). Here, 77% of *Pfn4^−/−^* sperm head showed a dramatically disturbed, irregular/round and smaller head size (Fig. 2B, Cluster 2). Of note, 16% (Cluster 1) of *Pfn4^−/−^* sperm showed a less irregular head shape, which was more comparable to WT and *Pfn4* heterozygous sperm (Fig. 2B). Detailed analysis revealed that in *Pfn4^−/−^* sperm nuclear area, perimeter, regularity, bounding width and circularity were significantly affected compared with WT and *Pfn4^+/−^* sperm (Fig. 2C). Details of parameters are given in Table S1.

### Defective manchette development in *Pfn4^−/−^* mice

**Fig. 3.**
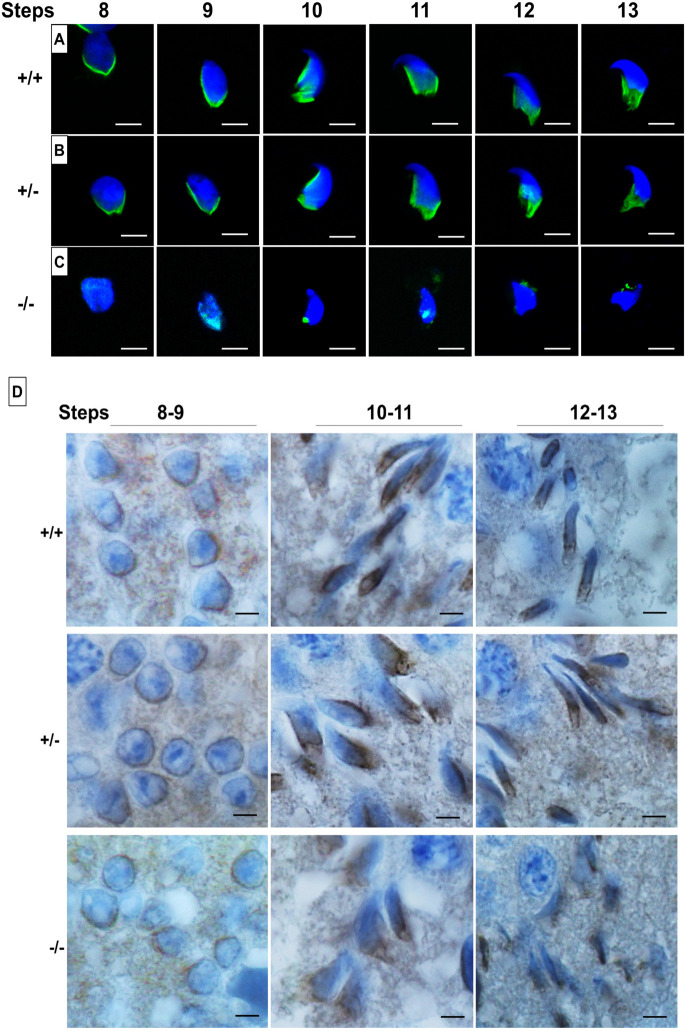
**Manchette staining using α-tubulin and HOOK1 antibodies.** (A-C) Immunofluorescence staining for the manchette using an α-tubulin antibody (green) on a germ cell population isolated from WT (A), *Pfn4*^+/−^ (B) and *Pfn4^−/−^* (C) testes (*n*=3/genotype). Nuclei were stained with DAPI (blue). (D) IHC using an anti-HOOK1 antibody on WT, *Pfn4^+/−^* and *Pfn4^−/−^* testes sections. Scale bars: 20 µm.

The manchette is a temporary microtubular structure that surrounds the sperm nucleus during spermatid elongation and is thought to play a role in shaping of the sperm head (Kierszenbaum and Tres, 2004). As PFN4 is localized to the manchette (Behnen et al., 2009), we investigated whether the loss of PFN4 affects manchette formation. α-Tubulin staining demonstrated that *Pfn4^+/−^* and WT spermatids showed proper manchette development and structural organization, exemplified by a manchette microtubular array forming a tight junction in the caudal region of spermatids (Fig. 3A,B). In contrast, *Pfn4^−/−^* mice showed only marginal staining for α-tubulin, presenting as punctate and dispersed. Hence, the developing *Pfn4^−/−^* spermatids completely lack the typical parallel microtubular array structure of the manchette (Fig. 3C).

In order to understand this defect at the morphological level, we used TEM to analyze the ultrastructure. *Pfn4^−/−^* spermatids showed mislocalization (steps 8-9) and an angular shape (step 10) of the microtubular manchette, whereas in *Pfn4^+/+^* spermatids the microtubular manchette was properly aligned (steps 8-9) and had a crescent shape (step 10) in the middle of the posterior region of the developing spermatid (Fig. S2A). The manchette defect was more prominent in later steps (11-13) of development. In steps 11-13, *Pfn4^−/−^* spermatids lacked the microtubular array structure, and appeared scraggy and rugged in shape, compared with *Pfn4^+/+^* spermatids, in which the microtubular mantle was aligned in arrays (Fig. S2A). These data indicate that deletion of PFN4 disrupts manchette formation, which, in turn, leads to the deformed sperm heads observed in Fig. 2C and Fig. S2A.

Furthermore, we performed immunohistochemistry (IHC) using an α-tubulin antibody on WT, *Pfn4^+/−^* and *Pfn4^−/−^* testis sections. IHC staining was abnormal at steps 8-11 in *Pfn4^−/−^* spermatids (Fig. S2B, stars), compared with WT and *Pfn4^+/−^* testis sections, which showed a properly formed, sickle-shaped microtubular manchette in the posterior region of developing spermatids. Furthermore, at steps 12-16 a complete loss of the manchette and amorphous shape of elongated sperm heads were observed (indicated by stars) in *Pfn4^−/−^* spermatids compared with WT and *Pfn4^+/−^* spermatids (Fig. S2B).

In order to examine further the structural origin of the manchette malformation, we performed IHC using HOOK1 (hook microtubule tethering protein 1) and ARL3 (ADP-ribosylation factor like 3) antibodies on testes sections for all three genotypes to see whether the localization of these proteins is affected in *Pfn4^−/−^* sperm. HOOK1 is localized in the manchette as well in the nuclear ring between the manchette and the nucleus. HOOK1 directs correct positioning and elongation of the microtubular manchette (Mendoza-Lujambio et al., 2002; Schwarz et al., 2017). ARL3 is localized in the manchette and essential for its development (Qi et al., 2013). In addition, ARL3 plays a role in ciliary trafficking and intraflagellar transport (Li et al., 2010). Interestingly in *Pfn4^−/−^* spermatids, HOOK1 is correctly localized in the nuclear ring in steps 8-9 sperm (Fig. 3D). In steps 10-13, HOOK1 expression appears disturbed, reflecting the staining pattern of α-tubulin. This suggests that the manchette starts to form (including the microtubule mantle) with the development of the sperm head, but the manchette structure becomes progressively more abnormal and, in some cases, appears to be absent. Interestingly, ARL3 expression seems to be absent up to steps 12-13, where the ARL3 antibody appears to stain random regions of the sperm heads (Fig. S2C); by contrast, in WT and *Pfn4^+/−^* testes sections, the microtubular manchette is properly aligned from steps 8-13.

### Flagellar defects in *Pfn4^−/−^* sperm

**Fig. 4.**
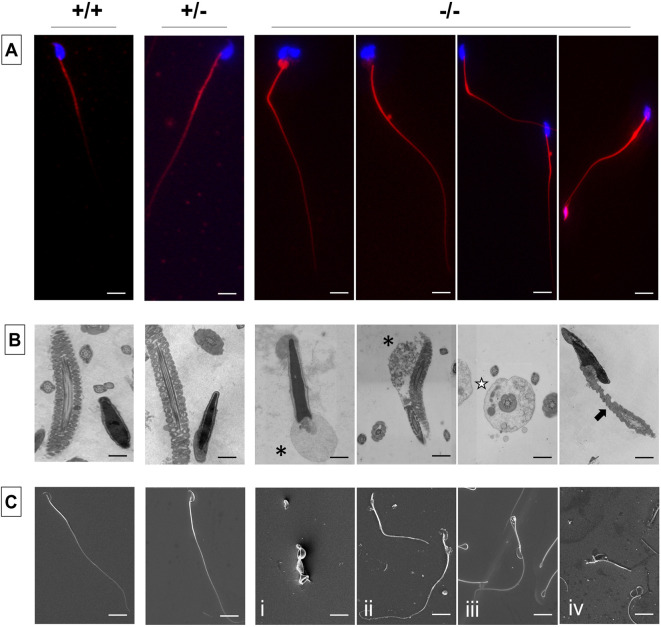
**Flagellar staining and ultrastructural analysis.** (A-C) Flagellar staining and ultrastructural analysis using MitoRed (A), TEM (B) and SEM (C) of WT, *Pfn4*^+/−^ and *Pfn4^−/−^* sperm isolated from the cauda epididymis (*n*=3 biological replicates/genotype). Black asterisks indicate cytoplasmic droplets in the midpiece, white star indicates a defective fibrous sheath, and black arrow indicates mitochondrial structural defects. Scale bars: 10 μm.

It has been reported that during flagellar development sperm tail proteins are also delivered through the manchette, aside from the intraflagellar transport (IFT) (Mochida et al., 1991; Mendoza-Lujambio et al., 2002). Given that loss of *Pfn4* disturbs development of the manchette, we next examined midpiece and flagellar structures in sperm cells. First, Mito Red dye was used to stain the sperm mitochondria in the midpiece of the flagella and DAPI was used to stain sperm heads. Here, *Pfn4^−/−^* sperm revealed numerous deformities including cytoplasmic droplets in the connecting piece, and a bent midpiece and abnormally thick midpiece (Fig. 4A). This suggests that the disturbed manchette seen in *Pfn4^−/−^* males causes the mitochondria abnormalities in sperm flagellum.

Next, transmission electron microscopy (TEM) was performed on cauda epididymis sperm. TEM on *Pfn4^−/−^* sperm revealed disorganized flagellar components such as cytoplasmic droplets in the midpiece (black asterisks), a defective fibrous sheath (white star), and mitochondrial structural defects (black arrow) (Fig. 4B). Scanning electron microscopy (SEM) revealed morphological anomalies of *Pfn4^−/−^* sperm, including coiled flagella (Fig. 4Ci), a shortened thick midpiece (Fig. 4Cii), cytoplasmic droplets in the midpiece (Fig. 4Ciii) and a bent connecting piece (Fig. 4Civ).

### Reduced sperm motility in *Pfn4^−/−^* mice

Flagellar deformities might affect the sperm motility parameters, so we performed computer-assisted semen analysis (CASA), to analyze the swimming properties of *Pfn4^−/−^* sperm. Compared with *Pfn4*^+/+^ and *Pfn4*^+/−^, sperm motility parameters [curvilinear velocity (VCL), straight-line velocity (VSL), average path velocity (VAP) and progressive and total motility] were significantly reduced in *Pfn4^−/−^* sperm (Table S1). These results indicate that abnormalities in sperm flagella lead to reduced overall sperm motility in *Pfn4^−/−^* mice.

### PFN4 is essential for acrosome biogenesis

**Fig. 5.**
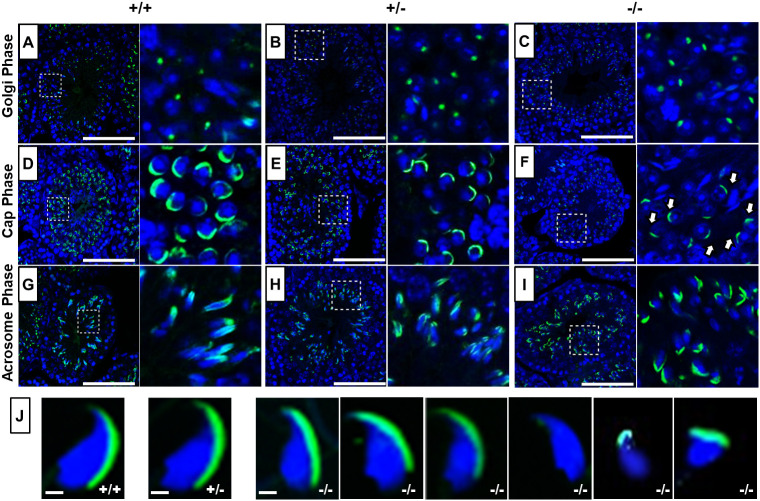
**Acrosome analysis using PNA-FITC fluorescence staining.** (A-I) Immunofluorescence staining for acrosome biogenesis on testes sections and mature sperm of WT, *Pfn4*^+/−^ and *Pfn4^−/−^* mice (*n*=3 biological replicates/genotype). In the Golgi phase, the proacrosomal granule (green) was labeled by PNA-FITC in WT (A), *Pfn4*^+/−^ (B) and *Pfn4^−/−^* (C) round spermatozoa. In the cap phase, acrosomal caps (green) were stained in WT (D), *Pfn4*^+/−^ (E) and *Pfn4^−/−^* (F) round spermatozoa (white arrows show abnormal cap structures). In the acrosomal phase, PNA-FITC-labeled the acrosomal area in WT (G), *Pfn4*^+/−^ (H) and *Pfn4^−/−^* (I) elongated spermatids. Dashed boxes indicate the areas enlarged to the right. (J) Immunofluorescence staining using PNA-FITC (green) on epididymal sperm cells of WT, *Pfn4*^+/−^ and *Pfn4^−/−^* mice (*n*=3/genotype) to show the variety of *Pfn4^−/−^* sperm such as malformed acrosome and abnormal head morphology. Scale bars: 20 μm.

In addition to the manchette, PFN4 is also localized to the acroplaxome (Behnen et al., 2009), suggesting a role in biogenesis/function of the acrosome. In order to examine acrosome development, PNA-FITC fluorescence labeling was performed on testes sections to visualize the different steps of acrosome development. In seminiferous tubules of WT (Fig. 5A) and *Pfn4*^+/−^ (Fig. 5B) male mice, the developing acrosome forms a single homogenous cluster present on the anterior face of nuclei in Golgi-phase spermatids, whereas in *Pfn4*^−/−^ spermatozoa PNA-FITC staining showed a non-uniform shape of the acrosomal vesicle (Fig. 5C). This abnormal acrosome development was further seen in the next step, known as the cap phase. Here, a cap-like covering can be seen on the apical surface of the WT and *Pfn4*^+/−^ spermatozoa (Fig. 5D,E). In *Pfn4^−/−^* sperm cells, the cap-like structures were partially impaired (white arrows) (Fig. 5F). In the acrosomal phase, when elongation and head remodeling of spermatids starts, this abnormal acrosome development was most prominent. In *Pfn4*^−/−^ sperm, the acrosome structure failed to develop into an arrow-like structure (Fig. 5I), as seen in the WT (Fig. 5G) and *Pfn4*^+/−^ sperm (Fig. 5H). These suggest that loss of PFN4 partially impairs biogenesis of the acrosome starting from the first phase (Golgi phase) of acrosome development. Furthermore, PNA-FITC labeling on mature sperm isolated from cauda epididymis of *Pfn4*^−/−^ mice showed malformed acrosomes and aberrant head morphology (Fig. 5J). In addition, PAS staining further confirmed the abnormalities in acrosome development (Fig. S3A).

**Fig. 6.**
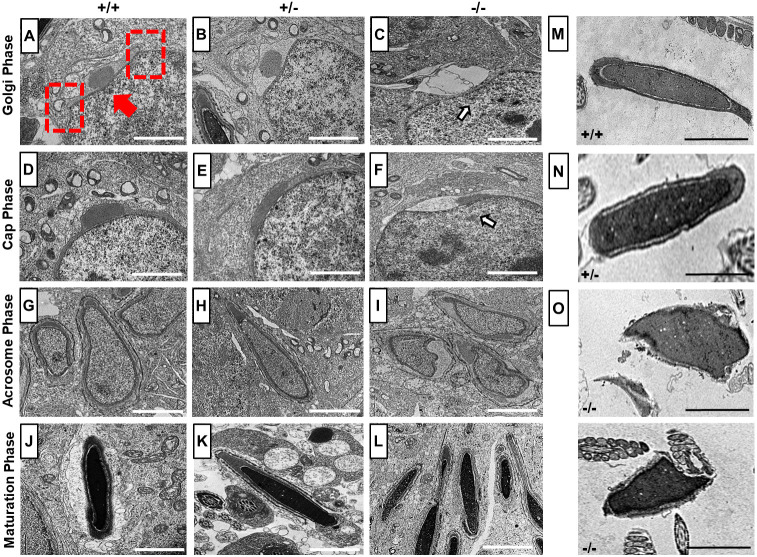
**TEM analysis.** (A-L) Ultrastructural analysis using TEM on testes sections of WT, *Pfn4*^+/−^ and *Pfn4^−/−^* mice (*n*=3 biological replicates/genotype). (A-C) Golgi phase spermatozoa of WT (A), *Pfn4*^+/−^ (B) *Pfn4^−/−^* (C) mice (arrow indicates deformed developing acrosome). Red arrow indicates the acroplaxome, red dashed rectangles indicate the marginal ring. (D-F) Cap phase spermatozoa of WT (D), *Pfn4*^+/−^ (E) and *Pfn4*^−/−^ (F) mice (arrow indicates abnormal cap-like structures). (G-I) Acrosomal phase spermatids of WT (G), *Pfn4*^+/−^ (H) and *Pfn4^−/−^* (I) mice. (J-L) Maturation phase spermatids of WT (J), *Pfn4*^+/−^ (K) and *Pfn4^−/−^* (L) mice. (M-O) Ultrastructural analysis of cauda epididymal sperms of WT (M), *Pfn4*^+/−^ (N) and *Pfn4^−/−^* (O) mice. Scale bars: 2 μm.

Next, TEM was used for ultrastructural analysis of the developing sperm cells. In the Golgi phase, the *trans-*Golgi domain releases proacrosomal vesicles, which fuse to form a single, large and dense acrosomal granule in the center at the marginal ring (red dashed boxes) of the acroplaxome membrane (red arrow) on the nuclear surface as seen in WT and *Pfn4*^+/−^ sperm (Fig. 6A,B). In *Pfn4*^−/−^ mice, the proacrosomal vesicles released from the *trans-*Golgi network did not seem to gather and fuse as a single, large and dense acrosomal vesicle on the apical face of developing spermatozoa (Fig. S3B). This suggests that the acrosomal vesicle formation is greatly delayed retarding acrosome biogenesis (Fig. 6C, arrow indicates large acrosomal vesicle) in subsequent phases. In the cap phase, the proacrosomal vesicle develops and flattens into a cap-like structure over the nucleus in WT (Fig. 6D) and *Pfn4*^+/−^ spermatids (Fig. 6E). However, in *Pfn4*^−/−^ spermatids, the cap-like structure did not spread out over the nucleus, but remained as a pinched granule (Fig. 6F, arrow shows abnormal cap structure). In the acrosomal and maturation phase, this defect became more prominent, and the cap developed further and formed a proper acrosome in WT (Fig. 6G,J) and *Pfn4*^+/−^ (Fig. 6H,K) spermatids, whereas in *Pfn4*^−/−^ sperm proacrosomal vesicles failed to develop into a polarized cap and acrosome (Fig. 6I,L).

To confirm the acrosomal defects, we performed IHC using Sp56 (Zp3r) and acrosin antibodies on testes sections of WT, *Pfn4*^+/−^ and *Pfn4^−/−^* mice. Sp56 and acrosin are markers of acrosomal vesicle and acrosomal matrix, respectively (Xin et al., 2020). Sp56 is detectable as early as the start of acrosome biogenesis starts (Golgi phase) (Kim et al., 2001). IHC showed that Sp56 and acrosin staining were not uniformly distributed in developing acrosomes in *Pfn4^−/−^* compared with WT and *Pfn4^+/−^* testes sections (Fig. S3C,D). These results further validate the observations of impaired acrosome biogenesis in *Pfn4^−/−^* mice.

In order to investigate the acrosomal defect in mature sperm, we used TEM to analyze mature sperm cells isolated from cauda epididymis. Ultrastructural analysis showed a lack of properly formed acrosomes in epididymal sperm of *Pfn4^−/−^* mice (Fig. 6O), whereas WT and *Pfn4*^+/−^ sperm cells showed proper and intact acrosomes (Fig. 6M,N).

### Loss of *Pfn4* results in a fragmented Golgi network

**Fig. 7.**
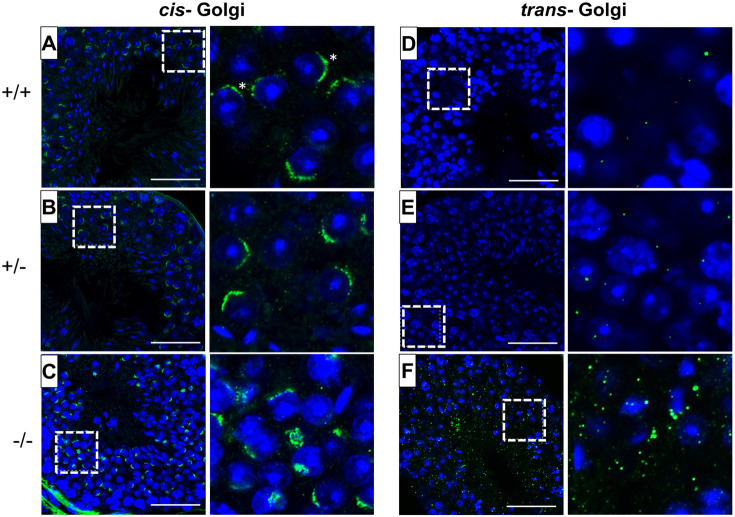
**Fluorescence staining of the Golgi network.** *Cis-* and *trans-*Golgi immunofluorescence staining using GM130 (green) and TGN46 (green) antibodies and nuclei stained with DAPI (blue) on WT, *Pfn4*^+/−^ and *Pfn4^−/−^* (*n*=3/genotype) testes sections. (A-C) WT (A), *Pfn4*^+/−^ (B) and *Pfn4^−/−^* (C) spermatozoa stained for the *cis-*Golgi compartment (asterisks). (D-F) WT (D), *Pfn4*^+/−^ (E) and *Pfn4^−/−^* (F) spermatozoa stained for the *trans-*Golgi network. Dashed boxes indicate the areas enlarged to the right. Scale bars: 50 μm.

The Golgi apparatus is responsible for releasing proacrosomal vesicles for the formation of the acrosome during the Golgi phase of acrosome biogenesis (Tang et al., 1982). We therefore examined the orientation and morphology of the Golgi network domains. Immunofluorescence (IF) staining was performed using GM130 (also known as Golga2) and TGN46 (*trans*-Golgi network protein) as markers for the *cis-* and *trans-*Golgi networks, respectively. GM130 plays a crucial role in vesicle tethering and fusion and in maintaining *cis*-Golgi structural integrity (Tiwari et al., 2019), whereas TGN46 is important for the formation of exocytic vesicles and secretion from the *trans-*part of the Golgi network (Huang et al., 2019). Compared with WT (Fig. 7A,D) and *Pfn4*^+/−^ (Fig. 7B,E) spermatids, IF staining showed defective and fragmented *cis-* (Fig. 7C) and *trans-* (Fig. 7F) Golgi network in *Pfn4*^−/−^ testis sections. In *Pfn4*^−/−^ testis sections, structural disruption and mild mislocalization of the *cis*-Golgi network was observed, and the *trans-*Golgi was fragmented and dispersed throughout the cytoplasm indicating impaired vesicle trafficking and transport, unlike in WT and *Pfn4*^+/−^ spermatids, where *cis-* and *trans*-Golgi were properly formed and aligned. These observations indicate that loss of PFN4 results in disorganization of the Golgi sub-domains, leading to malformed proacrosomal vesicles that seem to impair acrosome biogenesis.

Furthermore, we checked the localization of COPA (coatomer protein complex subunit α), PICK1 (protein interacting with C kinase 1) and GOPC (Golgi associated PDZ and coiled-coil motif containing) using IHC on testes sections of WT, *Pfn4*^+/−^ and *Pfn4^−/−^* mice. COPA is essential for cargo transport of Golgi-derived proteins (Lepelley et al., 2020), and PICK1 and GOPC play an important role in vesicle release from Golgi network and is further associated with proacrosomal granule formation (Xiao et al., 2009). Our IHC showed that COPA is mislocalized (arrowheads) in *Pfn4*^−/−^ spermatids, indicating disrupted vesicle transport for acrosome biogenesis, whereas in WT and *Pfn4*^+/−^ developing spermatids COPA is properly localized in the Golgi apparatus (black arrows) (Fig. S4A). IHC using anti-PICK1 and anti-GOPC antibodies showed mislocalization of Golgi was observed in *Pfn4*^−/−^ testes sections, whereas in WT and *Pfn4*^+/−^ testes sections (black arrows) staining showed proper Golgi localization (Fig. S4B,C). These results further indicate that vesicle trafficking is affected in *Pfn4^−/−^* mice, ultimately resulting in abnormal formation of proacrosomal granules.

### Disruption of PI3K/AKT and AMPK/mTOR signaling pathways leads to inhibited autophagy in *Pfn4*^−/−^ mice

In order to gain insight into the molecular mechanisms affecting the process of Golgi vesicle trafficking, liquid chromatography-mass spectrometry (LC-MS) was performed on protein lysates extracted from testes. A principal component analysis (PCA) performed on the top 5% proteins with highest variance across samples showed an overall biological difference between all three genotypes (WT, *Pfn4^+/−^* and *Pfn4^−/−^*) and the knockout replicates clustered apart from the controls (Fig. S5A).

Eleven proteins were differentially abundant when comparing *Pfn4^−/−^* samples with *Pfn4^+/−^* samples; however, significantly differentially abundant proteins were not detected in *Pfn4^−/−^* samples compared with the WT (Fig. S5B). The effect of PFN4 deficiency is likely restricted to the round spermatid stages. Because proteins were extracted from the whole testis, the samples include proteins from a very heterogeneous cell population. This could lead to difficulties in detecting signals of differential abundance due to PFN4 deficiency.

**Fig. 8.**
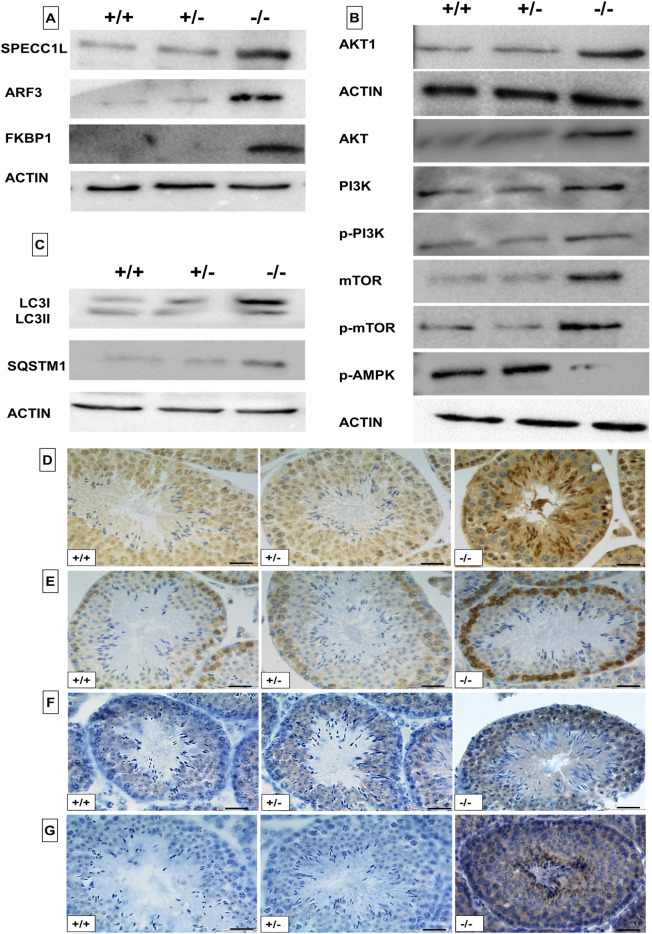
**Analysis of autophagy regulators.** Western blot analysis and immunohistochemical staining on WT, *Pfn4*^+/−^ and *Pfn4^−/−^* mice (*n*=3/genotype). (A) Western blot using SPECC1L, ARF3 and FKBP1 antibodies on protein extracts from WT, *Pfn4*^+/−^ and *Pfn4^−/−^* testes. (B) Western blot using AKT, AKT1, PI3K, p-PI3K, mTOR, p-mTOR and p-AMPK antibodies on protein extracts from WT, *Pfn4*^+/−^ and *Pfn4^−/−^* testes. (C) Western blot using LC3I/II and SQSTM1 antibodies on protein extracts from WT, *Pfn4*^+/−^ and *Pfn4^−/−^* testes. (D-G) IHC using LC3I/II (D), SQSTM1 (E), AKT1 (F) and ARF3 (G) antibodies on WT, *Pfn4*^+/−^ and *Pfn4^−/−^* testes sections. Scale bars: 50 μm.

However, visualization of the data by heatmap showed that in *Pfn4^−/−^* extracts three proteins ARF2:3 (ADP-ribosylation factor), SPECC1L (sperm antigen with calponin homology and coiled-coil domains 1 like) and FKBP1A (FKBP prolyl isomerase 1a) were moderately more abundant compared with *Pfn4^+/+^* and *Pfn4^+/−^* (Fig. S5C)*.* These proteins are involved in the *trans-*Golgi network, vesicle tethering and autophagy regulation. To validate these results, western blots on protein lysates from testes were performed. Here, enhanced levels of ARF3, SPECCL1L and FKBP1 were detected in *Pfn4^−/−^* testes (Fig. 8A). Furthermore, pathways for autophagy, PI3K/AKT and mTOR signaling, protein processing in the ER and Golgi vesicle transport were enriched in *Pfn4^−/−^* (Fig. S5D). Next, we aimed to validate this upregulation of P13K/AKT and AMPK/mTOR signaling in PFN4-deficient mice. PI3K/AKT and mTOR/AMPK regulate autophagic flux and are negative and positive regulators of autophagy, respectively. Western blot analyses showed increased protein levels for AKT (total), AKT1 (phosphorylated), PI3K (total) and phospho-P13K (phosphorylated), mTOR and phospho-mTOR, whereas the level of phospho-AMPKα was reduced in *Pfn4^−/−^* testes (Fig. 8B). These results together indicate that loss of PFN4 affects the autophagy-related signaling pathways PI3K/AKT and mTOR as well as protein processing in the ER–*trans-*Golgi network (ER-TGN).

Next, we investigated whether the altered levels of PI3K/AKT and mTOR/AMPK affect formation of the autophagosome. Therefore, levels of LC3-I/II, a marker for autophagy activation, and SQSTM1 (also known as p62), a marker for autophagic/lysosomal degradation, were analyzed by western blot. The immunoblot showed elevated protein levels of LC3I/II and SQSTM1/p62 in PFN4-deficient testis (Fig. 8C). Furthermore, increased protein levels of LC3II (Fig. 8D), SQSTM1 (Fig. 8E), AKT1 (Fig. 8F) and ARF3 (Fig. 8G) were also confirmed by IHC. Quantification of the western blots showed increased protein levels for LC3BI/II, SQSTM1, ARF3, FKBP1, SPECC1L, AKT1, AKT, mTOR, p-mTOR, p-PI3K and PI3K, and decreased levels for AMPK (Fig. S5E). These results suggest that the autophagic flux is impaired leading to inhibition of autophagy.

### The acrosomal reaction is significantly reduced in *Pfn4^−/−^* sperm

**Fig. 9.**
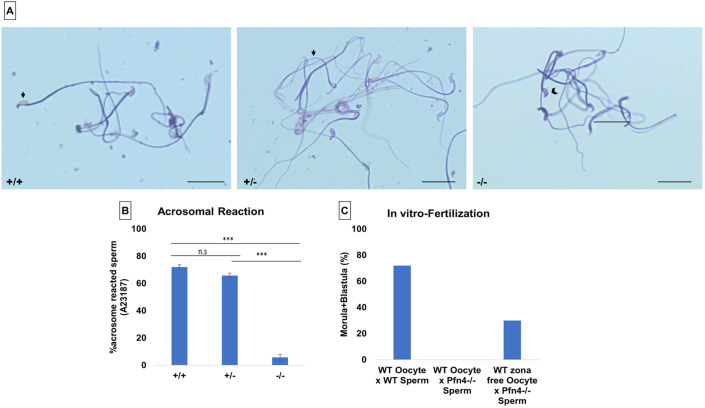
**Acrosomal reaction and *in vitro* fertilization.** Calcium ionophore-induced acrosomal reaction in WT, *Pfn4*^+/−^ and *Pfn4^−/−^* sperm cells (*n*=3/genotype). (A) Coomassie-stained sperm cells of WT, *Pfn4*^+/−^ and *Pfn4^−/−^*. Black arrows indicate that a successful acrosomal reaction took place. Black arrowhead indicates a crescent-shaped acrosome on a sperm head, indicating that the acrosomal reaction did not take place. Scale bars: 20 μm. (B) Percentage of acrosomal-reacted sperm WT, *Pfn4*^+/−^ and *Pfn4^−/−^* (*n*=3 biological replicates/genotype). ***P<0.0005, one-tailed, paired Student's *t*-test. *P*-value relates to acrosomal-reacted WT and *Pfn4*^+/−^ sperm compared with *Pfn4^−/−^* sperm. (C) Percentage of oocytes developing into blastocysts after fertilization of oocytes with WT and *Pfn4^−/−^* sperm and zona-free oocytes with *Pfn4^−/−^* sperm (*n*=1/genotype).

As a result of the impaired acrosome biogenesis, we expected the acrosomal reaction to be affected. To induce the acrosomal reaction, we used A23187 and Coomassie staining to differentiate the acrosome-reacted sperm (in which the acrosome is not present on the sperm head; Fig. 9A, black arrows) from the non-acrosomal-reacted sperm [in which the acrosome is detectable as a crescent-like shape (dark blue) on the sperm head; Fig. 9A, black arrowhead]. Bright-field microscopy was used to assess 200 spermatozoa in triplicate for each genotype. The acrosomal reaction was assessed by the absence of the acrosome (a dark-blue crescent shape on the sperm head). Upon exposure to A23187, more than 72% of sperm from WT mice and 65% of sperm from *Pfn4*^+/−^ mice showed acrosomal exocytosis, whereas only 5-6% sperm of the *Pfn4*^−/−^ mice underwent the acrosome reaction (Fig. 9B). This finding demonstrates that sperm of *Pfn4^−/−^* males display a significantly reduced acrosome reaction.

### PFN4-deficient sperm are able to fertilize zona-free oocytes

The acrosome contains digestive enzymes that degrade the zona pellucida and enable fusion of the sperm with the egg (Saldívar-Hernández et al., 2015). Because PFN4-deficient sperm display defects in acrosome function, we performed *in vitro* fertilization on oocytes with and without the zona pellucida using *Pfn4^−/−^* sperm. Although *Pfn4^−/−^* sperm were not capable of fertilizing oocytes with zonae, using zona-free oocytes resulted in successful fertilization with 30% of oocytes developing to the morula/blastocyst stage (Fig. 9C). In comparison, 70% of oocytes were successfully fertilized using WT sperm. This indicates that the acrosome defects in *Pfn4^−/−^* sperm contribute to male infertility in PFN4-deficient mice.

### Actin cytoskeletal organization is normal in *Pfn4^−/−^* seminiferous epithelium

In order to check whether loss of PFN4 also affects actin cytoskeletal organization in testicular seminiferous tubules, we performed phalloidin staining on testes sections of WT, *Pfn4^+/−^* and *Pfn4^−/−^* mice. The actin cytoskeleton is important for migration of germ cells from the apical to basal regions of seminiferous tubules, and maintenance of cell–cell (Sertoli–spermatid) interactions (Kanatsu-Shinohara et al., 2008). Interestingly, we found that the dynamic actin cytoskeleton is comparable for all three genotypes (Fig. S6). This result suggests that loss of PFN4 does not impede actin assembly in seminiferous epithelium of testes.

### Apoptosis and phagocytosis are not observed in *Pfn4^−/−^* mice

To determine whether apoptosis was induced in *Pfn4^−/−^* germ cells, IHC was performed for the apoptotic markers cleaved caspase 3, cleaved caspase 9 and active caspase 3. Upon apoptosis induction, caspases are actively transported into the nucleus resulting in cell death. Interestingly, IHC revealed no indication of germ cell death in *Pfn4^−/−^* testes, with staining comparable to WT and *Pfn4^−/−^* sections (Fig. S7A). Furthermore, we performed TEM analysis on testes sections to detect phagocytosis of germ cells by Sertoli cells. Surprisingly, phagocytosis was not observed in *Pfn4^−/−^* Sertoli cells, which is comparable to WT and *Pfn4^+/−^* sections (Fig. S7B).

## DISCUSSION

Here, we have established and analyzed mouse lines deficient in PFN4 and demonstrate that lack of PFN4 leads to male infertility. We show that PFN4-null spermatids display malformed formation of the manchette and flagella. Disturbed manchette development is the main cause of abnormal formation of sperm heads and flagellar defects, which lead to reduced sperm motility. We further reveal that acrosome biogenesis is impaired. Defects in acrosome biogenesis seems to originate from a malfunctioning Golgi network, which is incapable of forming a proper acrosomal vesicle. Proteomic analysis revealed an enrichment of the PI3K/AKT and mTOR pathways in *Pfn4^−/−^* mice, which was confirmed by western blot. Therefore, LC3I/II and SQSTM1 protein levels were increased, indicating inhibition of autophagy. Finally, we demonstrate that zona-free oocytes treated with *Pfn4^−/−^* sperm develop up to morula stage, suggesting that PFN4 is required for proper acrosome biogenesis and function.

The phenotype observed in PFN4-deficient mice correlates with PFN4 protein subcellular localization in the acrosome and manchette (Behnen et al., 2009). We observed defects in manchette development that seem to result in the amorphous head shape of *Pfn4^−/−^* sperm. Furthermore, we provide evidence that the flagellar structure is affected by the impaired manchette. In addition, acrosome biogenesis is partially aberrant, starting from the Golgi phase in *Pfn4^−/−^* mice. Hence, we show that PFN4 contributes to manchette formation, protein transport, flagellar structural integrity, sperm motility, acrosome biogenesis and sperm head morphology.

*Pfn4^−/−^* mice display greatly reduced sperm viability as a result of severe defects in sperm membrane integrity. The infertility seems to be mainly caused by perturbation of manchette development and acrosome biogenesis.

PFN4 is localized in the manchette of spermatids during elongation steps (Behnen et al., 2009). We observed defects in manchette development in *Pfn4^−/−^* sperm. Several studies on spermiogenesis-related genes, e.g. *Hook1* (Mendoza-Lujambio et al., 2002), *Ift20* (Li et al., 2016), *Pfn3* (Umer et al., 2021), *Spag17* (Kazarian et al., 2018), *Lrguk1* (Okuda et al., 2017), *Spef2* (Lehti et al., 2017), *Gopc* (Yao et al., 2002) and *Cdc42* (Chapin et al., 2001), showed aberrant manchette development resulting in altered sperm head shape. The manchette and nucleus are connected with linkers, suggesting a structural relationship between manchette and nucleus. Furthermore, clutching forces exerted by the manchette are responsible for shaping of the sperm head (Kierszenbaum and Tres, 2004).

In addition, we observed disrupted localization of HOOK1 and ARL3 in the manchette of *Pfn4^−/−^* spermatids. Interestingly HOOK1-deficient males display a similar phenotype (impaired manchette and flagellar development, abnormal sperm head shape, reduced motility and malformed acrosome formation) as we observed in our *Pfn4^−/−^* males (Schwarz et al., 2017). Recently, it has been reported that HOOK1 interacts with CCDC181 (coiled-coil domain containing protein 181) and plays a role in flagellar development and regulation (Schwarz et al., 2017). HOOK1 acts as cargo protein for CCDC181 protein in the IMT process. We observed perinuclear ring staining using HOOK1, suggesting that perinuclear ring formation is not affected in *Pfn4^−/−^* spermatids; however, in later steps (10-13), we did not observe formation of the microtubular manchette in *Pfn4^−/−^* spermatids. ARL3 plays a role in cargo transport for axoneme formation and is essential for ciliogenesis (Alkanderi et al., 2018). We speculate that PFN4 plays a role as a structural and cargo protein, whereby it might participate in binding motor proteins and transporting their cargo to specific locations during sperm development. We further hypothesize that PFN4 might interact directly or in a complex with HOOK1 and ARL3. Unfortunately, owing to a lack of PFN4 antibody, the exact localization of PFN4 protein (at the ultrastructural level) in sperm flagellar is not known; in addition, the co-immunoprecipitation experiments cannot be performed to find the interacting partners of PFN4. The localization of PFN4 to the manchette suggests a role in its organization and/or remodeling. Indeed, we find that loss of PFN4 results in defective manchette development and a smaller sperm head with severely altered shape. PFN3 also localized in the manchette and *Pfn3^−/−^* mice show amorphous sperm head morphology as a result of abnormal manchette development, which is comparable to the phenotype of *Pfn4^−/−^* mice.

As described previously, the manchette also contributes to the cargo of motor proteins before transporting them for tail formation through IMT. We observed flagellar defects that contribute to the reduced sperm motility in PFN4-deficient mice. The phenotypic similarities observed in other knockout mouse models, e.g. LRGUK (Liu et al., 2015), MEIG1 (Li et al., 2015), PACRG (Li et al., 2015), SUN4 (SPAG4) (Calvi et al., 2015), HOOK1 (Mendoza-Lujambio et al., 2002) and GOPC (Yao et al., 2002), emphasize the significance of the IMT mechanism in sperm flagellar development. This underlies the movement of proteins via acrosome-acroplaxome-manchette-tail association for manchette development, sperm head shaping, acrosome biogenesis, maintaining Golgi sub-domains, and flagella formation. However, further studies are needed to elucidate the role of PFN4 in IFT during late steps of development of sperm tail.

Ultrastructural examination of *Pfn4^−/−^* sperm revealed that round spermatids fail to properly fuse proacrosomal granules/vesicles to form the acrosome. Further analysis using *cis-* and *trans-*Golgi markers indicates that the structural disorganization of Golgi sub-domains might be the reason for aberrant proacrosomal vesicle formation and fusion during acrosome biogenesis. IHC using Sp56 and acrosin also showed disturbed acrosome biogenesis in PFN4-deficient mice. These results suggest a role of PFN4 in vesicle trafficking and protein transport from the Golgi network for acrosome biogenesis. Interestingly, PICK1, GM130 and PFN3 proteins have been shown to localize to the Golgi apparatus as well. Mice deficient for these genes also displayed a fragmented Golgi network resulting in impaired acrosome development due to failure of proacrosomal granule fusion, as observed in PFN4 knockouts.

In addition, several proteins, such as COPA, PICK1 and GOPC, play a role in vesicle trafficking and transport. It has been reported that mutations in COPA lead to aberrant protein trafficking (Lepelley et al., 2020). We observed mislocalization of COPA, PICK1 and GOPC in PFN4-deficient mice, suggesting disturbed vesicle trafficking and transport and resulting in arrested acrosome formation. We hypothesize that PFN4 might interact directly or indirectly with COPA, PICK1 and GOPC, which are crucial for protein transport and trafficking, and loss of PFN4 might disturb this complex. However, further experiments need to be done to find the interacting partners of PFN4.

We reported previously (Umer et al., 2021) that PFN3 is located in the Golgi apparatus, acrosome and manchette, and hence is similar to PFN4 in subcellular localization (Behnen et al., 2009). Both *Pfn3^−/−^* and *Pfn4^−/−^* male mice display impaired acrosome biogenesis with disrupted Golgi sub-domains. However, *Pfn4^−/−^* mice show a more dramatic phenotype in terms of sperm viability, acrosome biogenesis and fertility compared with *Pfn3^−/−^* mice. We speculate that this is due to PFN4 affecting the main signaling pathway of autophagy (PI3K/AKT), whereas PFN3 affects only downstream targets (mTOR/AMPK) of autophagy (Umer et al., 2021).

Protein trafficking from the Golgi network is vital throughout the process of sperm differentiation. During acrosome development, proacrosomal vesicles are released from the *trans-*Golgi network and fuse to form the acrosome (Tang et al., 1982). In acrosome biogenesis, components of the autophagy network play a role (Wang et al., 2014). They modulate proacrosomal formation, and support transport and fusion towards the nucleus for acrosome development (Shang et al., 2016). P13K/AKT and mTOR are key regulators of autophagy and an imbalance in these factors leads to inhibition of autophagy (Jia et al., 2022). We demonstrate that protein levels of P13K, p-PI3K, AKT and AKT1 were increased in PFN4-deficient mice, likely leading to the activation of mTOR and p-mTOR and suppression of AMPK, resulting in autophagy inhibition. The increase of LC3BI/II and SQSTM1 protein levels in *Pfn4^−/−^* males suggests that autophagosomes accumulate. Ultimately, this seems to result in a blockage of autophagic flux and impaired acrosome biogenesis in PFN4-deficient mice.

SIRT1 (Liu et al., 2017) and ATG7 (Wang et al., 2014) deficient mice show inhibited autophagy, resulting in LC3BI/II and SQSTM1 accumulation and failure of proacrosomal granule formation and fusion. Our study on *Pfn3*^−/−^ shows a disturbance in the mTOR/AMPK pathway along with the accumulation of autophagosomes (Umer et al., 2021). This suggests that SIRT1 and ATG7 proteins play a role in the downstream cascade, whereas PFN3 protein is essential for regulating the central player (mTOR) of autophagy. However, PFN4 seems to play a role in the upstream PI3K/AKT signaling mechanism of autophagy.

Proteomics analysis of *Pfn4^−/−^* mice indicated moderate deregulation of SPECC1L, ARF2:3 and FKBP1 proteins. They play a role in PI3K signaling, the *trans-*Golgi network and vesicle tethering and transport. SPECC1L is known to be a modulator of PI3K/AKT signaling (Wilson et al., 2016), which is essential for autophagy regulation. *Specc1l* mutants exhibit reduced PI3K/AKT signaling (Wilson et al., 2016). We observed increased protein levels of SPECC1L in *Pfn4^−/−^* mice, which seem to lead to the activation of PI3K/AKT signaling, as confirmed by enrichment and western blot analysis (Fig. 6; Fig. S5D). Increased protein levels of AKT leads to activation of mTOR, which results in the inhibition of autophagy (Hahn-Windgassen et al., 2005). ARF2:3 and FKBP1A localize in the *trans-*Golgi network, play a role in post-Golgi trafficking and act as a sorting station from Golgi to plasma membrane (Manolea et al., 2010). Enrichment analysis further showed that biological processes for protein transport are altered in *Pfn4^−/−^* mice. This suggests that loss of PFN4 might disturb the autophagy mechanism responsible for acrosome biogenesis, Golgi structural organization, and vesicle formation, transport and fusion.

Aberrant acrosome biogenesis is likely responsible for the disrupted acrosome reaction and inability of sperm to fertilize oocytes. Indeed, *Pfn4^−/−^* sperm showed a very low acrosomal exocytosis rate compared with controls, but *Pfn4^−/−^* sperm were still able to fertilize oocytes that had been treated to remove the zona pellucida. Thus, impaired acrosome biogenesis seems to be responsible, at least in part, for the *Pfn4^−/−^* male infertility. In contrast to PFN3-deficient mice (Umer et al., 2021), acrosomal exocytosis was affected more severely in the PFN4-deficient mouse model.

In addition, we did not observe any organizational defects in the actin cytoskeleton in *Pfn4^−/−^* testes. This could be because PFN4 does not retain the actin-binding site, and actin organization and polymerization are affected only when F-actin capping or cross-linking protein junctions are disturbed.

Interestingly, we did not observe male germ cell apoptosis and phagocytosis by Sertoli cells in our PFN4-deficient mice. This suggests that inhibition of autophagy caused by disrupted signaling pathways does not contribute to apoptosis in testicular germ cells of *Pfn4^−/−^* males.

Our results suggest that PFN4 plays a role in manchette development and vesicle trafficking from the Golgi network to developing spermatozoa for acrosome biogenesis. In addition, defects in manchette development were observed, which seems to result in abnormal sperm head morphology. Furthermore, we demonstrated the deregulation of PI3K/AKT and mTOR/AMPK signaling pathways, which results in the autophagy inhibition during acrosome biogenesis in *Pfn4^−/−^* mice. This unusual member of the profilin family is essential for mouse male fertility and is an intriguing target for studies concerning human male factor infertility.

## MATERIALS AND METHODS

### Ethical statement

Animal care, breeding setup and all experimental experiments were approved according to German laws of animal protection and in agreement with the approval of the local institutional animal care committees (Landesamt für Natur, Umwelt und Verbraucherschutz, North Rhine-Westphalia, approval ID: AZ84- 02.04.2013.A429).

### Generation of *Pfn4* knockout mice

Superovulation was achieved by intraperitoneal injection of C57BL/6J female mice with pregnant mare serum (5 IU) and human chorionic gonadotropin (5 IU); two females were then mated with one C57BL/6J male. Zygotes were isolated and injected as described previously (Umer et al., 2021). *Pfn4* knockout mice were generated by co-injection of *Cas9* mRNA (100 ng/μl) (Sigma-Aldrich), and *in vitro-*transcribed single guide RNAs (50 ng/μl each guide) (Table S2) into the cytoplasm of zygotes. The zygotes surviving after microinjection were kept for 3 days in KSOM medium in a CO_2_ incubator. The resulting blastocysts were transferred into the uteri of pseudo-pregnant foster recipients as described previously (Umer et al., 2021).

### Genotyping PCR

Genomic DNA was isolated using phenol:chloroform methodology from mouse tails and subjected to PCR. Gene-specific primers were designed and used for genotyping PCR. PCR products were sequenced to identify the locus-specific mutations mediated by CRISPR/Cas9 genome editing. Primers are listed in Table S1.

### Fertility analysis

For fertility analysis, a total of seven to nine WT and knockout male mice aged 10-12 weeks were individually mated with sexually mature C57BL/6J WT females in a controlled breeding experiment. Females were observed for the vaginal plugs and pregnancies. The average litter size from each pregnant female was calculated.

### Morphological analysis

*Pfn4^−/−^*, *Pfn4^+/−^* and WT littermate male mice were used for phenotypic characterization (body, testes and cauda epididymis weights) as previously reported (Umer et al., 2021) (*n*=9 animals/genotype). Male mice aged 10-12 weeks were used in this study.

### Epididymis sperm assessment

Epididymal sperm were extracted by multiple incisions into the cauda epididymis followed by a swim-out for 30-60 min in M2 medium. Sperm viability analysis was performed using E&N staining and sperm membrane integrity was accessed by the hypo-osmotic swelling test (Umer et al., 2021).

### Nuclear morphology analysis

For nuclear morphology analysis, after swim-out sperm cells were washed three times followed by fixation in 2% paraformaldehyde. Sperm cells were diluted in fixative and spread evenly on a glass slide and allowed to air dry. Slides were counterstained with DAPI (Carl Roth) as described previously (Skinner et al., 2019). Analysis was performed on three independent experiments in total (*n*=3/genotype). Images were taken using a Leica DM5500 B/JVC KY-F75U digital camera. Images were analyzed using the ImageJ plugin ‘Nuclear morphology analysis v1.15.3’ according to the developer's instructions.

### Protein extraction and western blot analysis

For total protein extracts from the testes, tissue was homogenized using a Dounce homogenizer (Sartorius) into 500-900 μl RIPA buffer (Thermo Fisher Scientific). The homogenized tissue was kept for 15 min on ice followed by 15 min centrifugation on 4°C at 13,000 rpm (17,383 ***g***). In order to load equal amounts of protein, the concentration of supernatant was measured using the Pierce BCA Protein-Assay Kit (Thermo Fisher Scientific). SDS gel followed by western blot was performed as described previously (Umer et al., 2021) with primary antibodies against LC3I/II (1:1000; ab58610, Abcam), p-mTOR (1:1000; 2971, Cell Signaling Technology), mTOR (1:1000; 2972, Cell Signaling Technology), SQSTM1 (1:1000; 5114, Cell Signaling Technology), AMPK (1:1000; 5831, Cell Signaling Technology), β-actin (1:10,000; A5441, Sigma-Aldrich), as described previously (Umer et al., 2021), ARF3 (1:1000; 10800-1-AP), FKBP1 (1:1000; 10273-1-AP) (Hou et al., 2017), SPECC1L (1:1000; PA5-71632; Thermo Fisher Scientific), p-AKT (1:2000; 4060, Cell Signaling Technology), p-PI3K (1:1000; 17366, Cell Signaling Technology), PI3K (1:1000; 4263, Cell Signaling Technology) and AKT (1:1000; 4691, Cell Signaling Technology), as described previously (Chen et al., 2022).

### cDNA synthesis and qRT-PCR

After removal of the tunica albuginea from testes tissue, RNA isolation using TRIzol reagent according to manufacturer's protocol (Life Technologies) was performed. The concentration and purity of isolated RNA was measured using NanoDrop (Peqlab). After DNaseI treatment of RNA, cDNA synthesis was performed using the RevertAid First Strand cDNA Synthesis Kit (Fermentas, now Thermo Fisher Scientific). qRT-PCR was performed using Maxima SYBR Green qPCR Master Mix (Life Technologies) on ViiA 7 Real Time PCR System (Applied Biosystems, distributed by Life Technologies) as described previously (Umer et al., 2021). At the end of each PCR run, a melting point analysis was performed. *Gapdh* was used as reference gene for data normalization.

### TEM

Testes tissue and epididymal mature sperm cells were washed in PBS and fixed in 1.5% glutaraldehyde and 0.1 M cacodylate buffer (pH 7.4). Sperm cells were washed with 0.1 M cacodylate buffer, followed by fixation in 2% osmium tetroxide with an additional wash in 0.1 M cacodylate buffer. Further steps were performed as described previously (Umer et al., 2021). Images of ultra-thin sections were taken using a Verios 460L microscope (FEI) with a STEM III-detector.

### Immunohistochemistry

Bouin's fixed 5-μm thick sections were processed for immunohistochemistry. Staining was carried out using a Lab Vision PT module (Thermo Fisher Scientific) and Autostainer 480S (medac) as described previously (Umer et al., 2021). Primary antibodies against HOOK1 (1:200; 10871-1-AP) (Zhou et al., 2009), LC3B (1:100; ab58610, Abcam), ARL3 (1:200; 10961-1-AP) (Qi et al., 2013), GOPC (1:200; 12163-1-AP, Thermo Fisher Scientific), COPA (1:200; BS-12464R, Thermo Fisher Scientific), PICK1 (1:200; PA5-76084, Thermo Fisher Scientific), SP56 (1:200; MA1-10866, Thermo Fisher Scientific), acrosin (1:200; BS-5151R, Thermo Fisher Scientific), AKT1 (1:100; 4060, Cell Signaling Technology), SQSTM1 (1:200; 5114, Cell Signaling Technology) and ARF3 (1:200; 10800-1-AP, Proteintech) were used.

### Isolation of germ cell population from testes

Testes were collected in ice-cold PBS and the tunica albuginea was removed carefully. Testicular tissue was minced in 200 μl of digestion medium [1 mg/ml collagenase/dispase (10269638001, Roche), 1 mg/ml hyaluronidase (H3506; Sigma-Aldrich), 1 mg/ml DNAse I (DN-25; Sigma-Aldrich) in DMEM] until all seminiferous tubules seemed to be digested. Then 800 μl of digestion medium was added and incubated for 25 min at 37°C with slow continuous rotation. Additional mechanical disruption was performed by pipetting the solution five times using recovery tips. The suspension was filtered through a 35 mm pore size filter to achieve a single-cell suspension. The cell suspension was pipetted slowly to avoid clogging and cell damage. Cells were collected by centrifugation at 400 ***g*** for 10 min at 37°C, the supernatant was discarded, and the pellet was resuspended in 1 ml of PBS (Umer et al., 2021).

### Immunofluorescence staining

Immunofluorescence staining was performed on testes sections using PNA-FITC Alexa Fluor 488 conjugate (Molecular Probes, Invitrogen), anti-mouse GM130 (1:250; 610823, BD Biosciences) and anti-rabbit TGN46 (1:100; JF1-024, Thermo Fisher Scientific) to assess acrosome biogenesis, *cis-* and *trans-*Golgi structural organization, respectively. Immunofluorescence staining of α-tubulin to detect the manchette was performed as described (Umer et al., 2021) on testes sections, epididymal sperm cells and the isolated germ cell population. Images were taken within 24 h using an LSM 710 confocal microscope (Zeiss).

### PAS staining

Bouin's fixed 5-μm thick testes sections were deparaffinized and rehydrated followed by incubation for 10 min with periodic acid (0.5% in H_2_O). Slides were washed with H_2_O and incubated for 20 min with Schiff reagent. Finally, slides were rinsed for 7 min with H_2_O, followed by counterstaining with Hematoxylin, dehydration and mounting with a coverslip. Images were taken using a panoramic 3DHistech™ slide scanner.

### Mass spectrometry

To prepare tissue for mass spectrometry, 20 mg of testes tissue was suspended in 200 µl LYSE buffer from the iST-NHS sample preparation kit (PreOmics) supplemented with 1× HALT1/2 protease inhibitors (Thermo Fisher Scientific). To each sample, 50 mg glass beads with 0.5 mm diameter (VWR International) were added. Tissues were disrupted in a Bioruptor ultrasound device (Diagenode SA) at 4°C. Samples were reduced by incubation at 95°C for 10 min shaking in a thermomixer at 1000 rpm. Insoluble matter was separated by centrifugation (10 min, 14,000 ***g***, 10°C) and the supernatant was transferred to a new reaction tube. An aliquot was diluted tenfold in water for measurement of protein content with a BCA assay (Pierce Thermo Scientific) according to the manufacturer's instructions.

Solution containing 50 µg protein was added to 50 µl of DIGEST buffer (iST-NHS kit) for digestion of proteins (3 h, 37°C) and 0.4 mg of TMT10plex isobaric Mass Tag Labeling reagent was added to each sample and incubated at room temperature for 1 h. The reaction was quenched with 10 µl hydroxylamine. The preparation procedure was continued according to the iST-NHS kit instructions. Pooled peptides were dried in a vacuum concentrator, dissolved in IPG buffer pH 3-10 (GE Healthcare) and fractionated with an Off Gel device (Agilent) according to the manufacturer's instructions. Dried peptide fractions were desalted using ZipTip C18 tips (Sigma-Aldrich Chemie). Eluates were dried and re-dissolved in 10 µl 0.1% formic acid (FA).

Peptide separation was performed on a Dionex Ultimate 3000 RSLC nano HPLC system. The autosampler was operated in ‘μl-pickup’ mode. Peptides were dissolved in 10 µl 0.1% FA (solvent A) and 1 µl of this solution was injected onto a C18 analytical column (self-packed 300 mm length, 75 µm inner diameter, ReproSil-Pur 120 C18-AQ, 1.9 µm, Dr. Maisch). Peptides were separated over a linear gradient from 5% to 35% of solvent B (90% acetonitrile, 0.1% FA) at 300 nl/min. The nanoHPLC was coupled online to an Orbitrap Fusion Lumos mass spectrometer (Thermo Fisher Scientific). Gradient length was 120 min. Peptide ions between 330 and 1600 m/z were scanned in the Orbitrap detector every 3 s with a resolution of 120,000 (maximum fill time 50 ms, AGC target 100%). Polysiloxane (445.12002 Da) was used for internal calibration (typical mass error ≤1.5 ppm). Using a top-speed method, peptides were subjected to collision-induced dissociation for identification (CID: 0.7 Da isolation, normalized energy 35%) and fragments analyzed in the linear ion trap with target 10,000 and maximum fill time 35 ms, turbo mode. Fragmented peptide ions were excluded from repeat analysis for 25 s. The top eight fragment ions were chosen for synchronous precursor selection and fragmented with higher energy CID (HCD: 2 Da MS2 isolation, 65% collision energy) for detection of reporter ions in the Orbitrap analyzer (resolution 50,000, maximum fill time 86 ms, target 100,000).

Raw data processing and database search were performed using Proteome Discoverer software 2.5.0.400 (Thermo Fisher Scientific). Peptide identification was carried out with an in-house Mascot server version 2.6.1 (Matrix Science). MS data were searched against *Mus musculus* sequences from the SwissProt database including isoforms (2019/06, 560,459+40,403 sequences) and contaminants database (cRAP) (Mellacheruvu et al., 2013). Precursor ion m/z tolerance was 10 ppm, fragment ion tolerance 0.5 Da (CID). Tryptic peptides with up to two missed cleavages were searched. C_6_H_11_NO-modification of cysteines (delta mass of 113.08406) and tandem mass tag (TMT) on N-termini and lysines were set as static modifications. Oxidation was allowed as dynamic modification of methionine. Mascot results were evaluated using the Percolator algorithm version 3.02.1 (The et al., 2016) as implemented in Proteome Discoverer. Spectra with identifications above 1% q-value were sent to a second round of database search with semi tryptic enzyme specificity (one missed cleavage allowed). Protein N-terminal acetylation, methionine oxidation, TMT and cysteine alkylation were then set as dynamic modifications. Actual false discovery rate values were 0.6% (peptide spectrum matches) and 1.0% (peptides and proteins). Reporter ion intensities (most confident centroid) were extracted from the MS3 level, with co-isolation <90% and SPS mass match >65%.

The statistical analyses of the MS data was performed using an in-house developed workflow in R environment (R version 3.6) (https://www.gbif.org/tool/81287/r-a-language-and-environment-for-statistical-computing). Prior to the statistical analysis, non-unique peptides and single-shot proteins were removed. From all available fractions, only those with a minimum number of missing values per peptide-spectrum match (PSM) and across all TMT channels were selected. When this filter returned more than one fraction per PSM, the one with the highest average intensity across all TMT channels was selected. The PSM-level data were then transformed and variance-stabilized using the VSN package (Huber et al., 2002) and then aggregated from PSM-level to protein-level intensities using Tukey's median polish method. The statistical inference analysis was performed using the R package Limma (Ritchie et al., 2015), in which the peptide pools were modeled as fixed effects. The resulting *P*-values for each statistical contrast were adjusted for multiple testing using the Benjamini–Hochberg method and the false discovery rates were calculated. The volcano plots, heatmaps and PCA plots were generated using ggplot2, ComplexHeatmap (Gu et al., 2016) and FactoMineR (Lê et al., 2008) packages, respectively.

### Acrosomal reaction

After sperm swim-out in M2 medium, capacitation was induced by incubating in HTF medium (Millipore) for 90 min at 37°C, 5% CO_2_. To induce the acrosome reaction, the calcium ionophore A23187 (10 µM; c7522, Sigma-Aldrich) was added and the experiment was performed according to Umer et al. (2021). Spermatozoa for each genotype (*n*=3/genotype) were assessed by bright-field microscope.

### Statistics

Statistical analysis was performed using Student's *t*-test (two-tailed unpaired and one-tailed paired), and pairwise comparisons between populations using Mann–Whitney *U*-test and ANOVA (Tukey's post-hoc) to ascertain the significance of the variability in the data. The mean of all values for a particular data set is represented in the graphical form. Error bars are used to denote standard deviation. *P*<0.05 was considered statistically significant.

### *In vitro* fertilization

Oocytes from B6D2F1 females were super-ovulated by intraperitoneal injection of 5 IU pregnant mare serum and human chorionic gonadotropin. Oocytes were extracted from the oviducts after 15 h of the last hormone injection and freed from cumulus cells by treatment with 0.5-1 ml hyaluronidase for 30 s to 1 min at 37°C and 5% CO_2_. Oocytes and sperm were co-cultured in M2 medium (Sigma-Aldrich) for 2-4 h. Surviving oocytes were cultivated in KSOM in micro-drops (Gynemed) under mineral oil (Gynemed) at 37°C and 5% CO_2_.

## Supplementary Material

Supplementary information

Reviewer comments
