# Supplementary material for: PFN4 is required for manchette development and acrosome biogenesis during mouse spermiogenesis
Source: Development. 2022 Aug 22;149(16):dev200499. doi: 10.1242/dev.200499 (PMC9481974; doi:10.1242/dev.200499)
Supplement: Supplementary information [file develop-149-200499-s1.pdf]

## Supplementary Figure. 1

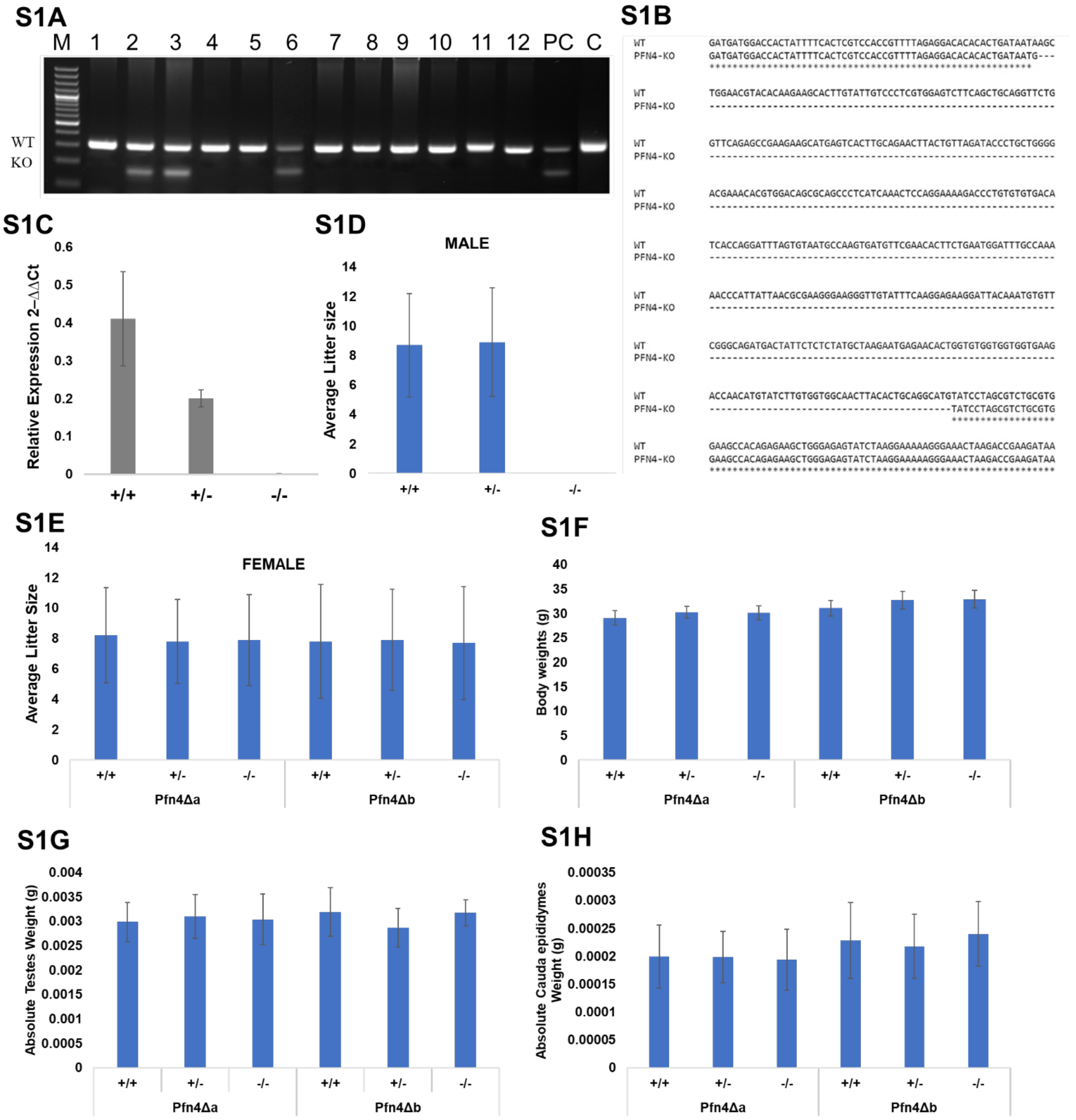


Development • Supplementary information

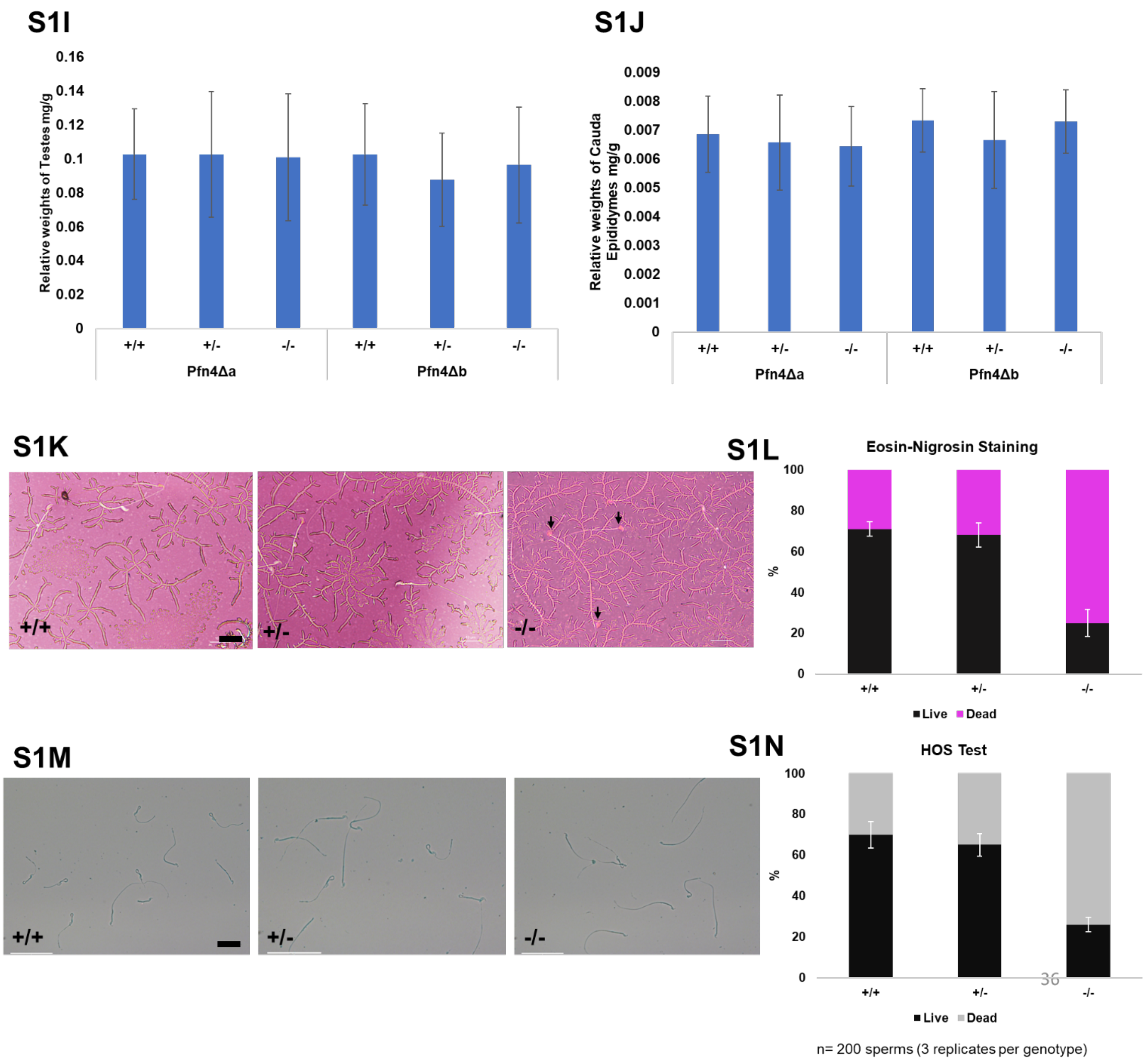


**Fig. S1.** (A) Genotyping for PFN4-deficient founder mice (P#2, 3, and 6 are positive for the deletion). M= 100bp Plus ladder, 302 bp PCR product represents WT allele, and 180bp PCR products represent mutant allele. (B) Sequencing results using clustalW of Pfn4Δ5417 mouse line. Sequences of WT and PFN4-deficient line. * indicates same base pairs and Dash line (-) represents deletion. (C) Validation of PFN4-deficient mice (*Pfn4*Δb). qRT-PCR showed the expression of *Pfn4* mRNA in murine testis of WT, $Pfn4^{+/-}$ and $Pfn4^{-/-}$ mice for



Development • Supplementary information

*Pfn4*Δ5417b mutation. (D) Average litter size of WT, *Pfn4*$^{+/-}$ and *Pfn4*$^{-/-}$ male for *Pfn4*Δb line. (E) Average litter size of WT, *Pfn4*$^{+/-}$ and *Pfn4*$^{-/-}$ female for *Pfn4*Δa and *Pfn4*Δb lines. (F) Body weights in gram, (G, H) Absolute weight of testes and cauda epididymis, (I, J) Relative weight of testes and cauda epididymis of WT, *Pfn4*$^{+/-}$ and *Pfn4*$^{-/-}$ for *Pfn4*Δa and *Pfn4*Δb lines (n=9). (K) Eosin and nigrosine staining of mature WT, *Pfn4*$^{+/-}$ and *Pfn4*$^{-/-}$ sperms isolated from cauda epididymis. Live= white sperm cells, pink=dead sperm cells. Scale bar = 10 µm. (L) Eosin and nigrosine staining on biological replicates (n=3) per genotype of WT, *Pfn4*$^{+/-}$ and *Pfn4*$^{-/-}$ sperms. (M) Hypo-osmotic swelling test performed of mature WT, *Pfn4*$^{+/-}$ and *Pfn4*$^{-/-}$ sperms isolated from cauda epididymis. Tail curling= live sperm cells. Scale bar= 10µm. (N) Hypo-osmotic swelling test on biological replicates (n=3) per genotype of WT, *Pfn4*$^{+/-}$ and *Pfn4*$^{-/-}$ sperm. Scale bar = 10µm. At least 200 spermatozoa were evaluated per sample.

Development • Supplementary information

**Supplementary Figure. 2**

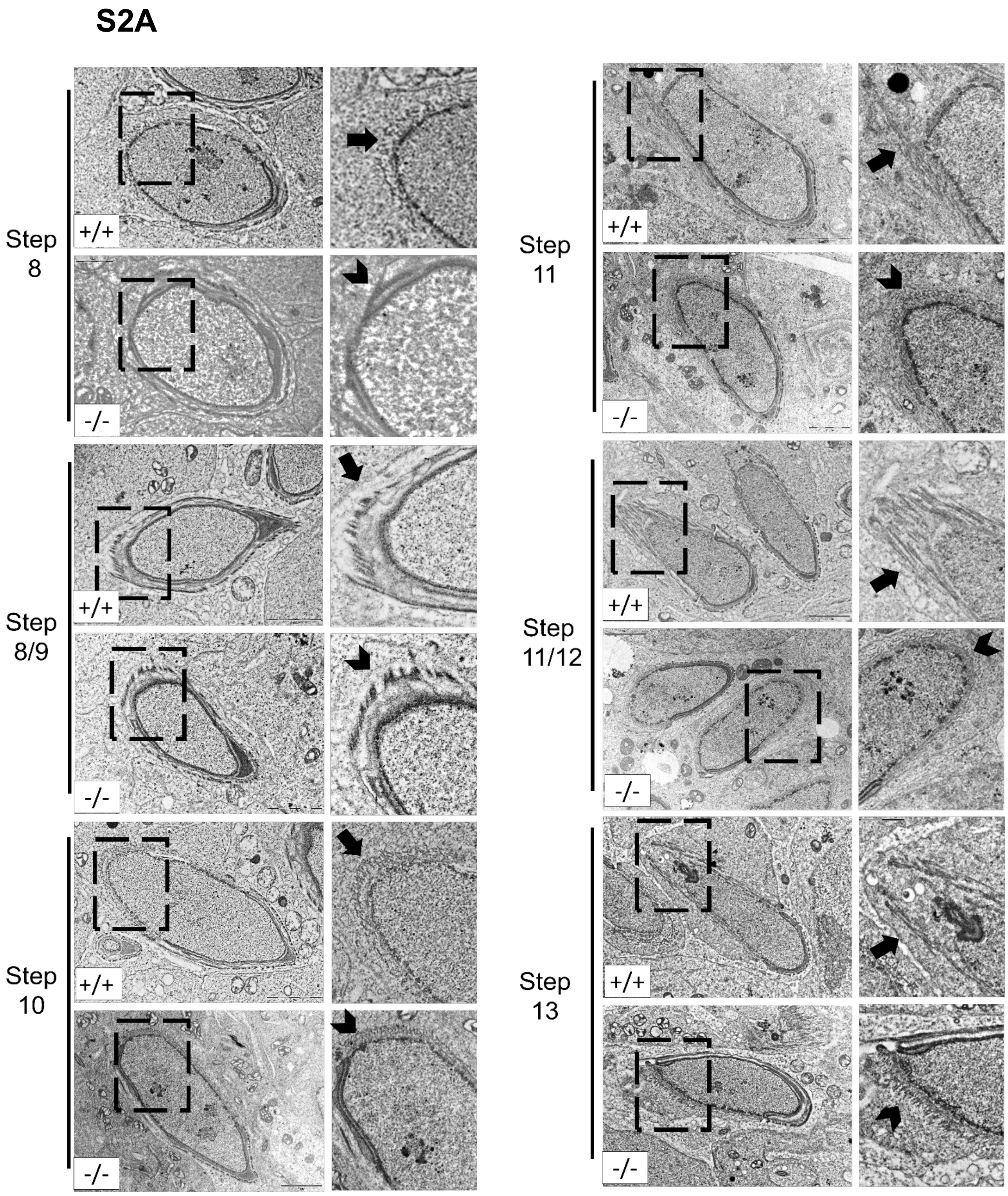


Development • Supplementary information

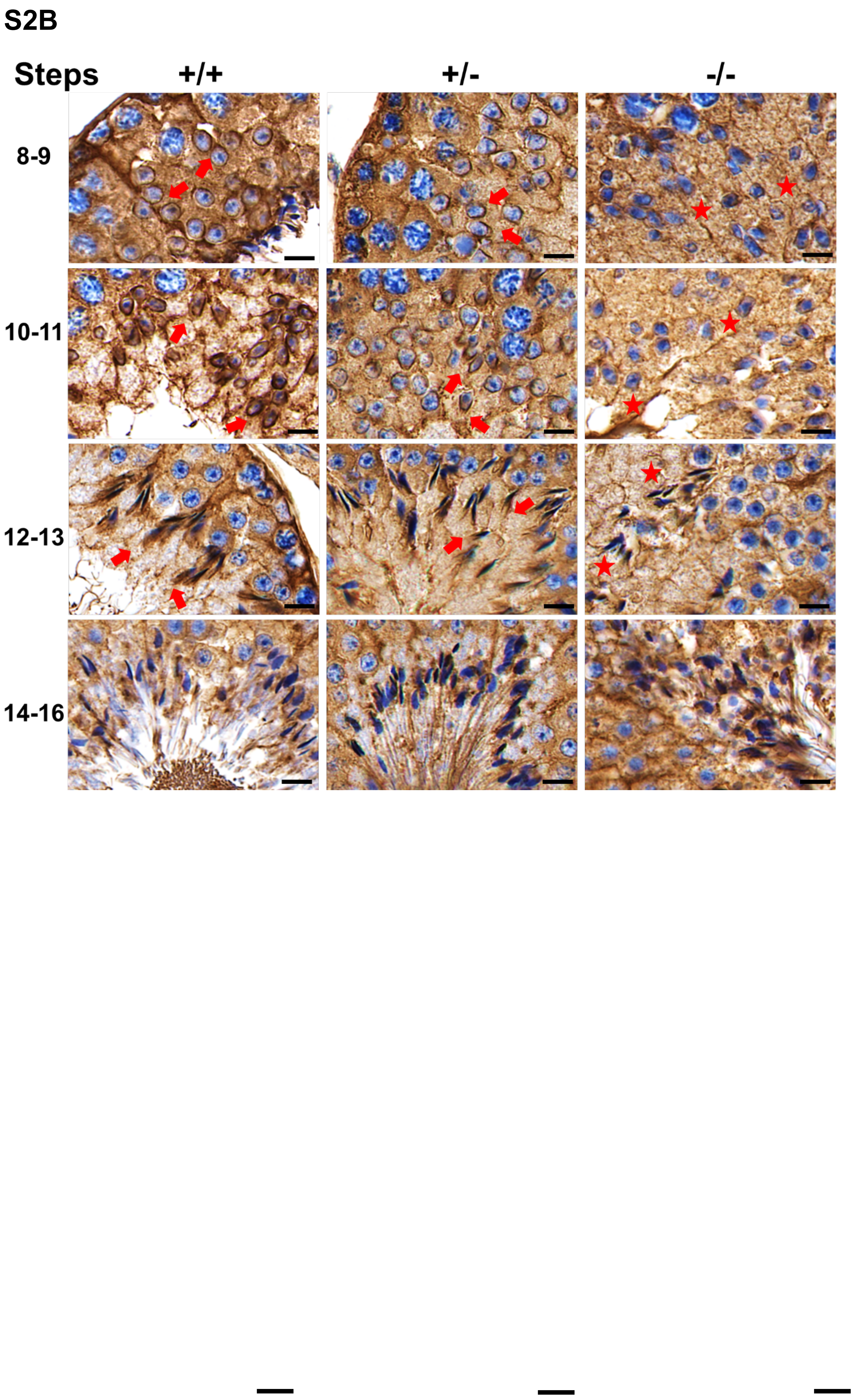


Development • Supplementary information

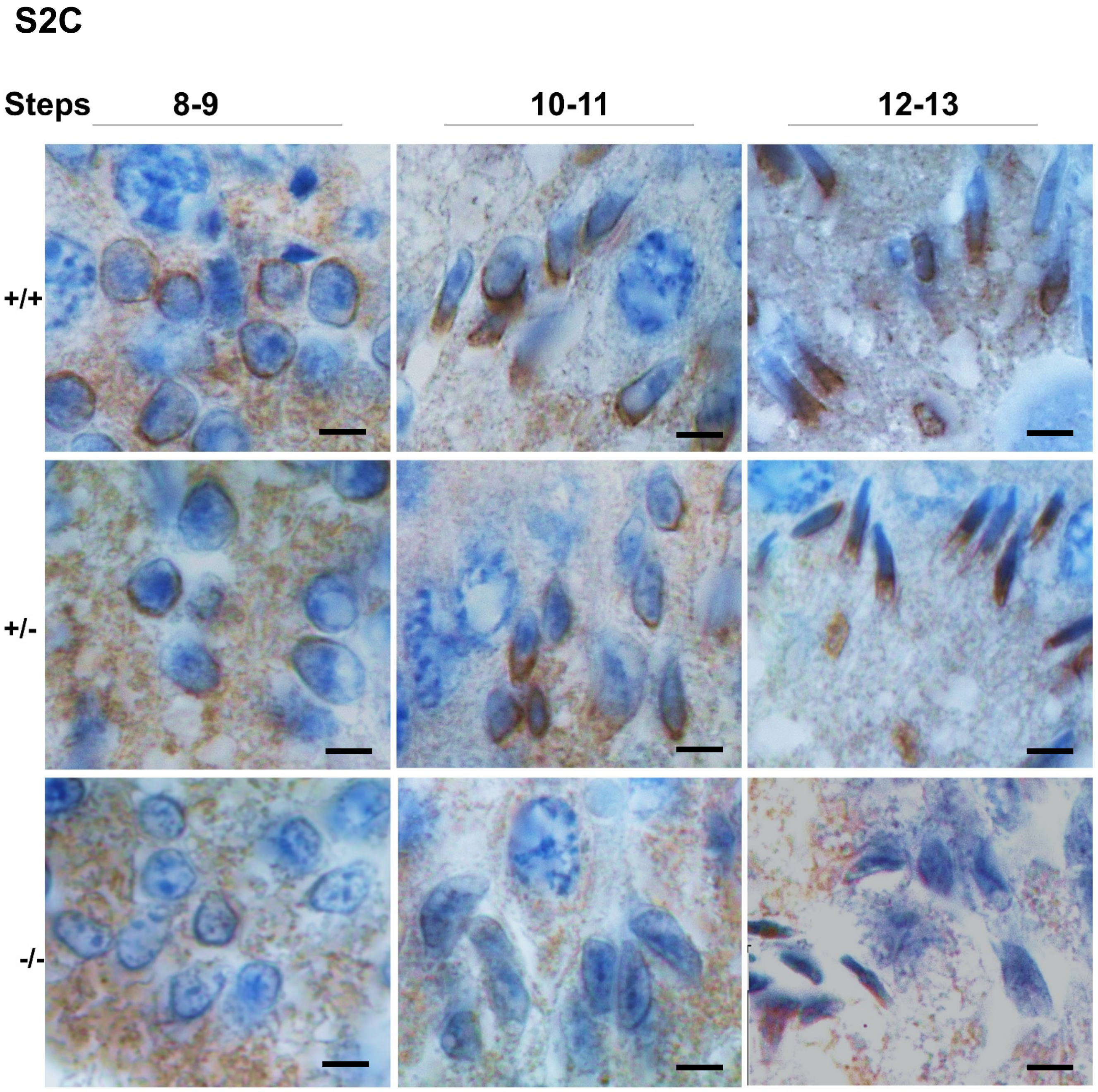


**Fig. S2.** (A) Ultrastructure of manchette formation in developing spermatids from step 8-13 using TEM in WT and *Pfn4*$^{-/-}$ mice. Black arrows indicating manchette formation in WT and arrow heads indicating malformed manchette. Scale bar= 2µm. (B) alpha tubulin staining on WT, *Pfn4*$^{+/-}$ and *Pfn4*$^{-/-}$ testes sections. Scale bar= 20µm. (C) IHC using anti-ARL3 antibody on WT, *Pfn4*$^{+/-}$ and *Pfn4*$^{-/-}$ testes sections. Scale bar= 20µm

Development • Supplementary information

**Supplementary Figure. 3**

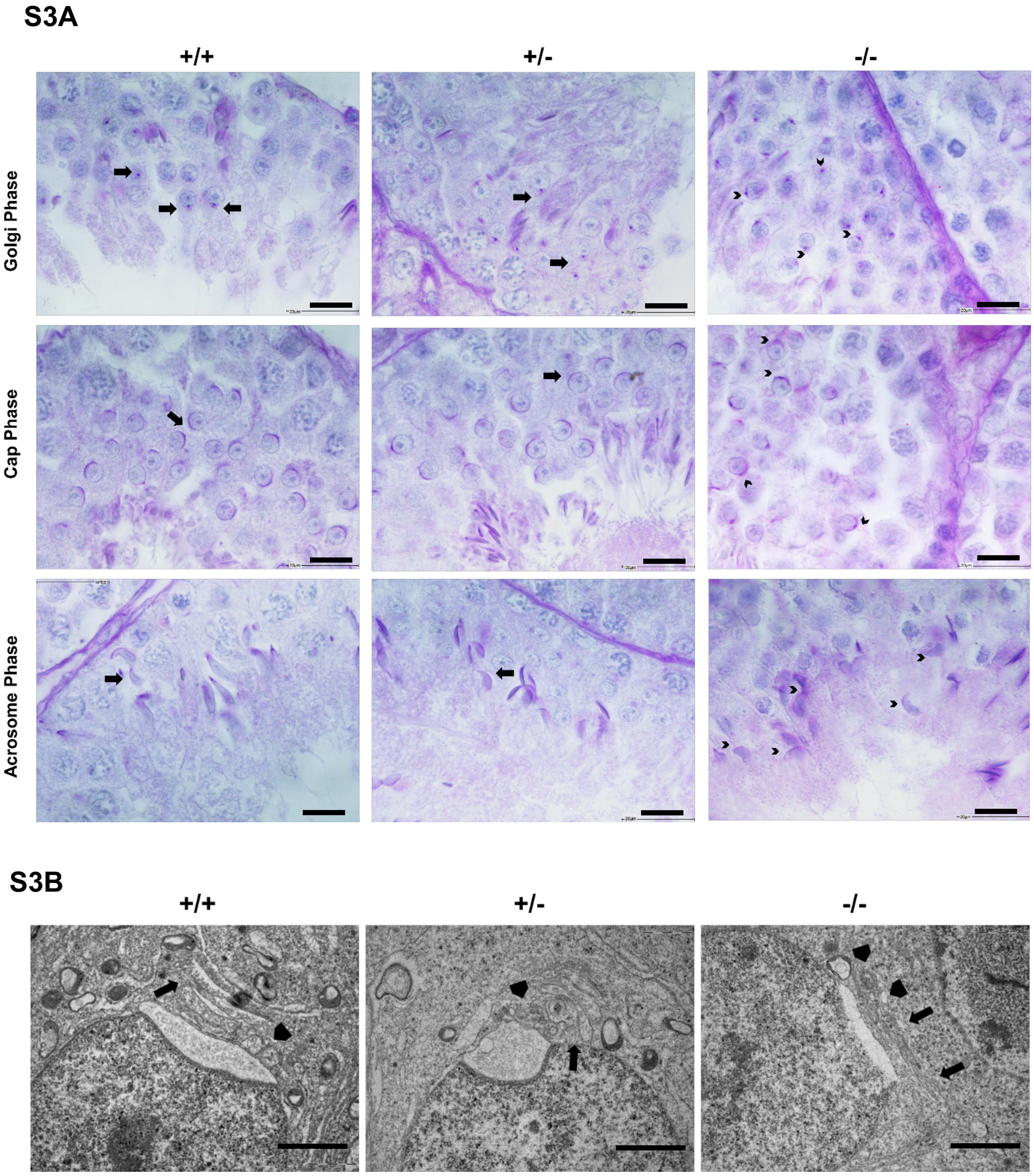

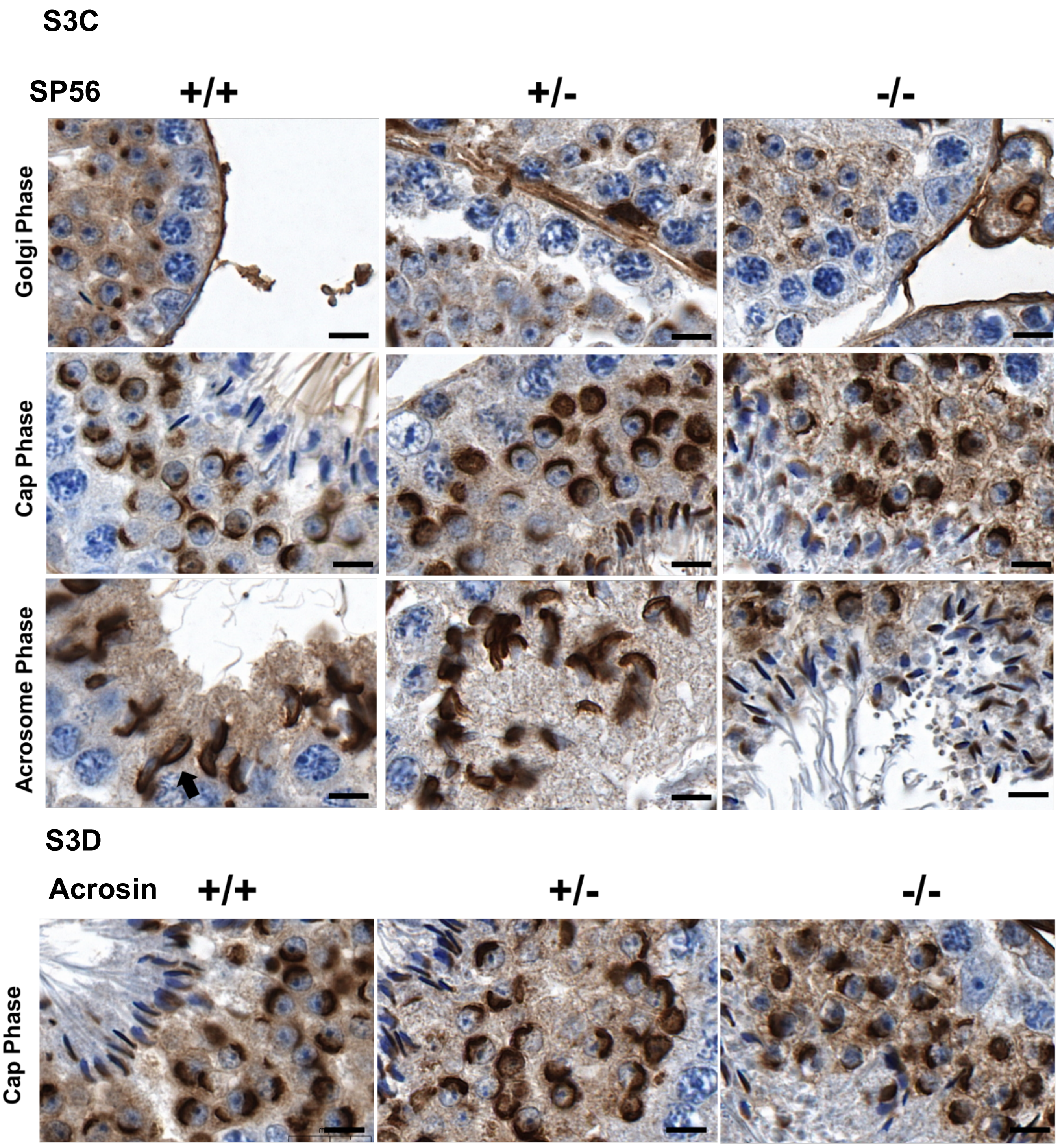


**Fig. S3.** (A) PAS Staining on testes sections of WT, *Pfn4*$^{+/-}$ and *Pfn4*$^{-/-}$ mice. Black arrows indicating normal process of acrosome biogenesis and arrow heads indicating impaired acrosome biogenesis. Scale bar= 20µm. (B) TEM on developing spermatids, *trans-Golgi (arrows) released pro-acrosomal granules (arrowhead) and gather near the acroplaxome* membrane in WT and *Pfn4*$^{+/-}$ testes sections, *Pfn4*$^{-/-}$ section showed dispersed *trans*-Golgi, improper gathering, and formation of proacrosomal granules (arrow heads) Scale bar= 1µm. (C) IHC staining using anti-Sp56 antibody on testes sections of WT, *Pfn4*$^{+/-}$ and *Pfn4*$^{-/-}$ mice for Golgi, Cap and Acrosomal Phase. Scale bar= 20µm. (D) IHC staining using anti-acrosin antibody on testes sections of WT, *Pfn4*$^{+/-}$ and *Pfn4*$^{-/-}$ mice. Scale bar= 20µm.

Development • Supplementary information

**Supplementary Figure. 4**

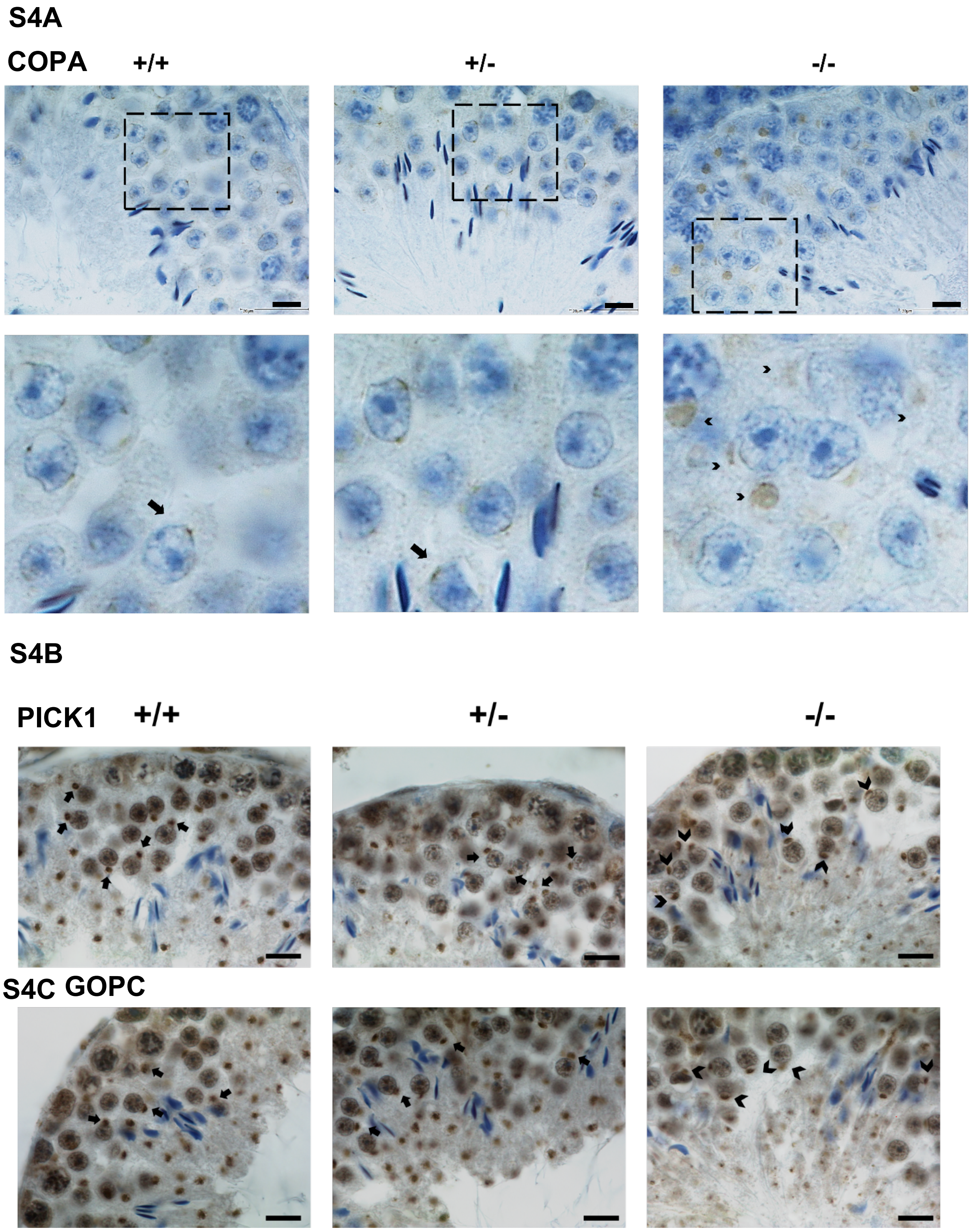


**Fig. S4.** IHC using (A) anti-COPA, (B) anti-PICK1, and (C) anti-GOPC antibodies on WT, *Pfn4*$^{+/-}$ and *Pfn4*$^{-/-}$ testes sections. arrows indicate fine localization of Golgi; arrow heads indicate big Golgi clumps in (A) and miss localization in (B, C). Scale bar= 20µm.

**Supplementary Figure. 5**

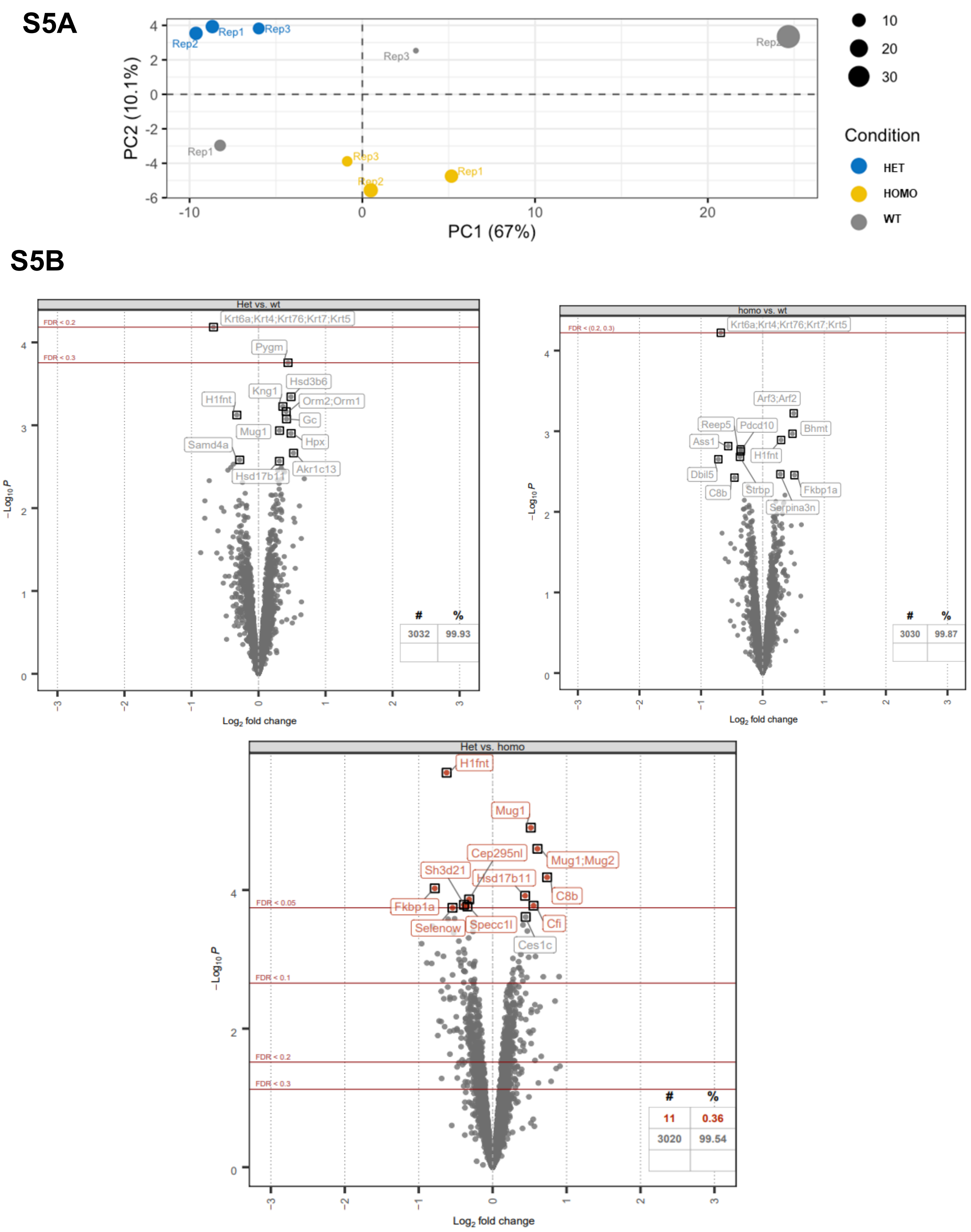


Development • Supplementary information

**S5C**

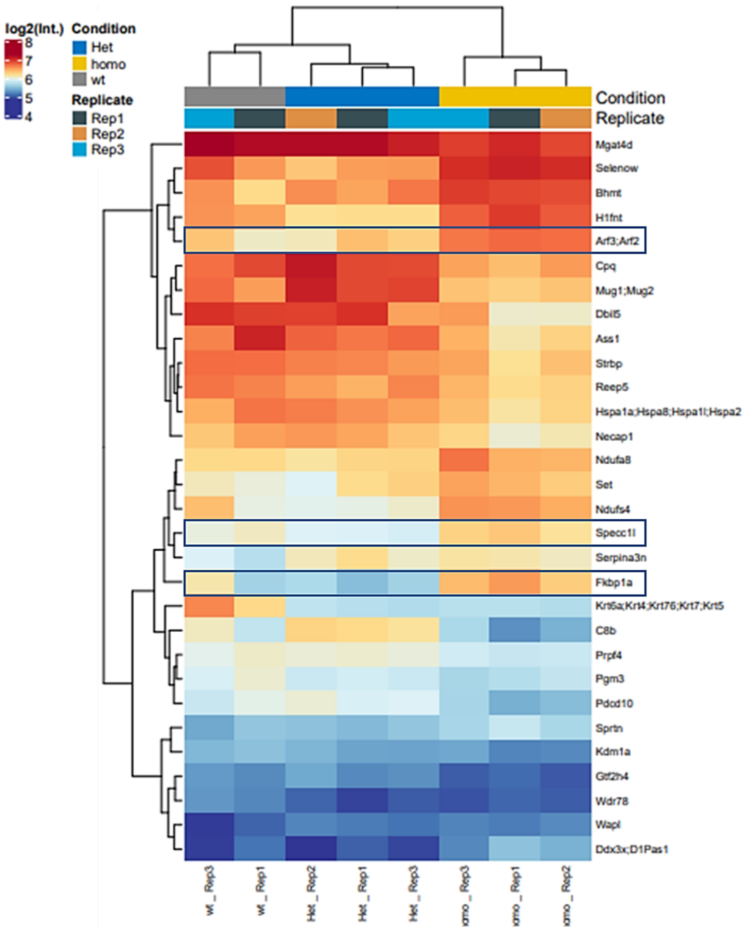


**S5D**

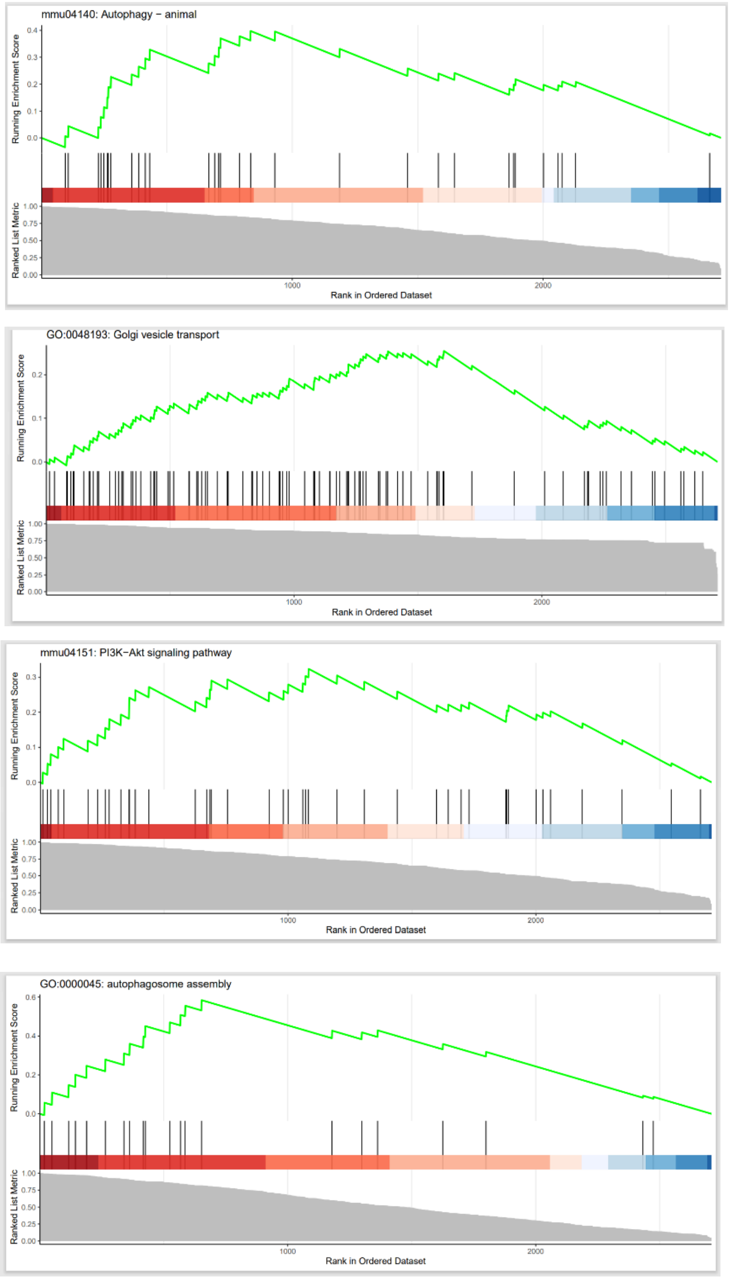


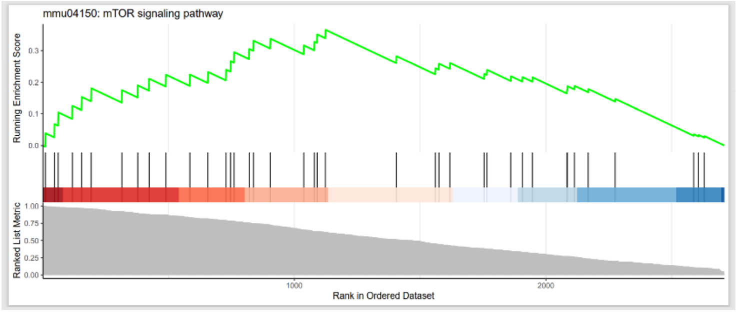


Development • Supplementary information

S5E

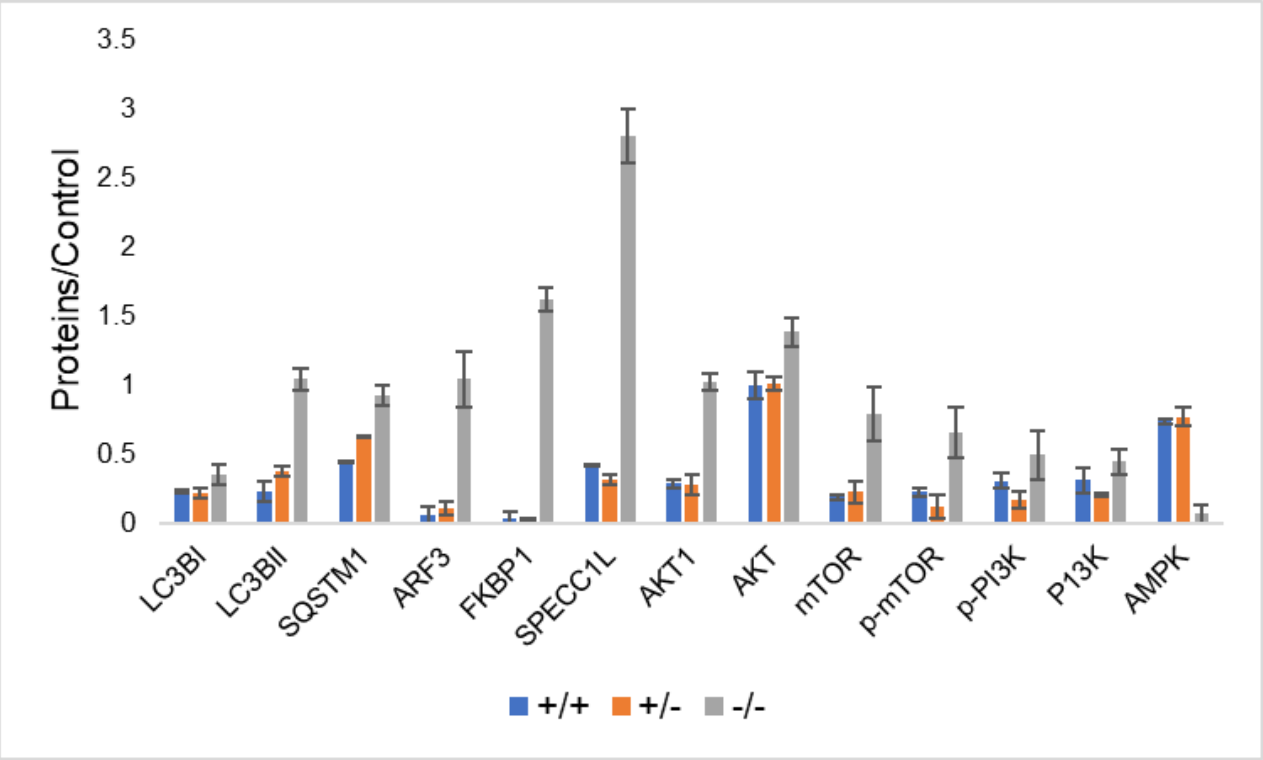


**Fig. S5.** (A) Principal component analysis on mass spectrometry data of WT, *Pfn4*$^{+/-}$ and *Pfn4*$^{-/-}$ samples. Contribution of each data set in PCA represented by the dot size. (B) Volcano plots for WT and *Pfn4*$^{+/-}$ , WT and *Pfn4*$^{-/-}$ , *Pfn4*$^{+/-}$ and *Pfn4*$^{-/-}$ contrasts. (C) Heatmap visualization of 30 deregulated proteins between WT, *Pfn4*$^{+/-}$ and *Pfn4*$^{-/-}$ samples. (D) Enrichment pathway analysis of proteins on WT and *Pfn4*$^{-/-}$ samples. (E) Protein quantification of WT, *Pfn4*$^{+/-}$ and *Pfn4*$^{-/-}$. LC3BI/II, SQSTM1, ARF3, FKBP1, SPECC1L, AKT1, AKT, MTOR, P-MTOR, P-PI3K, PI3K protein levels are increased and similarly AMPK protein level are reduced in *Pfn4*$^{-/-}$ mice. Details are given in main result part.

Development • Supplementary information

**Supplementary Figure. 6**

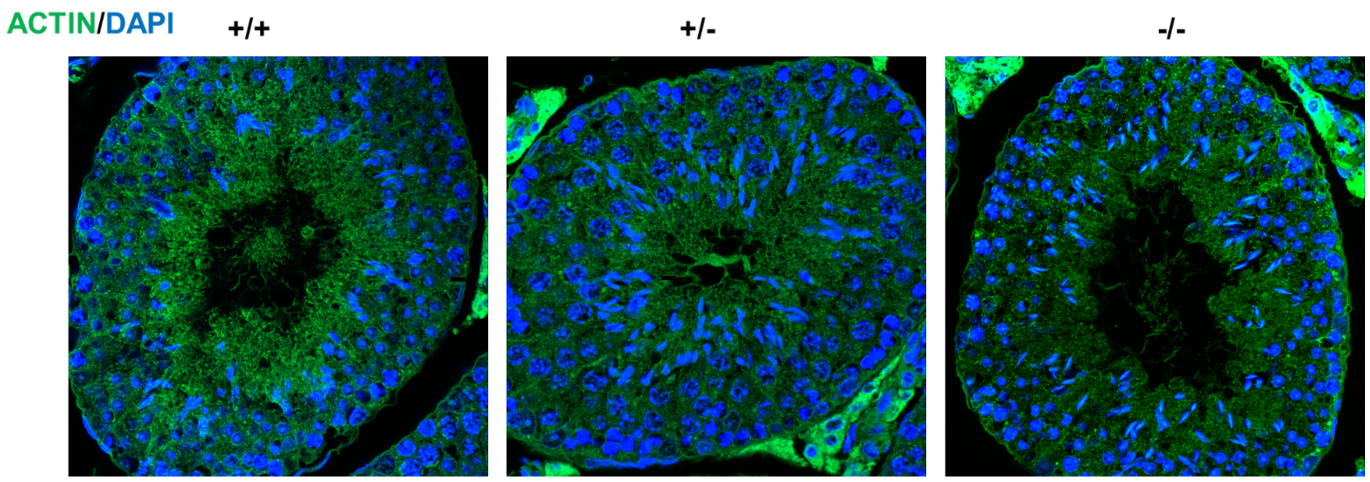


**Fig. S6.** Phalloidin Staining on testes sections of WT, *Pfn4*$^{+/-}$ and *Pfn4*$^{-/-}$ mice. Scale bar= 20µm.

Development • Supplementary information

**Supplementary Figure. 7**

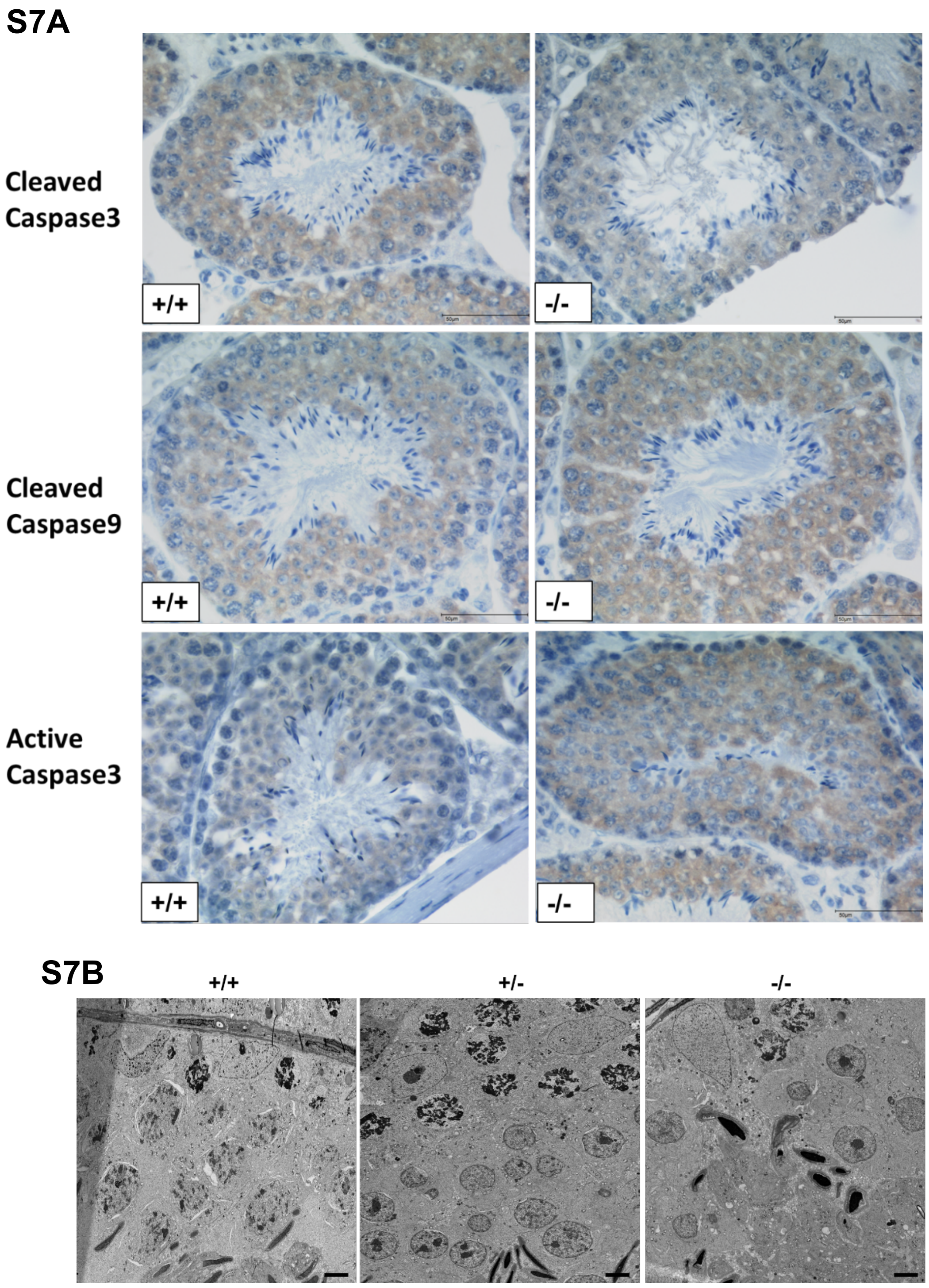


**Fig. S7.** Apoptosis and phagocytosis analysis. (A) IHC of Caspase-3, -9 and active caspase-3 on testes sections of WT, *Pfn4*$^{+/-}$ and *Pfn4*$^{-/-}$ mice. Scale bar= 20µm. (B) Ultrastructure analysis using TEM on testes section of WT, *Pfn4*$^{+/-}$ and *Pfn4*$^{-/-}$ mice. Scale bar= 10µm.

Development • Supplementary information

**Table S1.** Parameters details used in nuclear morphology analysis.

| | WT | Het | Homo Cluster 1 | Homo Cluster 2 |
|---|---|---|---|---|
| **Area mean 95% CI** | 20.13 ± 1.66 | 16.83 ± 1.44 | 15.03 ± 1.36 | 23.31 ± 0.70 |
| **Perimeter mean** | 21.34 | 19.58 | 21.41 | 27.9 |
| **Perimeter mean 95% CI** | 21.34 ± 0.92 | 19.58 ± 0.92 | 21.41 ± 1.31 | 27.90 ± 0.65 |
| **Circularity mean** | 0.53 | 0.53 | 0.43 | 0.4 |
| **Circularity mean 95% CI** | 0.53 ± 0.01 | 0.53 ± 0.01 | 0.43 ± 0.02 | 0.40 ± 0.01 |
| **Regularity mean** | 1.46 | 1.46 | 1.51 | 1.42 |
| **Regularity mean 95% CI** | 1.46 ± 0.02 | 1.46 ± 0.02 | 1.51 ± 0.03 | 1.42 ± 0.02 |
| **Bounding width mean** | 4.92 | 4.54 | 6.14 | 6.36 |
| **Bounding width mean 95% CI** | 4.92 ± 0.24 | 4.54 ± 0.23 | 6.14 ± 0.29 | 6.36 ± 0.12 |

Development • Supplementary information

**Table S2.** gRNAs and primers used in this study.

| gRNA Sequences | Top Strand oligo | Bottom strand oligo |
|---|---|---|
| **Pfn4-gRNA1** | CACCGGACACACACTGATAATAAGC | AAACGCTTATTATCAGTGTGTGTCC |
| **Pfn4-gRNA2** | CACCGGTGGTGGCAACTTACACTGC | AAACGCAGTGTAAGTTGCCACCACC |
| **In-Vitro transcribed Oligos** | **Forward primer** | **Reverse primer** |
| **T7-Pfn4-gRNA1** | TTAATACGACTCACTATAGGGACACACACTGATAATAAGC | |
| **T7-Pfn4-gRNA1** | TTAATACGACTCACTATAGGGTGGTGGCAACTTACACTGC | |
| **T7-sgRNA** | | AAAAGCACCGACTCGGTGCCGCTGAGCCCAGAGCGCGTAG |
| **Genotyping PCR Primers** | | |
| **Pfn4exon2F** | CTTCCCCTCTGAGTACTGGTGAACCCT | **Pfn4exon4R** TTTGGATCCCCCAGGAAGTTCTTCCCT |
| **Pfn41exon2R** | TCACCAGGATTTAGTGTAAGGGTGAAAGC | |
| **qrt-PCR primers** | | |
| **Pfn4-qrtPCR** | CTCCAGGAAAAGACCCTGTG | AATACAACCCTTCCCTTCGC |
| **beta-Actin** | TGTTACCAACTGGGACGACA | GGGGTGTTGAAGGTCTCAAA |

Development • Supplementary information
