# Supplementary material for: PFN4 is required for manchette development and acrosome biogenesis during mouse spermiogenesis
Source: Development. 2022 Aug 22;149(16):dev200499. doi: 10.1242/dev.200499 (PMC9481974; doi:10.1242/dev.200499)
Supplement: Reviewer comments [file develop-149-200499_review_history.pdf]

PFN4 is required for manchette development and acrosome biogenesis during mouse spermiogenesis

Naila Umer, Sharang Phadke, Farhad Shakeri, Lena Arévalo, Keerthika Lohanadan, Gregor Kirfel, Marc Sylvester, Andreas Buness and Hubert Schorle
DOI: 10.1242/dev.200499

Editor: Haruhiko Koseki

Review timeline
Original submission: 4 January 2022
Editorial decision: 14 February 2022
First revision received: 3 June 2022
Editorial decision: 7 July 2022
Second revision received: 13 July 2022
Accepted: 14 July 2022

Original submission

First decision letter

MS ID#: DEVELOP/2022/200499

MS TITLE: Loss of PFN4 leads to male infertility by impairing sperm acrosome biogenesis caused by deregulation of autophagy through the PI3K/AKT/mTOR pathway and defective manchette development.

AUTHORS: Naila Umer, Sharang Phadke, Farhad Shakeri, Lena Arévalo, Keerthika Lohanadan, Gregor Kirfel, Marc Sylvester, Andreas Buness, and Hubert Schorle

I have now received all the referees' reports on the above manuscript, and have reached a decision. The referees' comments are appended below, or you can access them online: please go to BenchPress and click on the 'Manuscripts with Decisions' queue in the Author Area.

As you will see, the referees express considerable interest in your work, but have some significant criticisms and recommend a substantial revision of your manuscript before we can consider publication. If you are able to revise the manuscript along the lines suggested, which may involve further experiments, I will be happy receive a revised version of the manuscript. Your revised paper will be re-reviewed by one or more of the original referees, and acceptance of your manuscript will depend on your addressing satisfactorily the reviewers' major concerns. Please also note that Development will normally permit only one round of major revision.

We are aware that you may be experiencing disruption to the normal running of your lab that make experimental revisions challenging. If it would be helpful, we encourage you to contact us to discuss your revision in greater detail. Please send us a point-by-point response indicating where you are able to address concerns raised (either experimentally or by changes to the text) and where you will not be able to do so within the normal timeframe of a revision. We will then provide further guidance. Please also note that we are happy to extend revision timeframes as necessary.

Please attend to all of the reviewers' comments and ensure that you clearly highlight all changes made in the revised manuscript. Please avoid using 'Tracked changes' in Word files as these are lost in PDF conversion. I should be grateful if you would also provide a point-by-point response detailing

© 2022. Published by The Company of Biologists under the terms of the Creative Commons Attribution License (https://creativecommons.org/licenses/by/4.0/).

how you have dealt with the points raised by the reviewers in the 'Response to Reviewers' box. If you do not agree with any of their criticisms or suggestions please explain clearly why this is so.

Reviewer 1

Advance summary and potential significance to field

Umer et al have characterised the role of profilin 4 (PFN4) in spermatogenesis and male fertility using a Pfn4 knockout (Pfn4-/-) mouse. Profilins are actin binding proteins that have roles in regulating actin dynamics, and a range of cellular processes including membrane trafficking, cytokinesis and cell motility. Here, the authors reveal that PFN4 is essential for male fertility in the mouse and show that a loss of PFN4 results in head shaping and acrosome abnormalities. Towards understanding the mechanism for the acrosome abnormalities, they show that there is dysregulation of key components of the autophagy and Golgi transport pathways.

Overall the study clearly shows an important role for PFN4 in male fertility, and will be of interest to researchers in the male reproductive biology field.
However, characterisation of the specific aspects of the acrosome and manchette phenotypes need to be strengthened and go into much more depth to ensure the data and interpretations are convincing.

Comments for the author

Specific comments:

1. The basic characterisation of the male fertility phenotype in Fig 1 needs to be improved:
a. Testis and body weight are independent of body size and thus should not be expressed as a ration of body weight.
b. The histological characterisation of the testis needs to re-done. Testes should be stained with Periodic Acid Schiff’s stain and hematoxylin (not hematoxylin and eosin) to allow proper stage specific characterisation of the seminiferous epithelium (this will also provide good insight on acrosome formation as PAS stains the acrosome and proacrosomal vesicles so when there is a defect in vesicle trafficking it is quite apparent). Higher magnification images should also be used to point out the abnormal shaped sperm heads, they are quite hard to discern in the current Fig 1G images.
c. Sperm motility should be assessed.

2. The authors show that sperm head shaping is disrupted in the Pfn4-/- mice.
Towards understanding the origin of this phenotype they stain isolated germ cells for alpha tubulin to look at manchette development and dynamics. From the data presented it appears that no manchettes from in the Pfn4-/- mice which is a really interesting finding however this data needs to be strengthened for it be convincing:
a. The finding should be confirmed by electron microscopy to look at MT cytoskeleton ultrastructure and/or IHC staining of testis sections for alpha tubulin so that the spermatids can be properly staged. Indeed, given that head-shaping is abnormal this will impact accuracy of staging of isolated germ cells in the Pfn4-/-.
b. Also need to more precisely define in text how the manchette is disrupted.
3. The authors show that Pfn4-/- mice have a high percentage of dead epididymal sperm, in addition they show that a high percentage of Pfn4-/- epididymal sperm have an amorphous and smaller head shape. Is it possible that these two things are related i.e. that the small amorphous shape is just due to the dead sperm becoming pyknotic as they degrade? Is it possible to characterise the head shapes of just the viable sperm to exclude the possibility that that the defect seen is just due to cell death?
4. In Fig 4 the authors report that acrosome biogenesis is disrupted.
However, while the EM data is clear, in the IF data panels A-I is not convincing for showing a defect in acrosome biogenesis. It is unclear what is meant by non-uniform acrosome vesicle for the in text description of Fig 4C – non uniform in shape, or thickness or gaps in it? Overall, the KOs look normal particularly in 4C and in panels F it appears the spermatids are at an earlier stage than panels D

© 2022. Published by The Company of Biologists under the terms of the Creative Commons Attribution License (https://creativecommons.org/licenses/by/4.0/).

and E. Similarly, it is hard to tell if Panel I is just an earlier stage than G and H or if is abnormal. Assessing PAS-stained sections may make the data more convincing and will help make seminiferous tubule stage more clear.
5. In Fig 4 the EM pictures are nice however, even in the source file they are quite pixelated please fix for the final submission – in particular it would be good to be able to discern the detail of the acroplaxome and marginal ring.
Also, the Fig 4 EM panels needs to be clarified. In particular the authors state that for Fig 4m ‘ in Pfn4-/- mice, the proacrosomal vesicles released from trans-Golgi network do not seem to gather and fuse on the apical face of developing spermatozoa, suggesting that the acrosomal vesicle did not form properly’. However in subsequent stages they show that acrosome does form albeit abnormally. Would this not suggest that this process is just delayed as opposed to failing completely?
6. Fig 5 – Please clarify how the cis-Golgi staining shows that it is a non-polarised stack in Pfn4-/-.
7. Fig 8 – needs more clarification in the figure legend. Is 1-cell effectively ones that haven’t fertilised?
8. In the discussion the authors state that the PFN4-null Golgi is incapable of forming proper proacrosomal granules. At present the data does not directly show this.
9. In the discussion the authors suggest that the male infertility is primarily driven by defects in acrosome biogenesis in part based on the results of the zona free fertilisation experiments. However, this experiment only rescues the development to morula stage by about 30%, which would suggest there are other key factors impacting the fertility of the PFN4-null males. Or does this interpretation take into account reduced sperm viability as a result of acrosome abnormalities?
10. The method used for germ cell isolation needs to be included.
11. For acrosome reaction, were dead sperm removed from analysis?

Reviewer 2

Advance summary and potential significance to field

The authors generated PFN4-deficient mice and studied the role of the gene in sperm formation and male fertility. They discovered that the homozygous males were infertile associated with structural defects in the cis- and trans-Golgi networks and aberrant acrosome biogenesis as well as abnormal manchette formation. Proteomic analysis revealed increased protein levels of some proteins involved in Golgi membrane trafficking and the PI3K/AKT pathway and an inhibition of autophagy. They concluded that hat PFN4 plays a role in pro-acrosomal vesicle formation and trafficking by maintaining the structural integrity of the Golgi network for acrosome biogenesis through autophagy regulation. and that impaired manchette development explaining the abnormal sperm nuclear morphology.
The research is novel, and the data are solid. The study identified another key gene essential for acrosome formation and therefore is highly significant.

Comments for the author

1. How about sperm motility?
2. The manchette defects normally suggest sperm flagella defects. The authors should provide high magnification of sperm images and TEM images of sperm flagella structure;
3. The quality of most Western blots was not acceptable. A statistic analysis showing difference should be conducted. It would be also interesting to compare the localization of some proteins, like ARF3 and AKT1 between the control and KO mice.
4. The authors examined the Golgi bodies/acrosome and the manchette using the specific antibodies against the key structure components. How about other proteins localized at these structures normally in the wild-type mice in the KO mice? It would be more supportive if some examples can be provided.
5. How about profilin 3 expression in the Ko mice.
Minor.
1.Page 10. It is confusion: Eleven proteins were differentially abundant when comparing Pfn4-/- samples to Pfn4+/- samples, however, significantly differentially abundant proteins were not detected in Pfn4-/- samples compared to the WT (Fig. S2 B). Please clarify;

© 2022. Published by The Company of Biologists under the terms of the Creative Commons Attribution License (https://creativecommons.org/licenses/by/4.0/).

2. More details should be provide for the Acrosomal reaction. Foe example, how to evaluate the sperm with acrosome reaction;
3. Make sure all the references are correct.

Reviewer 3

Advance summary and potential significance to field

Both Pfn4 and Pfn3are actin monomer binding molecules. Umer et al. generated Pfn4-deficient mice by the CRISPR-Cas9 system and analyzed the phenotype of the mice. They made two lines with an identical gene deletion at the Pnf4 allele. They found that Pfn4-deficient spermatids revealed an impairment of proacrosomal granules at Golgi apparatus inhibition of autophagy, formation of the amorphous head shape, the fragmented Golgi apparatus, and a reduction of sperm viability. The IVT experiments with Pfn4-deficient sperm showed abnormal development of the fertilized eggs. In sum, they reported functions of Pfn4 possibly in autophagy-mediated pro-acrosomal vesicle formation and trafficking for acrosome biogenesis.

Comments for the author

Actin is essential during sperm differentiation at various places in spermatid and sperm such as acrosome matrix, acroplaxome, manchette and tail. There are several possible molecular mechanisms that explain the abnormalities observed in the KO mice. Thus, a more comprehensive understanding of Pfn4 deficiency and more in-depth analyses in spermatid and sperm are necessary to conclude whether the molecular mechanism proposed here is correct or not. For example, the KO mice revealed deformed elongated spermatids and dead sperm in testes. Therefore, although it is known that autophagy-related molecules participate in acrosome biogenesis, it is likely that the cells that are undergoing cell death are present in the testes of the KO mice. Also, autophagy can occur through both mTOR-dependent and -independent pathways. The authors should carefully distinguish them and characterize them.
In addition, despite that the same group previously generated Pfn3-deficient mice and reported the phenotype, the results presented in this paper, unfortunately, do not significantly advance our previous understanding of the function of Pfn3/4 molecules in spermatogenesis.

Major comments:
1. The title should be changed. Although the PI3K/AKT/mTOR related proteins are upregulated in the total cell lysate western blotting, the authors do not show the change in PI3K/AKT/mTOR directly causes inhibition of autophagy in spermatogenic cells.
2. Please show PFN4 protein localization by TEM in developing spermatids during acrosome biogenesis. Also, please indicate the histology data of the epididymis and statistically evaluate the number of sperm to see if the KO mice have less sperm.
3. Figure 1. They set up two mouse lines with an identical gene deletion, Δa and Δb. The description of these two lines is very confusing.
Fig1C-F: Are these data for line Δa? In other words, do the labels +/- and -/- mean Δa?
S1A: Does this show the result of screening for identification of founder mice? Or does this figure show genotyping of the Δa line?
4. The KO mice revealed a low percentage of viable sperm. Is the apoptosis/cell death frequency in the round and elongated spermatids in the KO testes higher than that in the control mice?
5. The authors pointed out the misshaped and smaller nuclei in elongated spermatids. These abnormal spermatids should be phagocytosed by Sertoli cells. Please check it by TEM.
6. Figure 2. How many mice did they use? How many independent experiments did they perform?
7. Figure 2C. Please show the data on which these plots are based.
8. Figure 4M, 4P. What do the arrows in these figures indicate? In addition, please specify the place where the trans-Golgi network does not gather to fuse, and a failure of the cap-like structure formation. Much larger magnification figures will help to understand the observation.
9. Figure 4. Whether the observed staining image of the Golgi apparatus is due to a failure of fusion must be firmly distinguished from fragmentation. Actin cytoskeleton network is necessary for correct Golgi positioning, architecture and trafficking. It is also known that fragmented Golgi

© 2022. Published by The Company of Biologists under the terms of the Creative Commons Attribution License (https://creativecommons.org/licenses/by/4.0/).

apparatus is observed as an early apoptotic event due to disassembly of the Golgi apparatus. Stabilizing actin filaments causes an extensive fragmentation of Golgi. Thus, please analyze apoptosis and actin cytoskeleton network carefully.
10. Figure 5. Regarding TGN46 staining, there are many small dots in the cytoplasmic regions far from the nucleus. Please explain what they are. Why is the number of TGN46 signals increased depending on the Pfn4 gene dosage? Other trans-Golgi markers should be tested, such as TGN38 co-stained with LC3 and p62. This is also necessary for the validation of the western blot analyses. Is a phosphorylated form of p62 also increased?
11. In addition, please check other vesicle formation and trafficking molecules. The following molecules, ATG7, Beclin1, GOPC and PICK1, are candidates for staining.
12. Figure 5. There are mTOR-independent autophagy pathways. Please check whether Ca2+, JNK, ROS and HIF-1a signalling pathways are activated or not.
13. Figure 6. Since the mTOR signal is activated and autophagy is inhibited, LC3II and p62(SQSTM1) are accumulated in the KO mice as pointed out by the authors. However this could lead to autophagy cell apoptosis (ACA) and causes apoptosis in spermatids in the KO mice.
14. Figure 6. It is a bit surprising that LC3I migrates faster than LC3II.
15. Figure 6. The western blotting for AKT1 should be improved.
16. Figure 6. The authors claimed imbalance in PI3K/AKT/mTOR leads to inhibition of autophagy and cited Xu's paper (Xu et al. 2020). However, the paper the authors cited has been retracted.
17. Figure 7/Sup Figure 3. To test whether the infertility of the KO mice is mainly due to an acrosome defect, please employ intracytoplasmic sperm injection, too. The statistical data of the IVH experiments should be provided.
Minor comments:
Figure 1. Please mention that E&N staining and hypo-osmotic swelling test were done using cauda epididymis sperm.

First revision

Author response to reviewers' comments

Dear Reviewers,

We like to thank you for your comments and input and patience in dealing with our data regarding the PFN4 ko mice. As you can see from the ms and the rebuttal letter, we have made many changes, performed further experiments, added data and re-interpret the results in light of the reviewers input. We think that the ms is now greatly improved.

Thanks again Hubert Schorle

Reviewer 1 Advance Summary and Potential Significance to Field:
Umer et al have characterised the role of profilin 4 (PFN4) in spermatogenesis and male fertility using a Pfn4 knockout (Pfn4-/-) mouse. Profilins are actin binding proteins that have roles in regulating actin dynamics, and a range of cellular processes including membrane trafficking, cytokinesis and cell motility. Here, the authors reveal that PFN4 is essential for male fertility in the mouse and show that a loss of PFN4 results in head shaping and acrosome abnormalities. Towards understanding the mechanism for the acrosome abnormalities, they show that there is dysregulation of key components of the autophagy and Golgi transport pathways.

Overall the study clearly shows an important role for PFN4 in male fertility, and will be of interest to researchers in the male reproductive biology field.
However, characterisation of the specific aspects of the acrosome and manchette phenotypes need to be strengthened and go into much more depth to ensure the data and interpretations are convincing.

© 2022. Published by The Company of Biologists under the terms of the Creative Commons Attribution License (https://creativecommons.org/licenses/by/4.0/).

Reviewer 1 Comments for the Author:
Specific comments:

Reviewer's comment: 1.The basic characterisation of the male fertility phenotype in Fig 1 needs to be improved.a.Testis and body weight are independent of body size and thus should not be expressed as a ration of body weight. b.The histological characterisation of the testis needs to re-done. Testes should be stained with Periodic Acid Schiff's stain and hematoxylin (not hematoxylin and eosin) to allow proper stage specific characterisation of the seminiferous epithelium (this will also provide good insight on acrosome formation as PAS stains the acrosome and proacrosomal vesicles so when there is a defect in vesicle trafficking it is quite apparent). Higher magnification images should also be used to point out the abnormal shaped sperm heads, they are quite hard to discern in the current Fig 1G images.

Author's response: We added more details about the basic characterization. We recalculated the absolute weights of Cauda epididymis, Testes. Further data are presented in Supp Fig 1, E-H. Further, histological characterization of testes using PAS staining has been done and is presented in Fig. 1D as higher magnification images.

Reviewer's comment: Sperm motility should be assessed.

Author's response: The sperm motility was assessed using OPEN-Casa and presented in table 1. The section reads now (line 222-228)

Reduced sperm motility in *Pfn4*$^{-/-}$ mice
Flagellar deformities might affect the sperm motility parameters, so, we performed computer assisted semen analysis (CASA), to analyze the swimming properties of *Pfn4*$^{-/-}$ sperm. Compared to *Pfn4*$^{+/+}$ and *Pfn4*$^{+/-}$, sperm motility parameters (curvilinear velocity (VCL), straight-line velocity (VSL), average path velocity (VAP) progressive and total motility) are significantly reduced in *Pfn4*$^{-/-}$ sperm (Table 1). These results indicated that abnormalities in sperm flagella led to the reduced overall sperm motility in *Pfn4*$^{-/-}$ mice.

Reviewer's comment: 2. The authors show that sperm head shaping is disrupted in the Pfn4-/- mice. Towards understanding the origin of this phenotype they stain isolated germ cells for alpha tubulin to look at manchette development and dynamics. From the data presented it appears that no manchettes from in the Pfn4-/- mice which is a really interesting finding however this data needs to be strengthened for it be convincing. The finding should be confirmed by electron microscopy to look at MT cytoskeleton ultrastructure and/or IHC staining of testis sections for alpha tubulin so that the spermatids can be properly staged. Indeed, given that head- shaping is abnormal this will impact accuracy of staging of isolated germ cells in the Pfn4-/-.

Author's response: We agree with the reviewer and have performed more experiments and re-arranged the manuscript and in order to further analyze this defect. Now, MT ultrastructure is shown in supplementary Fig. S2A, and IHC staining of alpha tubulin on testes section is given in supplementary Fig. S2B.

Reviewer's comment: Also need to more precisely define in text how the manchette is disrupted.

Author's response: A description of the disrupted manchette is included in the text. We added the following sentences (see lines 171-191):

Alpha tubulin staining demonstrated, that *Pfn4*$^{+/-}$ and WT spermatids showed proper manchette development and structural organization as a tight junction of manchette microtubular array of the caudal region of spermatids (Fig. 3 A, B). In contrast, in *Pfn4*$^{-/-}$ mice, did show only marginal staining for alpha tubulin, presenting as punctate and dispersed. Hence, the developing *Pfn4*$^{-/-}$ spermatids completely lack the typical parallel microtubular array structure of the manchette (Fig. 3 C).

In order to further detail this defect on morphological level, we used TEM to analyze the ultrastructure. *Pfn4*$^{-/-}$ spermatids showed mis-localized (step 8-9) and angular shape (step 10) microtubular manchette, while in *Pfn4*$^{+/+}$ spermatid microtubular manchette is properly aligned

© 2022. Published by The Company of Biologists under the terms of the Creative Commons Attribution License (https://creativecommons.org/licenses/by/4.0/).

(step 8-9) and crescent shape (step 10) in the middle of posterior region of developing spermatid (Fig. S2 A). The Manchette defect was more prominent in later steps (11-13) of development. In step 11-13, *Pfn4*$^{-/-}$ spermatids lack the microtubular array structure, it appears scraggy and rugged in shape, compared to *Pfn4*$^{+/+}$ spermatids, who showed the microtubular mantle aligned in arrays (Fig. S2 A). These data indicate that deletion of PFN4 disrupts manchette formation, which, in consequence leads to the deformed sperm heads observed in (Fig. 2 C and S2 A).

Furthermore, we performed IHC using alpha tubulin antibody on testes sections of WT, *Pfn4*$^{+/-}$ and *Pfn4*$^{-/-}$. IHC showed abnormal staining from step 8-11 in *Pfn4*$^{-/-}$ spermatids (indicated by stars), compared to WT and *Pfn4*$^{+/-}$ testes section, IHC showed properly formed sickle shape microtubular manchette on posterior region of developing spermatids. Further, in steps 12-16, in *Pfn4*$^{-/-}$ spermatids complete loss of manchette and amorphous shape of elongated sperm heads are observed (indicated by stars), compared to WT and *Pfn4*$^{+/-}$ spermatids (Fig S2 B).

Reviewer's comment: 3.The authors show that Pfn4-/- mice have a high percentage of dead epididymal sperm, in addition they show that a high percentage of Pfn4-/-epididymal sperm have an amorphous and smaller head shape. Is it possible that these two things are related i.e. that the small amorphous shape is just due to the dead sperm becoming pyknotic as they degrade? Is it possible to characterise the head shapes of just the viable sperm to exclude the possibility that that the defect seen is just due to cell death?

Author's response: We reworded and re-analyzed the dataset. We have observed smaller heads in PAS stained testes sections shown in Fig. 1D and supplementary Fig. S3A. However, we think that the defects in the manchette observed by us is the reason of the amorphous (roundish) head shape seen in Fig 2, B. Further, we would like to mention is not possible to characterize the shape of live sperm only, since we used E&N stain and Hos test to assess sperm viability. The software used for analyzing sperm shape requires DAPI staining after fixing and washing with formaldehyde. There, we cannot discern between live and dead sperm. Of note, smaller sperm head shapes are shown in Supplementary Fig. 3D, S2B, S2C, and S3A.

Reviewer's comment: In Fig 4 the authors report that acrosome biogenesis is disrupted. However, while the EM data is clear, in the IF data panels A-I is not convincing for showing a defect in acrosome biogenesis. It is unclear what is meant by non- uniform acrosome vesicle for the in text description of Fig 4C – non uniform in shape, or thickness or gaps in it? Overall, the KOs look normal particularly in 4C and in panels F it appears the spermatids are at an earlier stage than panels D and E. Similarly, it is hard to tell if Panel I is just an earlier stage than G and H or if is abnormal. Assessing PAS-stained sections may make the data more convincing and will help make seminiferous tubule stage more clear.

Author's response: PAS-staining was performed and added in the Supplementary Fig. S3A (in addition to PAS staining shown in Fig 1 D.
Since Behnen et al. demonstrated that PFN4 is located in to the acroplaxome, we think that what we are detecting in terms of disturbance of the acrosome is in fact a primary defect of the lack of PFN4 here. We cannot rule out the fact, that the disturbed and rather chaotic development of the manchette has an influence on the genesis of the acrosome as well. We re-worded the paragraph from 237 -248 it now reads:

In seminiferous tubules of WT (Fig. 5 A) and *Pfn4*$^{+/-}$ (Fig. 5 B) male mice the developing acrosome forms a single homogenous cluster present on the anterior face of nuclei in Golgi phase spermatids, while in *Pfn4*$^{-/-}$ spermatozoa PNA-FITC staining showed a non-uniform shape of the acrosomal vesicle (Fig. 5 C). This abnormal acrosome development was further seen in the next step, known as the Cap phase. Here, a cap-like covering can be seen on the apical surface of the WT and *Pfn4*$^{+/-}$ spermatozoa (Fig. 5 D, E). In *Pfn4*$^{-/-}$ sperm cells the cap like structures were partially impaired (white arrows) (Fig. 5 F). In the Acrosomal phase, where elongation and head remodeling of spermatids starts, this abnormal acrosome development was most prominent. In *Pfn4*$^{-/-}$ sperm the acrosome structure failed to develop into an arrow-like structure (Fig. 5 I), as seen in the WT (Fig. 5 G) and *Pfn4*$^{+/-}$ sperm (Fig. 5 H). These suggest that loss of PFN4 partially impairs the biogenesis of acrosome starting from the first phase (Golgi phase) of acrosome development. Further, PNA-FITC labelling on mature sperm isolated from cauda epididymis of *Pfn4*$^{-/-}$ mice showed malformed

© 2022. Published by The Company of Biologists under the terms of the Creative Commons Attribution License (https://creativecommons.org/licenses/by/4.0/).

acrosome, and in addition, aberrant head morphology (Fig. 5 J). In addition, PAS (periodic acid-Schiff) staining further confirms the abnormalities in acrosome development (Fig. S3 A).

Reviewer's comment: 5. In Fig 4 the EM pictures are nice however, even in the source file they are quite pixelated please fix for the final submission – in particular it would be good to be able to discern the detail of the acroplaxome and marginal ring. Also, the Fig 4 EM panels needs to be clarified. In particular the authors state that for Fig 4m ' in Pfn4-/- mice, the proacrosomal vesicles released from trans-Golgi network do not seem to gather and fuse on the apical face of developing spermatozoa, suggesting that the acrosomal vesicle did not form properly'. However in subsequent stages they show that acrosome does form albeit abnormally. Would this not suggest that this process is just delayed as opposed to failing completely?

Author's response: Pixels are fixed, details of acroplaxome and marginal ring is highlighted in text and figure 5. We agree with the reviewer and added a sentence according to the reviewer's suggestion (lines 252-256):

In *Pfn4*$^{-/-}$ mice, the proacrosomal vesicles released from *trans*-Golgi network do not seem to gather and fuse as a single large and dense acrosomal vesicle on the apical face of developing spermatozoa (Fig. S3 B) This suggests that the acrosomal vesicle formation is greatly delayed retarding acrosome biogenesis (Fig. 6 C, arrow indicates large size acrosomal vesicle) in subsequent phases.

Reviewer's comment: 6. Fig 5 – Please clarify how the cis-Golgi staining shows that it is a non-polarised stack in Pfn4-/-.

Author's response: The term "polarized/ oriented stack" means cisterna of cis-Golgi is running nicely and uniformly along the nucleus, if the cisterna stack is not aligned uniformly along the nucleus then it is called non-polarized or dis-oriented. This was described previously by Bisel et al., 2013. However, for the ease of understanding, we have rephrased the sentence (lines 284-287):

In *Pfn4*$^{-/-}$ testis sections, structural disruption and mild mis-localization of *cis*-Golgi network is observed, and *trans*-Golgi is fragmented and dispersed throughout the cytoplasm indicating impaired vesicle trafficking and transport unlike in WT and *Pfn4*$^{+/-}$ spermatids, where *cis- and trans-*Golgi are properly formed and aligned.

Reviewer's comment: 7. Fig 8 – needs more clarification in the figure legend. Is 1-cell effectively ones that haven't fertilised?

Author's response: We reviewed the bar-graph and decided to display the data in a new and easier format. The old Fig. 8 is now updated now displayed as Fig. 9C. Here we display the number of zygotes which developed to blastula and morula stages, since these are the only ones, which are informative in a way that they are fertilized and show, that zona-free oocytes can be fertilized by Pfn4 deficient sperm. As we were looking at the data, however, it appeared to us, that a comparison (and presumably discussion) of the percentages of fertilized oocytes using PFN4 deficient sperm with WT sperm using oocytes with zonae might lead to a misinterpretation. To be correct, one should have used zona-free Oocytes and WT sperm as well to consider that the treatment to remove the zonae (Acid Tyrodes) might affect overall oocyte viability as well. This comment relates to Reviewers comment #9. The section now reads (line 354 – 362):

***Pfn4* deficient sperm are able to fertilize zona-free oocytes**
The acrosome contains digestive enzymes which degrade the zona pellucida and enable the fusion of the sperm with the egg (Saldívar-Hernández et al., 2015). Since the PFN4-deficient sperm display defects in acrosome function, we performed IVF (in-vitro fertilization) on oocytes with and without zona pellucida using *Pfn4*$^{-/-}$ sperm. While *Pfn4*$^{-/-}$ sperm was not capable of fertilizing oocytes with zonae, using zona-free oocytes resulted in successful fertilization since 30% developed to morula/blastocyst stage (Fig. 9 C). In comparison, 70% of oocytes were successful fertilized using wildtype sperm. This indicates that the acrosome defects in *Pfn4*$^{-/-}$ sperm contribute to male infertility of the PFN4-deficient mice.

Reviewer's comment: 8. In the discussion the authors state that the PFN4-null Golgi is incapable of forming proper proacrosomal granules. At present the data does not directly show this.

© 2022. Published by The Company of Biologists under the terms of the Creative Commons Attribution License (https://creativecommons.org/licenses/by/4.0/).

Author's response: We agree with the reviewer, "forming proper proacrosomal granule" is replaced now with "forming a proper acrosomal vesicle" given in lines from 384-386, it read now:

Defects in acrosome biogenesis seems to originate from a malfunctioning Golgi network, which is incapable of forming a proper acrosomal vesicle.

Reviewer's comment: 9. In the discussion the authors suggest that the male infertility is primarily driven by defects in acrosome biogenesis in part based on the results of the zona free fertilisation experiments. However, this experiment only rescues the development to morula stage by about 30%, which would suggest there are other key factors impacting the fertility of the PFN4-null males. Or does this interpretation take into account reduced sperm viability as a result of acrosome abnormalities?

Author's response: Yes, we agree with the reviewer, besides acrosomal defects, amorphous sperm heads due to defective manchette, reduced sperm motility and flagellar malformations together contribute to the infertility seen in PFN4 deficient mice. We have changed the text accordingly (lines 399 - 400).

The infertility seems to be mainly caused by perturbation of manchette development and acrosome biogenesis.

Reviewer's comment: The method used for germ cell isolation needs to be included.

Author's response: Method for germ cell isolation is given in the material and methods section (line 605-613).

Isolation of Germ cell population from testes

Testes were collected in ice-cold PBS and tunica albuginea was removed carefully. Testicular tissue was minced in 200 µl of digestion medium until all seminiferous tubules seemed to be digested. Then 800 µl of remaining digestive medium was added and incubated for 25 min at 37 °C with slow continuous rotation. Additional mechanical disruption was performed by pipetting the solution for 5 times using recovery tips. Suspension was filtered in 35mm pore size filter to achieve single cell suspension. Cell suspension was pipetted slowly to avoid clogging and cell damage. Cells were collected by centrifuge for 10 min at 37 °C on 400g, supernatant was discarded, and pellet was resuspended in 1 mL of PBS (Umer et al., 2021).

Reviewer's comment: 11. For acrosome reaction, were dead sperm removed from analysis?

Author's response: For acrosomal reaction, dead sperm were not removed from the analysis because we used a calcium ionophore to induce the acrosomal exocytosis. It induces the rapid influx of calcium in cells, leading to exocytosis. Dead sperm cells can't increase the calcium flux in cells and therefore did not participate in this reaction. In addition, acrosomal content is lost in dead sperm cell by rupturing of membrane and they will not undergo the acrosomal reaction. So, removing of dead cells from the analysis will not affect the overall percentage of acrosomal reacted sperm in *Pfn4*$^{-/-}$ mice.

Reviewer 2 Advance Summary and Potential Significance to Field:
The authors generated PFN4-deficient mice and studied the role of the gene in sperm formation and male fertility. They discovered that the homozygous males were infertile associated with structural defects in the cis- and trans-Golgi networks and aberrant acrosome biogenesis as well as abnormal manchette formation. Proteomic analysis revealed increased protein levels of some proteins involved in Golgi membrane trafficking and the PI3K/AKT pathway and an inhibition of autophagy. They concluded that hat PFN4 plays a role in pro- acrosomal vesicle formation and trafficking by maintaining the structural integrity of the Golgi network for acrosome biogenesis through autophagy regulation. and that impaired manchette development explaining the abnormal sperm nuclear morphology.
The research is novel, and the data are solid. The study identified another key gene essential for acrosome formation and therefore is highly significant.

© 2022. Published by The Company of Biologists under the terms of the Creative Commons Attribution License (https://creativecommons.org/licenses/by/4.0/).

Reviewer 2 Comments for the Author:
Reviewers comment: 1. How about sperm motility?

Author's response: We performed sperm motility analyses using OpenCASA and present the results in table 1, the paragraph now reads (line 222 – 228):

Reduced sperm motility in *Pfn4$^{-/-}$* mice
Flagellar deformities might affect the sperm motility parameters, so, we performed computer assisted semen analysis (CASA), to analyze the swimming properties of *Pfn4$^{-/-}$* sperm. Compared to *Pfn4$^{+/+}$* and *Pfn4$^{+/-}$*, sperm motility parameters (curvilinear velocity (VCL), straight-line velocity (VSL), average path velocity (VAP) progressive and total motility) are significantly reduced in *Pfn4$^{-/-}$* sperm (Table 1). These results indicated that abnormalities in sperm flagella led to the reduced overall sperm motility in *Pfn4$^{-/-}$* mice.

Reviewers comment: 2. The manchette defects normally suggest sperm flagella defects. The authors should provide high magnification of sperm images and TEM images of sperm flagella structure;

Author's response: We checked the sperm flagella using MitoRed, SEM and TEM, found defects and included them in the manuscript. Results are given in Figure 4a, b, c, included in results section (lines 207-221) and in the discussion. Line 207-221 now read:

It has been reported, that during flagellar development sperm tail proteins are also delivered through the manchette, aside from the intraflagellar transport (IFT) (Mochida, Tres and Kierszenbaum, 1991), (Mendoza-Lujambio et al., 2002). Since loss of *Pfn4* disturbs development of the manchette, we next examined midpiece and flagellar structures in sperm cells. First, MitoRed dye was used to stain the sperm mitochondria in mid piece of flagella and DAPI was used to stain sperm heads. Here, *Pfn4$^{-/-}$* sperm revealed numerous deformities including i) cytoplasmic droplets in connecting piece, ii) bent mid-piece and abnormally thick mid-piece (Fig. 4 A). This suggests, that the disturbed manchette seen in *Pfn4$^{-/-}$* males, causes the mitochondria abnormalities in sperm flagellum.
Next, TEM was performed on cauda epididymis sperm. TEM on *Pfn4$^{-/-}$* sperm revealed disorganized flagellar components like i) cytoplasmic droplets in mid piece (black asterisk), ii) defective fibrous sheath (white star), and iii) mitochondria structural defect (black arrow) (Fig. 4 B). Scanning electron microscope (SEM) revealed morphological anomalies of *Pfn4$^{-/-}$* sperm including, i) coiled flagella, ii) shortened thick mid piece, iii) cytoplasmic droplets in mid piece and iv) bent connecting piece (Fig. 4 C).

Reviewers comment: 3. The quality of most Western blots was not acceptable. A statistic analysis showing difference should be conducted. It would be also interesting to compare the localization of some proteins, like ARF3 and AKT1 between the control and KO mice.

Author's response: We we performed WB again using AKT1 antibody. Further, we adjusted the background contrast of the remaining WB-images.
We added a statistical analysis of WB data and display the results in Supplementary fig. S5

Reviewers comment: 4. The authors examined the Golgi bodies/acrosome and the manchette using the specific antibodies against the key structure components. How about other proteins localized at these structures normally in the wild-type mice in the KO mice? It would be more supportive if some examples can be provided.

Author's response : We have performed additional IHC using antibodies to detect HOOK1 and ARL3 in order to further examine the structure of the manchette. The results are shown in Fig 3D and Supplementary data S2C. In addition, we antibodies towards GOPC, PICK1, and COPA to examine the Golgi structures, results are given in Supplementary data S4, A, B, C. Finally, antibody staining detecting SP56 and Acrosin were used to assess the acrosomal structure. The results are shown in Supplementary data S3 C, D for all three genotypes. The results were added in line 290-302:
Furthermore, we checked the localization of COPA (Coatomer *protein* complex subunit α), PICK1 (*Protein* Interacting with C Kinase - 1) and GOPC (Golgi Associated PDZ And Coiled-Coil Motif Containing) using IHC on testes sections of WT, *Pfn4$^{+/-}$* and *Pfn4$^{-/-}$* mice. COPA is essential for cargo

© 2022. Published by The Company of Biologists under the terms of the Creative Commons Attribution License (https://creativecommons.org/licenses/by/4.0/).

transport of Golgi derived proteins (Lepelley et al., 2020), PICK1 and GOPC play an important role for vesicle trafficking released from Golgi network and further associated with the proacrosomal granule formation (Xiao et al., 2009). Our IHC showed that COPA is mis-localized (arrow heads) in localized *Pfn4*$^{-/-}$ spermatids, indicating the disrupted vesicle transport for acrosome biogenesis while in in WT and *Pfn4*$^{+/-}$ developing spermatids COPA is localized in Golgi apparatus (black arrows) (Fig. S4 A). IHC using anti- PICK1 and anti-GOPC antibodies showed mis-localization of Golgi was observed in *Pfn4*$^{-/-}$ testes sections, while in WT and *Pfn4*$^{+/-}$ testes sections (black arrows) staining showed proper Golgi localization (Fig S4 B, C). These results further indicates that vesicle trafficking is affected in *Pfn4*$^{-/-}$ mice which ultimately result in abnormal formation of proacrosomal granules.

And line 410-426:

In addition, we observed the disrupted localization of HOOK1 and ARL3 in manchette of *Pfn4*$^{-/-}$ spermatids. Interestingly HOOK1 deficient males display a similar phenotype (impaired manchette and flagellar development, abnormal sperm head shape, reduced motility and malformed acrosome formation) as we observed in our *Pfn4*$^{-/-}$ males (Schwarz et al., 2017). Recently it has been reported that HOOK1 interacts with CCDC181 (coiled-coil domain containing protein 181) and plays a role in flagellar development and regulation (Schwarz et al., 2017). HOOK1 acts as cargo protein in IMT process for CCDC181 protein.
We have observed perinuclear ring staining using HOOK1, suggests that perinuclear ring formation is not affected in *Pfn4*$^{-/-}$ spermatids, however in later steps (10-13), we didn't observe the formation of microtubular manchette in *Pfn4*$^{-/-}$ spermatids. ARL3 plays a role in cargo transport for axoneme formation and is essential for ciliogenesis (Alkanderi et al., 2018). We speculate that PFN4 plays a role as structural and cargo protein, where it might participate in binding motor proteins and transport their cargo to specific locations during sperm development. We further hypothesize that PFN4 might interact directly or in a complex with HOOK1 and ARL3. Unfortunately, due to lack of PFN4-antibody, the exact localization of PFN4 protein (at ultrastructural level) in sperm flagellar cannot be known, in addition the Co-IP experiments cannot be performed to find the PFN4 interacting partners.

Reviewers comment: 5. How about profilin 3 expression in the Ko mice.

Author's response :We performed qRT-PCR on RNA extracts of testes for all three genotypes. We found that Profilin 3 expression is not affected in PFN4-deficient mice and is comparable to the levels detected in WT and Het mice. The data are shown below, we did not include this dataset in the MS. Please let us know if that is ok.

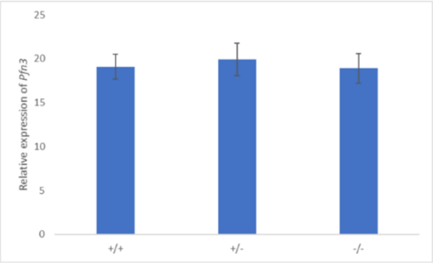

Minor.
Reviewers comment: 1.Page 10. It is confusion: Eleven proteins were differentially abundant when comparing Pfn4-/- samples to Pfn4+/- samples, however, significantly differentially abundant proteins were not detected in Pfn4-/- samples compared to the WT (Fig. S2 B).
Please clarify;

Author's response: A problematic aspect of our Mass-spec analysis is the greater variability within the wild-type group. This variability could explain why no significant proteins were found in the ko when compared to wild type. It can be assumed (just looking at the MS data) that the 3 groups (wt, het, hom) differ and that there is no intermediate phenotype for the het group and because of the low number of replicates (and the associated low power) only a few significant proteins were found and, in particular, none were found in the two comparisons with wild type.

© 2022. Published by The Company of Biologists under the terms of the Creative Commons Attribution License (https://creativecommons.org/licenses/by/4.0/).

Reviewers comment: 2. More details should be provide for the Acrosomal reaction. Foe example, how to evaluate the sperm with acrosome reaction;

Author's response: We performed additional experiments, rearranged the data and show them in Fig 9 a nd B. The section now reads (343 – 354):

Acrosomal reaction significantly reduced in *Pfn4*$^{-/-}$ sperm

As a result of the impaired acrosome biogenesis, we expected that the acrosomal reaction to be affected. To induce the acrosomal reaction, we used A23187 and Coomassie staining to differentiate the acrosome-reacted sperm (acrosome is not present on sperm head) (Fig. 9 A, black arrows) to non-acrosomal reacted sperm (acrosome is detectable as a crescent like shape (dark blue) on the sperm head) (Fig. 9 A, black arrowheads). Using the bright field microscope 200 spermatozoa in triplicates for each genotype is accessed. Acrosomal reaction was accessed by the absence of acrosome (a dark blue crescent shape on sperm head). Upon exposure to A23187, more than 72% of sperm from WT mice and 65% of sperm from *Pfn4*$^{+/-}$ mice showed acrosomal exocytosis, whereas only 5-6% sperm of the *Pfn4*$^{-/-}$ underwent acrosome reaction (Fig. 9 B). This finding demonstrates that sperm of *Pfn4*$^{-/-}$ males display significantly reduced acrosome reaction

Reviewers comment: 3. Make sure all the references are correct.

Author's comment: All the references are re-checked and corrected.

Reviewer 3 Advance Summary and Potential Significance to Field:
Both Pfn4 and Pfn3are actin monomer binding molecules. Umer et al. generated Pfn4- deficient mice by the CRISPR-Cas9 system and analyzed the phenotype of the mice. They made two lines with an identical gene deletion at the Pnf4 allele. They found that Pfn4- deficient spermatids revealed an impairment of proacrosomal granules at Golgi apparatus, inhibition of autophagy, formation of the amorphous head shape, the fragmented Golgi apparatus, and a reduction of sperm viability. The IVT experiments with Pfn4-deficient sperm showed abnormal development of the fertilized eggs. In sum, they reported functions of Pfn4 possibly in autophagy-mediated pro-acrosomal vesicle formation and trafficking for acrosome biogenesis.

Reviewer 3 Comments for the Author:
Actin is essential during sperm differentiation at various places in spermatid and sperm, such as acrosome matrix, acroplaxome, manchette and tail. There are several possible molecular mechanisms that explain the abnormalities observed in the KO mice. Thus, a more comprehensive understanding of Pfn4 deficiency and more in-depth analyses in spermatid and sperm are necessary to conclude whether the molecular mechanism proposed here is correct or not. For example, the KO mice revealed deformed elongated spermatids and dead sperm in testes. Therefore, although it is known that autophagy- related molecules participate in acrosome biogenesis, it is likely that the cells that are undergoing cell death are present in the testes of the KO mice. Also, autophagy can occur through both mTOR-dependent and -independent pathways. The authors should carefully distinguish them and characterize them.
In addition, despite that the same group previously generated Pfn3-deficient mice and reported the phenotype, the results presented in this paper, unfortunately, do not significantly advance our previous understanding of the function of Pfn3/4 molecules in spermatogenesis.

Author's comment: Win regard to the last paragraph written by the reviewer we would, with all due respect, like to mention, that we generated mice deficient for PFN3 and PFN4. While the PFN3 mice were reported elsewhere by us (Umer et al., 2021) in this manuscript, we report on generation and analysis of PFN4 defcient mice. The way we see it, previous knowledge was, that PFN3 and PFN4 are expressed during spermatogenesis. It was thought that the proteins *might* have a role during development of germ cells. But how and what exactly remained to be elucidated. With the PFN3 deficient mice (Umer et al 2021) and this manuscript we provide mouse models answering when and where the proteins are required, and what pathomorphological consequences arise upon loss of the proteins. As of today, to our knowledge, the exact biochemical role especially PFN4, which is regarded as the least Profilin-like profilin is not known. Our approach revealed sites of action, that is the manchette and the acrosome, which, to our opinion are the primary sites of action for PFN4. The manuscript does not claim to answer all questions regarding

© 2022. Published by The Company of Biologists under the terms of the Creative Commons Attribution License (https://creativecommons.org/licenses/by/4.0/).

PFN4 – however in its whole, it describes a lot of work and plenty data, which contribute to the knowledge of PFN4 and help understanding its role and its function. We believe, that the fact, that loss of PFN4 leads to significant disturbances of manchette and acrosome development which cannot be compensated by the remaining PFN1-3, represents, in itself, a major insight in protamine biology per se.

Major comments:
Reviewers comment: The title should be changed. Although the PI3K/AKT/mTOR related proteins are upregulated in the total cell lysate western blotting, the authors do not show the change in PI3K/AKT/mTOR directly causes inhibition of autophagy in spermatogenic cells.

Author's response: Title as been updated according to the suggestion of the reviewer and reads now: "PFN4 is required for manchette development and acrosome biogenesis during spermiogenesis."

Reviewers comment: 2. Please show PFN4 protein localization by TEM in developing spermatids during acrosome biogenesis .

Author's response: Behnen et al., 2009 and Obermann et al., 2005 showed that PFN4 is localised to the acrosome-acroplaxome-manchette complex. We purchased all commercially available ABs (from biorybt orb540926), Thermo-fischer (**PA5-89362), stJohns (STJ115647), and origene (TA321963). Using** WT protein lysates for WB, and sections of WT testes for detecting PFN4 using IHC, none of the ABs gave rise to a signal. Hence we were not able to validate the data published by Behnen et al., 2009 and Obermann et al., 2005. However, based on the defects we see in the PFN4 deficient mice we think that the published data reflect the location of the protein, that is the acrosome and manchette.

Reviewers comment: Also, please indicate the histology data of the epididymis and statistically evaluate the number of sperm to see if the KO mice have less sperm

Author's response: We performed histology of cauda epididymis and included the data in Fig. 1E. Further, by looking at the cauda epididymis histological sections in Fig. 1E, we conclude that sperm production is not affected in PFN4-deficient mice.

Reviewers comment: 3. Figure 1. They set up two mouse lines with an identical gene deletion, Δa and Δb. The description of these two lines is very confusing.

Author's response : In deleting a gene and generating a mouse, you might have unwanted off-target effects which hamper analysis and interpretation. In order to rule out such a situation, we always (numerous mice generated so far) generate and analyze at least two lines. In doing some analyses in parallel, we try to include this information as good as possible. In principle, what we like to say is, that we do have two lines which display the same phenotypical aberration. Not all experiments, were performed on both lines, though this would have been too expensive. We added a more detailed description in text and in Fig.1. (lines 132-139):

After generating and validating the PFN4-deficient lines, we performed fertility analysis on males from both *Pfn4*Δa and *Pfn4*Δb lines. Adult males heterozygous and homozygous for the *Pfn4* deletion mated normally with females as vaginal plugs were clearly seen. Males, heterozygous for the *Pfn4* mutation retain normal fertility, with average litter size comparable to WT littermates. This suggests that the reduced level of *Pfn4* mRNA observed in heterozygous males does not affect fertility. In contrast, *Pfn4*$^{-/-}$ mice of both *Pfn4*Δa and *Pfn4*Δb lines are infertile as none of the males (n=9) produced any pregnancy or offspring (Fig. 1 C, *Pfn4*Δa, Fig. S1 D, *Pfn4*Δb). Of note, female *Pfn4*$^{-/-}$ mice did not show any alterations in fertility (Fig. S1 E).

Reviewers comment: Fig1C-F: Are these data for line Δa? In other words, do the labels +/- and -/- mean Δa?

Author's response : Yes, phenotype of Δa line is indicated as +/- and -/- in main result part. However supplementary data also contains result for Δb as given in supplementary figure S1. Both lines have the same phenotype as shown in Fig 1 and supplementary Fig S1. By performing basic

© 2022. Published by The Company of Biologists under the terms of the Creative Commons Attribution License (https://creativecommons.org/licenses/by/4.0/).

analyses on two lines, we exclude the chance of falsely analyzing and reporting on phenotypes caused by off-target effects of CRIPSR mediated deletion of PFN4. After observing that both lines have the identical phenotype, we decided to run the detailed analysis on line Δa only.
Reviewers comment: S1A: Does this show the result of screening for identification of founder mice? Or does this figure show genotyping of the Δa line?

Author's response : Genotyping PCR in supplementary Fig. S1A are of the F1 generation, displayed are the results which identified the founder animals.

Reviewers comment: 4. The KO mice revealed a low percentage of viable sperm. Is the apoptosis/cell death frequency in the round and elongated spermatids in the KO testes higher than that in the control mice?

Author's response : In order to check apoptosis, we performed IHC staining for cleaved caspase-3, -9 and activated caspase-3 on testes sections and display the results now in supplementary Fig. S 7. There are no apoptotic sperm cells observed in PFN4 deficient testes sections which is comparable to WT and Het sections. We believe that low percentage of viable sperm are a consequence of abnormal acrosome and flagella structure. A section was added (line 371 – 378):

Apoptosis and phagocytosis are not observed in *Pfn4*$^{-/-}$ mice
To find whether apoptosis was induced in *Pfn4*$^{-/-}$ germ cells, IHC was performed using apoptotic markers, cleaved caspase-3, -9 and active caspase 3. Upon apoptosis induction, caspases are actively transported into nucleus and result in cell death. Interestingly, IHC showed no signal for germ cell death in *Pfn4*$^{-/-}$ testes, which is comparable to WT and *Pfn4*$^{-/-}$ sections (Fig. S 7 A). Further, we performed TEM analysis on testes section to find the phagocytosis of germ cells by Sertoli cells. Surprisingly, phagocytosis was not observed in *Pfn4*$^{-/-}$ Sertoli cells, which is comparable to WT and *Pfn4*$^{+/-}$ sections (Fig. S 7 B).

Reviewers comment: 5.The authors pointed out the misshaped and smaller nuclei in elongated spermatids. These abnormal spermatids should be phagocytosed by Sertoli cells. Please check it by TEM.

Author's response : We checked TEM images, and did not detect signs of phagocytosis of germ cells by Sertoli cells. We included this information in the text (line 376-378):
Further, we performed TEM analysis on testes section to find the phagocytosis of germ cells by Sertoli cells. Surprisingly, phagocytosis was not observed in *Pfn4*$^{-/-}$ Sertoli cells, which is comparable to WT and *Pfn4*$^{+/-}$ sections (Fig. S 7 B).

and line 511-513:

Interestingly, we didn't observe male germ cell apoptosis and phagocytosis by Sertoli cells in our PFN4-deficient mice. This suggest that inhibited autophagy caused by disrupted signaling pathways does not contribute to apoptosis in testicular germ cells of *Pfn4*$^{-/-}$ males.

Reviewers comment: 6.Figure 2. How many mice did they use? How many independent experiments did they perform?

Author's response : We performed 3 independent experiments and in total 3 mice/genotype were used.

Reviewers comment: 7.Figure 2C. Please show the data on which these plots are based.

Author's response : The data on which plots are based are appended below. This data are generated by the software that we used for nucleus shape analysis, after adding the DAPI stained sperm cells into it.

© 2022. Published by The Company of Biologists under the terms of the Creative Commons Attribution License (https://creativecommons.org/licenses/by/4.0/).

	WT	Het	Homo Cluster 1	Homo Cluster 2
Area mean 95% CI	20.13 ± 1.66	16.83 ± 1.44	15.03 ± 1.36	23.31 ± 0.70
Perimeter mean	21.34	19.58	21.41	27.9
Perimeter mean 95% CI	21.34 ± 0.92	19.58 ± 0.92	21.41 ± 1.31	27.90 ± 0.65
Circularity mean	0.53	0.53	0.43	0.4
Circularity mean 95% CI	0.53 ± 0.01	0.53 ± 0.01	0.43 ± 0.02	0.40 ± 0.01
Regularity mean	1.46	1.46	1.51	1.42
Regularity mean 95% CI	1.46 ± 0.02	1.46 ± 0.02	1.51 ± 0.03	1.42 ± 0.02
Bounding width mean	4.92	4.54	6.14	6.36
Bounding width mean 95% CI	4.92 ± 0.24	4.54 ± 0.23	6.14 ± 0.29	6.36 ± 0.12

Reviewers comment: 8.Figure 4M, 4P. What do the arrows in these figures indicate? In addition, please specify the place where the trans-Golgi network does not gather to fuse, and a failure of the cap-like structure formation. Much larger magnification figures will help to understand the observation.

Author's response : We added a description to the text. The arrows indicate the cap like structure which is not forming properly in PFN4-deficient testes line 239.

In *Pfn4*$^{-/-}$ sperm cells the cap like structures were partially impaired (white arrows) (Fig. 5 F). And in line 754-757:
Ultrastructural analysis using TEM on testes sections of WT, *Pfn4*$^{+/-}$ and *Pfn4*$^{-/-}$ mice. Golgi phase spermatozoa of (A) WT, (B) *Pfn4*$^{+/-}$ (C) *Pfn4*$^{-/-}$ mice (arrow indicates deformed developing acrosome). Cap phase spermatozoa of (D) WT, (E) *Pfn4*$^{+/-}$ and (F) *Pfn4*$^{-/-}$ mice (arrow indicates abnormal cap like structures).

Further, we added larger magnification images of *trans*-Golgi with proacrosomal granules. They are displayed in supplementary Fig. S3B.

Reviewers comment: 9.Figure 4. Whether the observed staining image of the Golgi apparatus is due to a failure of fusion must be firmly distinguished from fragmentation. Actin cytoskeleton network is necessary for correct Golgi positioning, architecture and trafficking. It is also known that fragmented Golgi apparatus is observed as an early apoptotic event due to disassembly of the Golgi apparatus. Stabilizing actin filaments causes an extensive fragmentation of Golgi. Thus, please analyze apoptosis and actin cytoskeleton network carefully.

Author's response : In order to check apoptosis, we performed IHC staining for cleaved caspase-3, -9 and activated caspase-3 on testes sections and added the results to supplementary Fig. S7A. There are no apoptotic sperm cells observed in the PFN4 deficient males. We believe that low percentage of viable sperm are because of abnormal acrosome and flagella structure. We further checked the actin cytoskeleton in testes sections for all three genotypes as presented in supplementary Fig. S6. It is comparable in all three genotypes.
However, we believe that fragmented Golgi is a consequence of lack of PFN4, which is localized there. PFN4 plays a role in stabilizing its structure and essential for vesicle trafficking. It has been already reported by Behnen et al., 2009 that PFN4 does not bind to actin, so we hypothesized that lack of this interaction does not alter the actin cytoskeleton.

© 2022. Published by The Company of Biologists under the terms of the Creative Commons Attribution License (https://creativecommons.org/licenses/by/4.0/).

Reviewers comment: 10.Figure 5. Regarding TGN46 staining, there are many small dots in the cytoplasmic regions far from the nucleus. Please explain what they are. Why is the number of TGN46 signals increased depending on the Pfn4 gene dosage? Other trans-Golgi markers should be tested, such as TGN38 co-stained with LC3 and p62. This is also necessary for the validation of the western blot analyses. Is a phosphorylated form of p62 also increased?

Author's response : These small dots are fragmented trans-Golgi dispersed throughout the cytoplasm as described previously by Cheng et al., 2011. Principally, the TGN46 signal is not increased, this is the fragmentation of *Trans*-Golgi and its parts are distributed in cytoplasm. We performed the LC3 and p62 IHC in on testes sections, which confirmed their higher abundance in Pfn4 knockout testes as given in Fig. 7D-E. In addition, some other Golgi markers such as COPA, PICK1, and GOPC showed mislocalization in PFN4 knockout testes sections which further confirms the disrupted Golgi structural organization as given in supplementary S4A-C. Since it was reported, that p62 acts as a marker for autophagosome formation, so we did not check its phosphorylation form.

Reviewers comment: 11.In addition, please check other vesicle formation and trafficking molecules. The following molecules, ATG7, Beclin1, GOPC and PICK1, are candidates for staining.

Author's response : In order to check the vesicle formation and trafficking from Golgi network, we performed IHC using antibodies detecting COPA, PICK1 and GOPC. The results are added to the ms and displayed in supplementary Fig. S4A-C. We added the results to the text (line 290 – 302).

Furthermore, we checked the localization of COPA (Coatomer *protein* complex subunit α), PICK1 (*Protein* Interacting with C Kinase - 1) and GOPC (Golgi Associated PDZ And Coiled-Coil Motif Containing) using IHC on testes sections of WT, *Pfn4*$^{+/-}$ and *Pfn4*$^{-/-}$ mice. COPA is essential for cargo transport of Golgi derived proteins (Lepelley et al., 2020), PICK1 and GOPC play an important role for vesicle trafficking released from Golgi network and further associated with the proacrosomal granule formation (Xiao et al., 2009). Our IHC showed that COPA is mis-localized (arrow heads) in localized *Pfn4*$^{-/-}$ spermatids, indicating the disrupted vesicle transport for acrosome biogenesis while in in WT and *Pfn4*$^{+/-}$ developing spermatids COPA is localized in Golgi apparatus (black arrows) (Fig. S4 A). IHC using anti-PICK1 and anti-GOPC antibodies showed mis-localization of Golgi was observed in *Pfn4*$^{-/-}$ testes sections, while in WT and *Pfn4*$^{+/-}$ testes sections (black arrows) staining showed proper Golgi localization (Fig S4 B, C). These results further indicates that vesicle trafficking is affected in *Pfn4*$^{-/-}$ mice which ultimately result in abnormal formation of proacrosomal granules.

Reviewers comment: 12.Figure 5. There are mTOR-independent autophagy pathways. Please check whether Ca2+, JNK, ROS and HIF-1a signalling pathways are activated or not.

Author's response : We performed IHC using 8-OHdG antibody on testes sections and didn't observe any sign of ROS mediated DNA damage in sperm of PFN4 knockouts. Since we did not detect apoptosis we did not check for JNK, HIF-1a and Ca2+. In addition, our enrichment analysis showed that proteins for PI3K/AKT pathway were enriched in Pfn4-deficient mice as data given in Supplementary Fig. S5D.

Reviewers comment: 13.Figure 6. Since the mTOR signal is activated and autophagy is inhibited, LC3II and p62(SQSTM1) are accumulated in the KO mice as pointed out by the authors. However, this could lead to autophagy cell apoptosis (ACA) and causes apoptosis in spermatids in the KO mice.

Author's response : In order to check the apoptosis, we performed the cleaved caspase-3, -9 and activated caspase-3 IHC staining on testes section as seen in supplementary Fig. S7A. There are no apoptotic sperm cells observed in Knockouts.

Reviewers comment: 14.Figure 6. It is a bit surprising that LC3I migrates faster than LC3II. Author's response : This was a mistake. LC3II migrates faster. We corrected this in Fig. 8C. Reviewers comment: 15.Figure 6. The western blotting for AKT1 should be improved.
Author's response : AKT1 WB is redone and presented in Fig. 8B.

© 2022. Published by The Company of Biologists under the terms of the Creative Commons Attribution License (https://creativecommons.org/licenses/by/4.0/).

Reviewers comment: 16.Figure 6. The authors claimed imbalance in PI3K/AKT/mTOR leads to inhibition of autophagy and cited Xu's paper (Xu et al. 2020). However, the paper the authors cited has been retracted.

Author's response : Thank you for pointing this out. Xu et al., 2020 retracted his paper because their research overlapped another publication (Wang MH and Lu JK). So, we replaced the reference with Li et al., 2018. The retracted citation has been replaced (line 85- 87).

mTOR is a main intracellular autophagic suppressor and positively regulated by PI3K/AKT pathway (Li et al., 2018).

Reviewers comment: 17.Figure 7/Sup Figure 3. To test whether the infertility of the KO mice is mainly due to an acrosome defect, please employ intracytoplasmic sperm injection, too.
The statistical data of the IVH experiments should be provided.

Author's response :
Upon comments from Reviewer 1 and 3, we reviewed the bar-graph showing the IVF data and decided to display the data in a new and easier format. The old Fig8 is now updated now displayed as Fig.9C. Here we display the number of zygotes which developed to blastula and morula stages, since these are the only ones, which are informative in a way that they are fertilized and show, that zona-free oocytes can be fertilized by Pfn4 deficient sperm. As we were looking at the data, however, it appeared to us, that a comparison (and presumably statistical discussion) of the percentages of fertilized oocytes using PFN4 deficient sperm with WT sperm using oocytes with zonae might lead to a misinterpretation. To be correct, one should have used zona-free Oocytes and WT sperm as well to consider that the treatment to remove the zonae (Acid Tyrodes) might affect overall oocyte viability as well. Since we did not have the resources (animal license, personell due to corona-related shortage of personell in this area of experiments) we decided to tone down the interpretation and discussion. We claim that the defect of the acrosome is not the only factor leading to infertility. Also, the amorphous head shape and flagellar defects are also contributing to the observed loss of fertility.
To our view ICSI would be mandatory in the case if IVF did not work. This would have indicated that the PFN4 sperm suffer from more damage than "only" disturbed acrosome formation.

Minor comments:
Reviewers comment: Figure 1. Please mention that E&N staining and hypo-osmotic swelling test were done using cauda epididymis sperm.

Author's response : We corrected the sentence, it reads now (line 147-148):
In order to assess the viability of spermatozoa, eosin and nigrosin (E&N) staining was performed on mature sperm cells isolated from cauda epididymis.

Second decision letter

MS ID#: DEVELOP/2022/200499

MS TITLE: PFN4 is required for manchette development and acrosome biogenesis during spermiogenesis.

AUTHORS: Naila Umer, Sharang Phadke, Farhad Shakeri, Lena Arévalo, Keerthika Lohanadan, Gregor Kirfel, Marc Sylvester, Andreas Buness, and Hubert Schorle

I have now received all the referees reports on the above manuscript, and have reached a decision. The referees' comments are appended below, or you can access them online: please go to BenchPress and click on the 'Manuscripts with Decisions' queue in the Author Area.

The overall evaluation is positive and we would like to publish a revised manuscript in Development, provided that the referees' comments can be satisfactorily addressed. Please attend

© 2022. Published by The Company of Biologists under the terms of the Creative Commons Attribution License (https://creativecommons.org/licenses/by/4.0/).

to all of the reviewers' comments in your revised manuscript and detail them in your point-by-point response. If you do not agree with any of their criticisms or suggestions explain clearly why this is so. If it would be helpful, you are welcome to contact us to discuss your revision in greater detail. Please send us a point-by-point response indicating your plans for addressing the referee's comments, and we will look over this and provide further guidance.

Reviewer 1

Advance summary and potential significance to field

Umer et al have characterised the role of profilin 4 (PFN4) in spermatogenesis and male fertility using a Pfn4 knockout (Pfn4-/-) mouse. Profilins are actin binding proteins that have roles in regulating actin dynamics, and a range of cellular processes including membrane trafficking, cytokinesis and cell motility. Here, the authors reveal that PFN4 is essential for male fertility in the mouse and show that a loss of PFN4 results in head shaping, acrosome and sperm tail abnormalities. Towards understanding the mechanism for the acrosome abnormalities they show that there is dysregulation of key components of the autophagy and Golgi transport pathways.

Overall the study clearly shows an important role for PFN4 in male fertility, and will be of interest to researchers in the male reproductive biology field.

Comments for the author

The researchers have addressed the majority of concerns from the original review.
A few comments below:
Line 29 - The authors should considers using a word other than 'retarded' which can be offensive
Line 47/48 - specify that you are talking about the timing of manchette formation in mice.
Line 52 – should it instead read - proteins are transported along manchette MTs by intramanchette transport? Current wording is not clear, need to clarify what the proteins are for.
Line 72 – 76: need to more precisely clarify where and when this process occurs during spermiogenesis
Line 144- 145 and Fig 1D – authors stare nuclei appear smaller however the KO is at a later stage than the WT and Het seminiferous tubules cross sections presented. The KO is stage XIII, just prior to sperm release and when nuclei have completed condensation. Whereas the WT and HET appear to be stage XII which is mid condensation when nuclei are still larger. I realise your other data supports a smaller nuclei phenotype, but these images need to be stage matched to conclude that from this.
Fig S2A – The quality of EM images makes differences had to discern. The contrast is low so MTs cannot be clearly seen. Collectively it appears from the images MT are present but are maybe not properly arranged around the nucleus at least in the later stages. Step 8 -/- panel: arrow appears to be pointing to an abnormal ectopic acrosome as opposed to a manchette? Step 8-9: -/- and +/+ panels appear comparable in terms of manchette. Step 10 -/- panel: arrow needs to be moved currently pointing within the nucleus. Step 12 –/- panel: arrow needs to be moved appears to pointing at the condensing chromatin, in this picture spears to be no MTs but hard to tell.
Line 202 – data do not suggest perinuclear ring is forming normally versus the microtubule mantle forming abnormally. Localisation of HOOK1 in Fig 3D, is consistent with localisation along the whole step 8-9 manchette structure. Data suggests manchette begins to form (including the microtubule mantle) but as head shaping progresses the manchette structure becomes progressively more abnormal and in some cases appears to be absent.

Reviewer 2

Advance summary and potential significance to field

The authors addressed all of my questions, and the quality of this manuscript has been significantly improved.

Comments for the author

The authors addressed all my issues.

© 2022. Published by The Company of Biologists under the terms of the Creative Commons Attribution License (https://creativecommons.org/licenses/by/4.0/).

Reviewer 3

Advance summary and potential significance to field

In this revised Manuscript, I found that the authors consequently improved the overall clarity of the paper.
The authors appropriately answered all the comments I previously raised.

Comments for the author

Minor comment
1. Please include the data for Figure 2C shown in the response as a supplemental table.

Second revision

Author response to reviewers' comments

Dear Dr. Koseki,

Thanks for the e-Mail and the information about your opinion on the manuscript. According to the reviewers comments, we have edited the have edited the manuscript and provide now a revised revised version. We also provide a letter where we answer to the reviewer's suggestions in a point-by-point manner. The new and edited sentences are highlighted in yellow in the manuscript.
We appreciate your patience with the story, thanks for handling our manuscript. Best wishes
Hubert Schorle

Reviewer 1 Advance Summary and Potential Significance to Field:
Umer et al have characterised the role of profilin 4 (PFN4) in spermatogenesis and male fertility using a Pfn4 knockout (Pfn4-/-) mouse. Profilins are actin binding proteins that have roles in regulating actin dynamics, and a range of cellular processes including membrane trafficking, cytokinesis and cell motility. Here, the authors reveal that PFN4 is essential for male fertility in the mouse and show that a loss of PFN4 results in head shaping, acrosome and sperm tail abnormalities. Towards understanding the mechanism for the acrosome abnormalities, they show that there is dysregulation of key components of the autophagy and Golgi transport pathways.

Overall the study clearly shows an important role for PFN4 in male fertility, and will be of interest to researchers in the male reproductive biology field.

Reviewer 1 Comments for the Author:
The researchers have addressed the majority of concerns from the original review. A few comments below:
Line 29 - The authors should considers using a word other than 'retarded' which can be offensive

Authors comment:
Line 29: the word was replaced by "disrupted"

Reviewer 1 Comment:
Line 47/48 - specify that you are talking about the timing of manchette formation in mice.

Authors' reply:
The sentence was edited to include the species (Line 47). In mice, formation of the caudal manchette starts during

Reviewer 1 comment:
Line 52 – should it instead read - proteins are transported along manchette MTs by intramanchette transport? Current wording is not clear, need to clarify what the proteins are for.

© 2022. Published by The Company of Biologists under the terms of the Creative Commons Attribution License (https://creativecommons.org/licenses/by/4.0/).

Authors' reply:
The sentence was edited, it now reads (Line 53-54):
Protein synthesis and storage occur in the cytoplasmic lobe and the proteins responsible for the strengthening of head-tail attachment are transported along manchette microtubules by intra-manchette transport (IMT).

Reviewer 1 comment:
Line 72 – 76: need to more precisely clarify where and when this process occurs during spermiogenesis

Authors' reply:
The following sentences were added to the ms (Line 74-77):
Autophagy is crucial for the proliferation and differentiation of spermatogonia stem cells, meiotic progression and spermiogenesis. During spermiogenesis, autophagy plays a role in acrosome development, head shaping, flagellum formation and cytoplasmic removal. During the process of autophagy,

Reviewer 1 comment:
Line 144- 145 and Fig 1D – authors stare nuclei appear smaller however the KO is at a later stage than the WT and Het seminiferous tubules cross sections presented. The KO is stage XIII, just prior to sperm release and when nuclei have completed condensation. Whereas the WT and HET appear to be stage XII which is mid condensation when nuclei are still larger. I realise your other data supports a smaller nuclei phenotype, but these images need to be stage matched to conclude that from this.

Authors' reply:
Figure 1 has been updated, Wt and het are now stage XIII as well.

Reviewer 1 comment:
Fig S2A – The quality of EM images makes differences had to discern. The contrast is low so MTs cannot be clearly seen. Collectively it appears from the images MT are present but are maybe not properly arranged around the nucleus at least in the later stages. Step 8 -/- panel: arrow appears to be pointing to an abnormal ectopic acrosome as opposed to a manchette? Step 8-9: -/- and +/+ panels appear comparable in terms of manchette. Step 10 -/- panel: arrow needs to be moved, currently pointing within the nucleus. Step 12 –/- panel: arrow needs to be moved appears to pointing at the condensing chromatin, in this picture spears to be no MTs but hard to tell.

Authors' reply:
Figure S2A has been edited, better contrast pictures are now shown, arrows are moved in Step 10 and 12 towards the manchette. In step 8, the arrow head is pointing towards the abnormal manchette, however, ectopic acrosome is visible on the anterior region of developing nucleus (in the main panel).

Reviewer 1 comment:
Line 202 – data do not suggest perinuclear ring is forming normally versus the microtubule mantle forming abnormally. Localisation of HOOK1 in Fig 3D, is consistent with localisation along the whole step 8-9 manchette structure. Data suggests manchette begins to form (including the microtubule mantle) but as head shaping progresses the manchette structure becomes progressively more abnormal and in some cases appears to be absent.

Authors' reply:
We edited the sentence, which now reads (line 207-209):
This suggests that the manchette starts to form (including the microtubule mantle), with the development of sperm head, the manchette structure becomes progressively more abnormal and, in some cases, appears to be absent.

Reviewer 2 Advance Summary and Potential Significance to Field:
The authors addressed all of my questions, and the quality of this manuscript has been significantly improved.

© 2022. Published by The Company of Biologists under the terms of the Creative Commons Attribution License (https://creativecommons.org/licenses/by/4.0/).

Reviewer 2 Comments for the Author:
The authors addressed all my issues.

Reviewer 3 Advance Summary and Potential Significance to Field:
In this revised Manuscript, I found that the authors consequently improved the overall clarity of the paper.
The authors appropriately answered all the comments I previously raised.

Reviewer 3 Comments for the Author: Minor comment

1.Please include the data for Figure 2C shown in the response as a supplemental table.

Authors' reply:
We added the table to the supplemental material and added a sentence to the main text (line 170): Details of parameters are given in Supplementary table 1.

<u>Third decision letter</u>

MS ID#: DEVELOP/2022/200499

MS TITLE: PFN4 is required for manchette development and acrosome biogenesis during spermiogenesis.

AUTHORS: Naila Umer, Sharang Phadke, Farhad Shakeri, Lena Arévalo, Keerthika Lohanadan, Gregor Kirfel, Marc Sylvester, Andreas Buness, and Hubert Schorle
ARTICLE TYPE: Research Article

I am happy to tell you that your manuscript has been accepted for publication in Development, pending our standard ethics checks.

© 2022. Published by The Company of Biologists under the terms of the Creative Commons Attribution License (https://creativecommons.org/licenses/by/4.0/).
